# An Efficient, Eco-friendly and Sustainable One-Pot Synthesis of 3,4-Dihydropyrimidin-2(1*H*)-ones Directly from Alcohols Catalyzed by Heteropolyanion-Based Ionic Liquids

**DOI:** 10.3390/molecules22091531

**Published:** 2017-09-11

**Authors:** Renzhong Fu, Yang Yang, Xudong Ma, Yu Sun, Jin Li, Hang Gao, Huaxing Hu, Xiaojun Zeng, Jun Yi

**Affiliations:** Jiangsu Laboratory of Advanced Functional Material, School of Chemistry and Materials Engineering, Changshu Institute of Technology, Changshu 215500, China; renzhongfujs@126.com (R.F.); 18852981193@163.com (X.M.); sy1885298@126.com (Y.S.); 18114760793@163.com (J.L.); 18020220376@163.com (H.G.); vsm6034@163.com (H.H.); zengxiaojun@cslg.edu.cn (X.Z.)

**Keywords:** Biginelli reaction, ionic liquid, microwave, oxidative cyclocondensation, solvent-free

## Abstract

Efficient, eco-friendly and sustainable access to 3,4-dihydropyrimidin-2(1*H*)-ones directly from alcohols under microwave and solvent-free conditions has been reported. The practical protocol involves heteropolyanion-based catalyzed oxidation of alcohols to aldehydes with NaNO_3_ as the oxidant followed by cyclocondensation with dicarbonyl compounds and urea or thiourea in a two-step, one-pot manner. Compatibility with different functional groups, good to excellent yields and reusable catalysts are the main highlights. The utilization of alcohols instead of aldehydes is a valid and green alternative to the classical Biginelli reaction.

## 1. Introduction

One-pot, sequential multi-step reactions have become an important area of research in organic chemistry, due to the improved atom economy, multiple-bond-forming efficiency, time and energy savings and avoiding waste and pollution [1,2,3,4,5]. Aldehydes are widely prevalent substrates in many efficient one-pot synthesis or multicomponent reactions (MCRs) [6,7,8]. However, they are generally very volatile, toxic and unstable, especially due to ease of aerial oxidation. In the practical process, aldehydes must be purified carefully just before use, otherwise the impurities will affect not only the concentration of the active aldehyde, but also the proceeding of the chemical reactions. It is apparent that these disadvantages have limited the application in industry. Recently, tandem oxidation processes (TOPs) in which oxidation of alcohols combined with the subsequent elaboration of the carbonyl intermediates (aldehydes) have gained considerable attention [9,10,11,12,13,14,15,16,17,18], while only a few reports described the combining alcohol oxidation with a MCR in a one-pot process [19,20]. Therefore, it is expected that the use of a single vessel oxidation-MCR protocol from alcohols would widen significantly the versatility and scope of aldehyde-based MCRs.

The century-old Biginelli reaction, which involves the one-pot condensation of an aldehyde, β-ketoester and urea or thiourea gives straightforward access to functionalized 3,4-dihydropyrimidin-2(1*H*)-ones (DHPMs) with a diverse range of biological properties [21,22,23,24,25,26], and is considered to be one of the most useful MCR [27,28,29,30,31]. Since the discovery of this reaction, a number of improved catalytic systems have been developed, such as Brønsted acids [32,33,34,35,36,37,38] or bases [39,40], metal Lewis acids [41,42,43,44,45,46,47,48,49,50], organocatalysts [51,52,53,54,55,56,57,58], and heterogeneous catalysts [59,60,61,62,63,64,65,66,67,68,69,70,71]. However, to the best of our knowledge, there have been only a few reports on the Biginelli reaction starting directly from alcohols [72,73,74,75].

With the common goal of being environmentally benign and allowing sustainable development in the chemical industry, the use of more eco-benign catalytic systems which are simple to recover helps to minimize waste production and maximize catalyst efficiency; this has been well studied in the past decades [76,77,78]. Among those, ionic liquids (ILs) have offered both economic and ecological benefits as ecofriendly and efficient alternatives to traditional organic solvents or catalysts [79,80]. In particular, a series of heteropolyanion-based ILs (HPAILs) [81,82,83], which were prepared by combining Keggin heteropolyanions with ‘task-specific’ IL cations containing special functional groups, are recently emerging as new species of hybrid materials [84,85]. In addition, reactions containing HPAILs are an attractive alternative for traditional acid-catalyzed [86,87,88,89,90,91] or oxidative [92,93,94,95,96,97,98,99] organic transformations because of their operational simplicity, lack of toxicity, ease of isolation and reusability. Therefore, HPAILs have proven to be a novel candidate for green and sustainable catalysts.

**Scheme 1:**
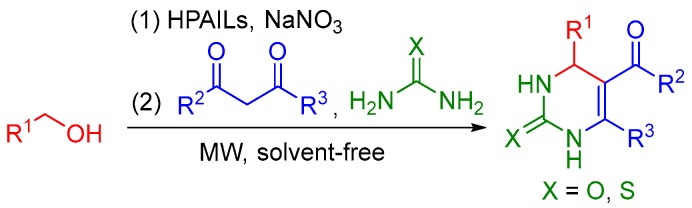
Tandem oxidation process (TOP) for the Biginelli reaction starting directly from alcohols.

Recently, a large number of publications have clearly shown that the main benefit of microwave (MW) chemical processing is the significant rate enhancements, yield and selectivity improvements as well as less environmental pollution, matching with the goals of green chemistry [100,101,102]. Following our continued investigation into the development of useful and sustainable synthetic methodologies [103,104,105,106,107,108,109,110,111]—along with recent investigations involving oxidation of alcohols and the subsequent trapping of carbonyl intermediates with appropriate nucleophiles in a one-pot operation. [112]—herein, we report an efficient and environmentally benign TOPs for the Biginelli reaction, starting directly from alcohols, using HPAILs as catalysts and NaNO_3_ as an oxidant under MW and solvent-free conditions (Scheme 1). Compared with existing reports [72,73,74,75], short reaction times and reusable catalysts are the main highlights of this methodology.

## 2. Results and Discussion

**Figure 1:**
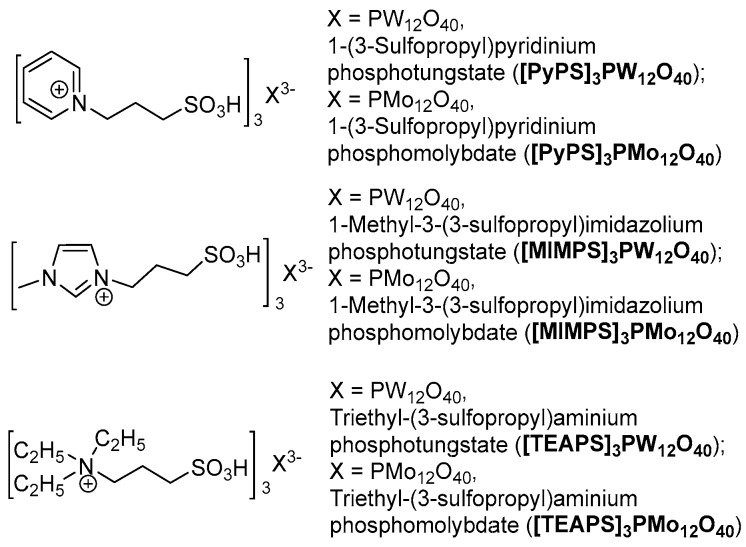
*N*-substituted imidazole, pyrdine and triethylamine based heteropolyanion-based ionic liquids (HPAILs).

Based on our previous investigations into HPAILs catalyzed reactions [103,104,105,106,107,108,109,110,111,112], *N*-substituted imidazole, pyridine and triethylamine based HPAILs were chosen as potential catalysts for this tandem oxidative cyclocondensation (Figure 1).

Initially, benzyl alcohol (3 mmol), ethylacetoacetate (3 mmol) and urea (4.5 mmol) were considered as standard model substrates to optimize the reaction conditions (Table 1). Firstly, we studied the tandem oxidative cyclocondensation using NaNO_3_ (3 mmol) as the oxidant in the presence of [PyPS]_3_PW_12_O_40_ (3 mol %) as catalyst at 80 °C under MW and solvent-free conditions in a one-step one-pot procedure, disappointingly the designed product was observed in only 13% yield even after long reaction times (Table 1, entry 1). However, to our delight, the DHPM was formed in 51% yield when ethyl acetoacetate and urea were added to the reaction mixture after the oxidation of alcohol to aldehyde was complete (Table 1, entry 2). Therefore, we aimed to accomplish the tandem oxidative cyclocondensation in a two-step one-pot manner. In order to improve the yield, some adjustments to the reaction conditions were made. It should be seen that higher temperature (100 °C) was harmful to the oxidation of alcohol due to the partial over-oxidation (Table 1, entry 3), whereas an obvious increased yield was obtained when the cyclocondensation was performed at higher temperature (100 °C and 120 °C) (Table 1, entries 4–5). In addition, more efficient results were observed by increasing the catalyst loading to 4 mol % and 5 mol % (Table 1, entries 6–7). Afterwards, the catalytic activities of other related catalysts prepared earlier were screened (Table 1, entries 8–12). It is expected that PyPS (1-(3-Sulfopropyl)pyridinium) species were found to be more efficient than MIMPS (1-Methyl-3-(3-sulfopropyl)imidazolium) and TEAPS (Triethyl-(3-sulfopropyl)aminium) species, while the results demonstrated that PW_12_O_40_ heteropolyanions were more active than PMo_12_O_40_ heteropolyanions. Although pure HPA (heteropolyacid) catalyst H_3_PW_12_O_40_ gave a moderate yield of 63%, its high solubility throughout organic solvents and water made its isolation from the reaction mixture difficult (Table 1, entry 13). Finally, an optimum result was obtained when the reaction was performed using 4 mol % of [PyPS]_3_PW_12_O_40_ at 80/120 °C under MW and solvent-free conditions affording DHPM in 92% yield (Table 1, entry 6).

To explore the scope and generality of this reaction, we extended the procedure to aromatic, heterocyclic, and aliphatic alcohols. In all cases, the reaction proceeded smoothly to afford the corresponding DHPMs within short reaction times (10–18 min) in good to excellent yields (71–95%) (Table 2). It could be noticed that a wide range of aryl alcohols bearing electron-withdrawing groups or electron-donating groups afforded good yields of DHPMs (Table 2, entries 1–7 and 11–28). Another important feature of this procedure is the compatibility with a variety of functional groups such as alkyl, alkoxyl, halide, nitro and hydroxyl under the present reaction conditions. Meanwhile, besides β-ketoesters, β-diketones (Table 2, entries 23–28) could also be employed with similar success to provide the corresponding products. In addition, the reactions with both urea and thiourea resulted in corresponding Biginelli adducts with good yields; nevertheless, longer reaction times were required in the cases of thiourea. It is worth mentioning that good yields were achieved (71–84% yield) in the cases of heterocyclic and aliphatic aldehydes (Table 2, entries 8–10), which normally were less reactive or completely inert in the Biginelli reaction.

**Figure 2:**
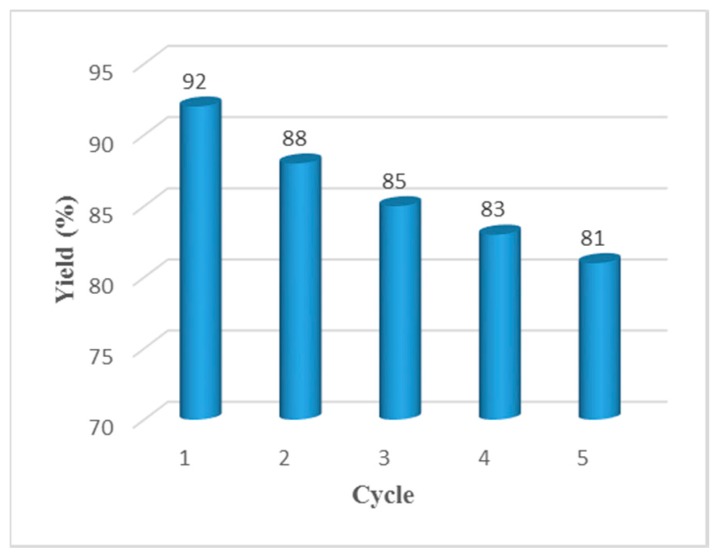
Reusability studies of the catalyst for the Biginelli reaction starting directly from alcohols.

In consideration of sustainable chemistry, the potential recycling of HPAILs was investigated with the reaction of benzyl alcohol, ethylacetoacetate and urea. Upon completion of the reaction in the first run, hot EtOAc was added to the reaction mixture in order to dissolve the final DHPM product. After vigorous stirring, the solid catalyst can be easily retrieved by simple filtration as well as washing with ethyl acetate and then ice water to remove traces of the previous reaction mixture and inorganic salt. After concentration of the filtrate, the almost pure product was obtained and recrystallization could be used for further purification. The recovered catalyst was used for further runs of the same reaction. As is evident from Figure 2, the reaction was repeated for up to five consecutive runs with a little loss of catalytic efficiency. Thus, the robustness of the catalyst and its reusability were demonstrated.

**Figure 3:**
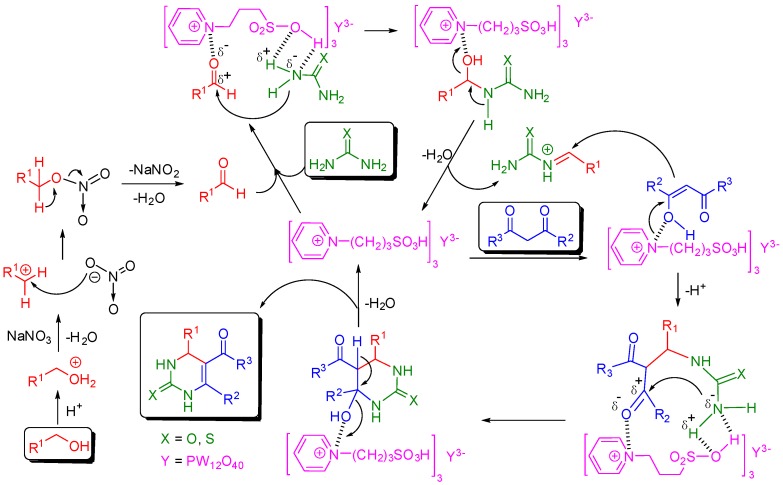
Plausible mechanism of HPAIL catalyzed Biginelli reaction.

Although the mechanism of Biginelli reaction has been a topic of much debate [27,28,29,30,31], the acidic proton and the cation of HPAIL appeared to play important roles in this tandem oxidative cyclocondensation based on the above discussions. Thus, according to the iminium route mechanism suggested by Folkers, Johnson and Kappe [113,114,115], a plausible mechanism for the reaction is depicted in Figure 3. Initially, the alcohol substrate was easily oxidized to aldehyde by NaNO_3_ with assistance of the acidic HPAIL catalyst. The catalytic cycle starts with the activation of the carbonyl of aldehyde by the coordination with an aminium cation and N–H activation of urea by the sulfonic group in the HPAIL cation. The adsorbed aldehyde-amine species undergoes an addition of the amine to the carbonyl carbon atom to obtain the dipolar adduct. After proton-exchange, desorption and dehydration, an imine intermediate is formed with a regenerated HPAIL catalyst. Meanwhile, the HPAIL cation stabilizes the enolization of 1,3-dicarbonyl compounds to form an enolate intermediate, which undergoes the Mannich-like addition with the imine intermediate followed by intramolecular cyclocondensation. Then, the final Biginelli product is obtained, resuming the HPAIL catalyst by proton-exchange, desorption and dehydration.

## 3. Materials and Methods

### 3.1. General Methods

Reagent grade solvents were used for extraction, recrystallization and flash chromatography. All other commercial reagents were used as received without additional purification. The progress of reactions was checked by analytical thin-layer chromatography (TLC, silica gel 60 F-254 plates) (Qingdao Haiyang Chemical Co., Ltd., Qingdao, Shandong, China). The plates were visualized first with UV illumination followed by iodine or phosphomolybdic acid hydrate. Column chromatography was performed using silica gel (200–300 mesh) (Qingdao Haiyang Chemical Co., Ltd., Qingdao, Shandong, China). NMR spectra were obtained using BRUKER AVANCE III instrument (Bruker Co., Ltd., Switzerland). ^1^H-NMR spectra were recorded at 300 MHz or 400 MHz and are reported in parts per million (ppm) on the δ scale relative to tetramethylsilane (TMS) as an internal standard. ^13^C-NMR spectra were recorded at 75 MHz or 100 MHz and are reported in parts per million (ppm) on the δ scale relative to CDCl_3_ (δ 77.16) and DMSO-*d*_6_ (δ 39.52). Multiplicities are indicated as the following: s, singlet; d, doublet; t, triplet; q, quartet; m, multiplet; dd, doubled doublet; td, tripled doublet; br, broad. Coupling constants (*J* values) where noted are quoted in hertz. Mass spectra were obtained using Agilent 1260-6120 (ESI) instrument (Agilent Technologies Co., Ltd., Santa Clara, CA, USA). MW-promoted heating was obtained using MAS-II instrument manufactured (Sineo Microwave Chemistry Technology Co., Ltd., Shanghai, China). The melting point was uncorrected.

### 3.2. General Procedure for the Synthesis of HPAILs

To a well-stirred mixture of 12.2 g 1,3-propane sulfone (0.10 mol) in 30 mL toluene was added 8.9 mL pyridine (0.11 mol). The reaction mixture was stirred for 24 h at 50 °C under a nitrogen atmosphere resulting in a white precipitate (PyPS). After the completion of reaction, it was cooled to room temperature. PyPS was obtained after filtration, washed with diethyl ether and dried in a vacuum. Then, 18.1 g PyPS (0.09 mol) was added to an aqueous solution of 86.4 g H_3_PW_12_O_40_ (0.03 mol). After stirring at room temperature for 24 h, the solution was removed in a vacuum to give the HPAIL product [PyPS]_3_PW_12_O_40_ as a solid. Thus, [PyPS]_3_PMo_12_O_40_, [MIMPS]_3_PW_12_O_40_, [MIMPS]_3_PMo_12_O_40_, [TEAPS]_3_PW_12_O_40_ and [TEAPS]_3_PMo_12_O_40_ were prepared using according starting materials. Characterization data and copies of NMR spectra of all products can be found in the supplementary materials.

### 3.3. General Procedure for the Synthesis of 3,4-Dihydropyrimidin-2(1H)-ones

A mixture of alcohol (3 mmol), NaNO_3_ (0.255 g, 3 mmol), and [PyPS]_3_PW_12_O_40_ (0.42 g, 0.12 mmol) was added to a 30 mL glass pressure tube. After the pressure tube was closed, the reaction mixture was stirred at 80 °C under MW for 5–8 min (700 W). After the oxidation of alcohol (monitored by TLC), 1,3-dicarbonyl compound (3 mmol) and urea or thiourea (4.5 mmol) were added to the reaction mixture. The mixture was heated with stirring at 120 °C under MW for 5–10 min. On completion of the reaction (monitored by TLC), the mixture was diluted with hot ethyl acetate (20 mL) with stirring for 30 min. The insoluble catalyst was recovered by filtration as well as washing with ethyl acetate and subsequent ice water to remove traces of the previous organic reaction mixture and inorganic salt. The filtrate was evaporated and the residue was obtained in near pure form. Recrystallization or column chromatography could be used for further purification. Characterization data and copies of NMR spectra of all products can be found in the supplementary materials.

## 4. Conclusions

In conclusion, all the above results demonstrate that an efficient, eco-friendly and sustainable approach for the Biginelli reaction starting directly from alcohols using NaNO_3_-HPAIL system was achieved. The protocol provides compatibility with various functional groups and moderate to excellent yields to afford DHPMs in a two-step one-pot process. Moreover, the HPAILs are recyclable and reused for more than five cycles. The present work complements the well-known Biginelli reaction. Thus, the expansion as a valid and green alternative to other aldehyde-based MCRs is currently underway.

## Figures and Tables

**Table 1:** Optimization of the reaction conditions for benzyl alcohol, ethylacetoacetate and urea. ^a^

Entry	Catalyst (mol %)	T^1 b^/T^2 c^ (°C)	t^1 d^/t^2 e^ (min)	Yield ^f^ (%)
1 ^g^	[PyPS]_3_PW_12_O_40_ (3)	80	120	13
2	[PyPS]_3_PW_12_O_40_ (3)	80/80	5/30	51
3	[PyPS]_3_PW_12_O_40_ (3)	100/80	5/30	35
4	[PyPS]_3_PW_12_O_40_ (3)	80/100	5/10	78
5	[PyPS]_3_PW_12_O_40_ (3)	80/120	5/5	90
6	[PyPS]_3_PW_12_O_40_ (4)	80/120	5/5	92
7	[PyPS]_3_PW_12_O_40_ (5)	80/120	5/5	91
8	[PyPS]_3_PMo_12_O_40_ (4)	80/120	5/5	84
9	[MIMPS]_3_PW_12_O_40_ (4)	80/120	5/5	90
10	[MIMPS]_3_PMo_12_O_40_ (4)	80/120	5/5	80
11	[TEAPS]_3_PW_12_O_40_ (4)	80/120	5/5	78
12	[TEAPS]_3_PMo_12_O_40_ (4)	80/120	5/5	71
13	H_3_PW_12_O_40_ (4)	80/120	5/5	63

^a^ Reaction conditions: benzyl alcohol (3.0 mmol), ethylacetoacetate (3.0 mmol), urea (4.5 mmol), NaNO_3_ (3 mmol) and HPAIL catalyst under microwave (MW) (700 W) and solvent-free condition in the two-step one-pot procedure; ^b^ Temperatures for oxidation of alcohols; ^c^ Temperatures for cyclocondensations; ^d^ Reaction times for oxidation of alcohols; ^e^ Reaction times for cyclocondensations; ^f^ Isolated yields based on benzyl alcohol; ^g^ Reaction was conducted in the one-step one-pot procedure.

**Table 2:**
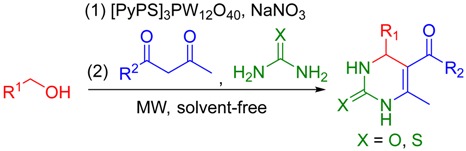
Scope of the Biginelli reaction starting directly from alcohols. ^a^

Entry	R^1^	R^2^	X	t^1 b^/t^2 c^ (min)	Yield ^d^ (%)
1	C_6_H_5_	OC_2_H_5_	O	5/5	92
2	4-CH_3_O-C_6_H_4_	OC_2_H_5_	O	5/5	94
3	4-CH_3_-C_6_H_4_	OC_2_H_5_	O	5/5	93
4	4-Cl-C_6_H_4_	OC_2_H_5_	O	5/5	90
5	4-NO_2_-C_6_H_4_	OC_2_H_5_	O	5/8	85
6	4-OH-C_6_H_4_	OC_2_H_5_	O	5/5	90
7	2-OH-C_6_H_4_	OC_2_H_5_	O	5/8	83
8	2-Furyl	OC_2_H_5_	O	5/8	84
9	n-C_6_H_13_	OC_2_H_5_	O	5/8	73
10	i-Pr	OC_2_H_5_	O	5/8	71
11	C_6_H_5_	OC_2_H_5_	S	5/10	90
12	4-CH_3_-C_6_H_4_	OC_2_H_5_	S	5/10	91
13	4-Cl-C_6_H_4_	OC_2_H_5_	S	5/10	90
14	4-NO_2_-C_6_H_4_	OC_2_H_5_	S	8/10	83
15	C_6_H_5_	OCH_3_	O	5/5	92
16	4-CH_3_O-C_6_H_4_	OCH_3_	O	5/5	95
17	4-CH_3_-C_6_H_4_	OCH_3_	O	5/5	91
18	4-Cl-C_6_H_4_	OCH_3_	O	5/5	89
19	4-NO_2_-C_6_H_4_	OCH_3_	O	8/8	80
20	C_6_H_5_	OCH_3_	S	5/10	88
21	4-CH_3_-C_6_H_4_	OCH_3_	S	5/10	90
22	4-NO_2_-C_6_H_4_	OCH_3_	S	5/10	78
23	C_6_H_5_	CH_3_	O	5/5	91
24	4-CH_3_-C_6_H_4_	CH_3_	O	5/5	92
25	4-NO_2_-C_6_H_4_	CH_3_	O	8/5	80
26	C_6_H_5_	CH_3_	S	5/5	88
27	4-CH_3_-C_6_H_4_	CH_3_	S	5/5	90
28	4-NO_2_-C_6_H_4_	CH_3_	S	8/5	77

^a^ Reaction conditions: alcohol (3.0 mmol), 1,3-dicarbonyl compound (3.0 mmol), urea or thiourea (4.5 mmol), NaNO_3_ (3 mmol) and [PyPS]_3_PW_12_O_40_ (4 mol %) at 80/120 °C under MW (700 W) and solvent-free condition in the two-step one-pot procedure; ^b^ Reaction times for oxidation of alcohols; ^c^ Reaction times for cyclocondensations; ^d^ Isolated yields based on alcohols.

## Supplementary Materials

Supplementary materials are available online.
