# Supplementary material for: An Efficient, Eco-friendly and Sustainable One-Pot Synthesis of 3,4-Dihydropyrimidin-2(1H)-ones Directly from Alcohols Catalyzed by Heteropolyanion-Based Ionic Liquids
Source: Molecules. 2017 Sep 11;22(9):1531. doi: 10.3390/molecules22091531 (PMC6151647; doi:10.3390/molecules22091531)
Supplement: Supplementary file 1 [file molecules-22-01531-s001.pdf]

Electronic Supplementary Information (ESI)

An efficient, Eco-friendly and Sustainable One-Pot Synthesis of 3,4-Dihydropyrimidin-2*(*1*H)*-ones Directly from Alcohols Catalyzed by Heteropolyanion-based Ionic Liquids

Renzhong Fu, Yang Yang *, Xudong Ma, Yu Sun, Jin Li, Hang Gao, Huaxing Hu, Xiaojun Zeng, Jun Yi *

Jiangsu Laboratory of Advanced Functional Material, School of Chemistry and Materials Engineering, Changshu Institute of Technology, Changshu 215500, PR China

Characterization data of all products

1-(3-Sulfopropyl)pyridine (PyPS)

White solid. Mp: 62.5-64.8 °C; IR (KBr) : 3443, 3159, 3103, 1572, 1459, 1184, 1010, 842, 780, 654, 601, 528 cm^{-1}; ^{1}H NMR (400 MHz, D_2O) δ 8.86 (d, J = 6.0 Hz, 2H), 8.53 (t, J = 7.8 Hz, 1H), 8.06 (t, J = 7.6 Hz, 2H), 4.75 (t, J = 7.2 Hz, 2H), 2.95 (t, J = 7.2 Hz, 2H), 2.47-2.40 (m, 2H); ^{13}C NMR (100 MHz, D_2O) δ 146.0, 144.5, 128.5, 60.0, 47.1, 26.2; HRMS Calcd for $C_8H_{12}NO_3S$ (M + H^+): 202.0532; Found: 202.0534.

1-Methyl-3-(3-sulfopropyl)imidazole (MIMPS)

White solid. Mp: 55.5-58.8 °C; IR (KBr) : 3433, 3025, 2944, 1631, 1487, 1328, 1197, 1099, 775, 687, 604, 522, 481 cm^{-1}; ^{1}H NMR (400 MHz, D_2O) δ 8.71 (s, 1H), 7.48 (s, 1H), 7.41 (s, 1H), 4.32 (t, J = 7.2 Hz, 2H), 3.86 (s, 3H), 2.88 (t, J = 7.2 Hz, 2H), 2.31-2.24 (m, 2H); ^{13}C NMR (100 MHz, D_2O) δ 136.2, 123.8, 122.2, 47.8, 47.3, 35.8, 25.1; HRMS Calcd for $C_7H_{13}N_2O_3S$ (M + H^+): 205.0641; Found: 205.0645.

Triethyl-(3-sulfopropyl)amine (TEAPS)

White solid. Mp: 48.5-49.8 °C; ^{1}H NMR (400 MHz, D_2O) δ 3.27 (q, J = 7.2 Hz, 8H), 2.93 (t, J = 7.2 Hz, 2H), 2.10-2.06 (m, 2H), 1.27-1.20 (m, 9H); ^{13}C NMR (100 MHz, D_2O) δ 52.7, 47.3, 46.7, 17.3, 8.3; HRMS Calcd for $C_9H_{22}NO_3S$ (M + H^+): 224.1315; Found: 224.1318.

1-(3-Sulfopropyl)pyridinium phosphotungstate ($[PyPS]_3PW_{12}O_{40}$)

White solid. Mp: 142.5-145.8 °C; IR (KBr) : 3409, 3131, 3068, 1633, 1488, 1220, 1182, 1079, 1042, 978, 896, 805, 681, 595, 521 cm^{-1}; ^{1}H NMR (400 MHz, D_2O) δ 8.87 (brs, 2H), 8.59 (brs, 1H), 8.11 (brs, 2H), 4.78 (brs, 2H), 2.97 (brs, 2H), 2.46 (brs, 2H); ^{13}C NMR (100 MHz, D_2O) δ 146.3, 144.4, 128.6, 60.2, 47.2, 26.2; HRMS Calcd for $C_8H_{12}NO_3S$ (M + H^+): 202.0532; Found: 202.0535.

1-(3-Sulfopropyl)pyridinium hosphomolybdate ($[PyPS]_3PMo_{12}O_{40}$)

Green solid. Mp: 142.5-145.8 °C; IR (KBr) : 3421, 3066, 1633, 1488, 1181, 1062, 1044, 957, 880, 795, 680, 609, 505 cm^{-1}; ^{1}H NMR (400 MHz, D_2O) δ 8.87 (brs, 2H), 8.67 (t,

J = 7.2 Hz, 1H), 8.20 (t, J = 7.2 Hz, 2H), 4.82 (t, J = 7.2 Hz, 2H), 2.98 (t, J = 7.2 Hz, 2H), 2.51-2.43 (m, 2H); ^{13}C NMR (100 MHz, D_2O) δ 146.4, 144.3, 129.0, 60.3, 47.3, 26.5; HRMS Calcd for $C_8H_{12}NO_3S$ (M + H^+): 202.0532; Found: 202.0536.

1-Methyl-3-(3-sulfopropyl)imidazolium phosphotungstate ($[MIMPS]_3PW_{12}O_{40}$)

White solid. Mp: 119.5-120.8 °C; IR (KBr) : 3417, 3147, 1636, 1571, 1459, 1418, 1173, 1080, 980, 897, 800, 516 cm^{-1}; ^{1}H NMR (400 MHz, D_2O) δ 8.68 (s, 1H), 7.55 (s, 1H), 7.51 (s, 1H), 4.42 (t, J = 7.2 Hz, 2H), 4.02 (s, 3H), 2.91 (t, J = 7.2 Hz, 2H), 2.36-2.28 (m, 2H); ^{13}C NMR (100 MHz, D_2O) δ 135.9, 124.2, 122.6, 48.1, 47.4, 36.1, 25.3; HRMS Calcd for $C_7H_{13}N_2O_3S$ (M + H^+): 205.0641; Found: 205.0645.

1-Methyl-3-(3-sulfopropyl)imidazolium hosphomolybdate ($[MIMPS]_3PMo_{12}O_{40}$)

Green solid. Mp: 105.5-108.8 °C; IR (KBr) : 3422, 3149, 3100, 1630, 1569, 1454, 1405, 1217, 1171, 1059, 963, 882, 796, 610, 504 cm^{-1}; ^{1}H NMR (400 MHz, D_2O) δ 8.63 (s, 1H), 7.48 (s, 1H), 7.42 (s, 1H), 4.35 (brs, 2H), 3.92 (s, 3H), 2.86 (brs, 2H), 2.26 (brs, 2H); ^{13}C NMR (100 MHz, D_2O) δ 136.3, 124.3, 122.6, 48.2, 47.4, 36.1, 25.3; HRMS Calcd for $C_7H_{13}N_2O_3S$ (M + H^+): 205.0641; Found: 205.0643.

Triethyl-(3-sulfopropyl)aminium phosphotungstate ($[TEAPS]_3PW_{12}O_{40}$)

White solid. Mp: 185.5-187.8 °C; IR (KBr) : 3440, 1485, 1460, 1400, 1230, 1170, 1080, 978, 893, 810 cm^{-1}; ^{1}H NMR (400 MHz, D_2O) δ 3.29-3.28 (m, 8H), 2.98-2.93 (m, 2H), 2.17-2.01 (m, 2H), 1.28-1.25 (m, 9H); ^{13}C NMR (100 MHz, D_2O) δ 60.2, 52.9, 48.0, 24.3, 6.7; HRMS Calcd for $C_9H_{22}NO_3S$ (M + H^+): 224.1315; Found: 224.1318.

Triethyl-(3-sulfopropyl)aminium hosphomolybdate ($[TEAPS]_3PMo_{12}O_{40}$)

Green solid. Mp: 165.5-167.8 °C; ^{1}H NMR (400 MHz, D_2O) δ 3.37-3.21 (m, 8H), 2.97-2.87 (m, 2H), 2.16-2.06 (m, 2H), 1.28-1.25 (m, 9H); ^{13}C NMR (100 MHz, D_2O) δ 60.2, 52.9, 48.0, 24.3, 6.7; HRMS Calcd for $C_9H_{22}NO_3S$ (M + H^+): 224.1315; Found: 224.1318.

5-Ethoxycarbonyl-6-methyl-4-phenyl-3,4-dihydropyrimidin-*2(1H)*-one

White solid. Mp: 201.1-203.6 °C; IR (KBr): 3244, 3109, 2933, 1710, 1650, 1222, 1089 cm^{-1}; ^{1}H NMR (300 MHz, DMSO-d_6) δ 9.20 (s, 1H), 7.74 (s, 1H), 7.35-7.30 (m, 2H),

7.25-7.21 (m, 3H), 5.15 (d, J = 3.0 Hz, 1H), 3.98 (q, J = 7.0 Hz, 2H), 2.25 (s, 3H), 1.09 (t, J = 7.0 Hz, 3H); ^{13}C NMR (75 MHz, DMSO-d_6) δ 165.4, 152.2, 148.4, 144.9, 128.4, 127.3, 126.3, 99.3, 59.2, 54.0, 17.8, 14.1; HRMS Calcd for $C_{14}H_{17}N_2O_3$ (M + H$^+$): 261.1234; Found: 261.1238.

5-Ethoxycarbonyl-6-methyl-4-(4-methoxyphenyl)-3,4-dihydropyrimidin-*2(1H)*-one

White solid. Mp: 203.1-205.7 °C; IR (KBr): 3243, 3111, 2930, 1709, 1649, 1223, 1089 cm^{-1}; ^{1}H NMR (300 MHz, DMSO-d_6) δ 9.16 (s, 1H), 7.67 (s, 1H), 7.15 (d, J = 8.7 Hz, 2H), 6.87 (d, J = 8.7 Hz, 2H), 5.10 (d, J = 3.0 Hz, 1H), 3.97 (q, J = 7.0 Hz, 2H), 3.71 (s, 3H), 2.24 (s, 3H), 1.10 (t, J = 7.0 Hz, 3H); ^{13}C NMR (75 MHz, DMSO-d_6) δ 165.4, 158.5, 152.2, 148.1, 137.1, 127.5, 113.7, 99.6, 59.2, 55.1, 53.4, 17.8, 14.2; HRMS Calcd for $C_{15}H_{19}N_2O_4$ (M + H$^+$): 291.1339; Found: 291.1337.

5-Ethoxycarbonyl-6-methyl-4-(4-methylphenyl)-3,4-dihydropyrimidin-*2(1H)*-one

Yellow solid. Mp: 216.1-217.1 °C; IR (KBr): 3231, 3094, 2916, 1701, 1649, 1456, 1285, 1219, 1082, 777 cm^{-1}; ^{1}H NMR (300 MHz, DMSO-d_6) δ 9.16 (s, 1H), 7.69 (s, 1H), 7.12 (s, 4H), 5.10 (d, J = 2.1 Hz, 1H), 3.98 (q, J = 7.2 Hz, 2H), 2.26 (s, 3H), 2.24 (s, 3H), 1.10 (t, J = 7.2 Hz, 3H); ^{13}C NMR (75 MHz, DMSO-d_6) δ 165.4, 152.2, 148.2, 142.0, 136.4, 128.9, 126.2, 99.4, 59.2, 53.6, 20.7, 17.8, 14.1; HRMS Calcd for $C_{15}H_{19}N_2O_3$ (M + H$^+$): 275.1390; Found: 275.1394.

5-Ethoxycarbonyl-6-methyl-4-(4-chlorophenyl)-3,4-dihydropyrimidin-*2(1H)*-one

Yellow solid. Mp: 214.8-216.6 °C; IR (KBr): 3245, 3119, 2972, 1650, 1487, 1471, 1287, 1220, 1084, 778, 484 cm^{-1}; ^{1}H NMR (300 MHz, DMSO-d_6) δ 9.32 (s, 1H), 9.11 (s, 1H), 7.39 (d, J = 8.4 Hz, 2H), 7.25 (d, J = 8.4 Hz, 2H), 5.14 (d, J = 3.3 Hz, 1H), 3.98 (q, J = 7.2 Hz, 2H), 2.25 (s, 3H), 1.09 (t, J = 7.2 Hz, 3H); ^{13}C NMR (75 MHz, DMSO-d_6) δ 165.2, 152.0, 148.7, 143.8, 131.8, 128.4, 128.2, 98.8, 59.3, 53.4, 17.8, 14.1; HRMS Calcd for $C_{14}H_{16}ClN_2O_3$ (M + H$^+$): 295.0844; Found: 295.0841.

5-Ethoxycarbonyl-6-methyl-4-(4-nitrophenyl)-3,4-dihydropyrimidin-*2(1H)*-one

Yellow solid. Mp: 201.1-202.7 °C; IR (KBr): 3230, 3109, 2977, 1701, 1641, 1591, 1520 cm^{-1}; ^{1}H NMR (300 MHz, DMSO-*d*$_6$) δ 9.37 (s, 1H), 8.22 (d, *J* = 8.7 Hz, 2H), 7.90 (s, 1H), 7.50 (d, *J* = 8.7 Hz, 2H), 5.27 (d, *J* = 3.0 Hz, 1H), 3.98 (q, *J* = 6.9 Hz, 2H), 2.26 (s, 3H), 1.09 (t, *J* = 6.9 Hz, 3H); ^{13}C NMR (75 MHz, DMSO-*d*$_6$) δ 165.1, 152.0, 151.8, 149.4, 146.7, 127.7, 123.9, 98.2, 59.4, 53.7, 17.9, 17.8, 14.1; HRMS Calcd for $C_{14}H_{16}N_3O_5$ (M + H^+): 306.1084; Found: 306.1086.

5-Ethoxycarbonyl-6-methyl-4-(4-hydroxyphenyl)-3,4-dihydropyrimidin-*2(1H)*-one

Yellow solid. Mp: 198.4-200.1 °C; IR (KBr): 3520, 3230, 3150, 1705, 1690 cm^{-1}; ^{1}H NMR (300 MHz, DMSO-*d*$_6$) δ 9.25 (s, 1H), 7.78 (s, 1H), 7.62 (s, 1H), 7.02 (d, *J* = 8.4 Hz, 2H), 6.68 (d, *J* = 8.4 Hz, 2H), 5.04 (d, *J* = 2.7 Hz, 1H), 3.97 (q, *J* = 7.2 Hz, 2H), 2.23 (s, 3H), 1.09 (t, *J* = 7.2 Hz, 3H); ^{13}C NMR (75 MHz, DMSO-*d*$_6$) δ 165.4, 156.5, 152.2, 147.8, 135.5, 127.4, 115.0, 99.7, 59.1, 53.4, 17.8, 14.1; HRMS Calcd for $C_{14}H_{17}N_2O_4$ (M + H^+): 277.1183; Found: 277.1186.

5-Ethoxycarbonyl-6-methyl-4-(2-hydroxyphenyl)-3,4-dihydropyrimidin-*2(1H)*-one

White solid. Mp: 223.1-224.5 °C; IR (KBr): 3354, 3268, 1683, 1597 cm^{-1}; ^{1}H NMR (300 MHz, DMSO-*d*$_6$) δ 9.57 (brs, 1H), 9.09 (s, 1H), 7.10 (s, 1H), 7.05 (td, *J* = 7.5, 1.5 Hz, 1H), 6.97 (dd, *J* = 7.5, 1.5 Hz, 1H), 6.79 (dd, *J* = 7.5, 0.6 Hz, 1H), 6.71 (td, *J* = 7.5, 0.6 Hz, 1H), 5.46 (d, *J* = 3.0 Hz, 1H), 3.92 (q, *J* = 7.2 Hz, 2H), 2.27 (s, 3H), 1.04 (t, *J* = 7.2 Hz, 3H); ^{13}C NMR (75 MHz, DMSO-*d*$_6$) δ 165.5, 154.7, 152.4, 148.6, 129.9, 128.3, 127.3, 118.8, 115.4, 97.8, 59.1, 49.2, 17.8, 14.1; HRMS Calcd for $C_{14}H_{17}N_2O_4$ (M + H^+): 277.1183; Found: 277.1186.

5-Ethoxycarbonyl-6-methyl-4-(furan-2-yl)-3,4-dihydropyrimidin-*2(1H)*-one

Yellow solid. Mp: 207.2-208.9 °C; IR (KBr): 3364, 3221, 3120, 2970, 1699, 1638, 1457, 1372, 1299, 1229, 1092, 1014, 793, 742, 449 cm^{-1}; ^{1}H NMR (300 MHz, DMSO-*d*$_6$) δ 9.24 (s, 1H), 7.76 (s, 1H), 7.55 (dd, *J* = 1.8, 0.9 Hz, 1H), 6.35 (dd, *J* = 3.0, 1.8 Hz, 1H), 6.09

(d, *J* = 3.3 Hz, 1H), 5.21 (d, *J* = 3.6 Hz, 1H), 4.02 (qd, *J* = 7.2, 0.9 Hz, 2H), 2.23 (s, 3H), 1.13 (t, *J* = 7.2 Hz, 3H); ^{13}C NMR (75 MHz, DMSO-*d6*) *δ* 165.1, 156.0, 152.5, 149.4, 142.2, 110.4, 105.3, 96.8 59.3, 47.8, 17.8, 14.2; HRMS Calcd for $C_{12}H_{15}N_2O_4$ (M + H$^+$): 251.1026; Found: 251.1024.

5-Ethoxycarbonyl-6-methyl-4-(n-hexyl)-3,4-dihydropyrimidin-*2(1H)*-one

Yellow solid. Mp: 229.8-231.2°C; IR (KBr): 3248, 3121, 2957, 2936, 2855, 1724, 1703, 1647, 1383, 1288, 1223, 1086, 779 cm^{-1}; ^{1}H NMR (300 MHz, DMSO-*d6*) *δ* 8.92 (s, 1H), 7.32 (s, 1H), 4.11-3.99 (m, 3H), 2.15 (m, 3H), 1.38 (m, 10H), 1.18 (t, *J* = 6.9 Hz, 3H), 0.84 (t, *J* = 6.9 Hz, 3H); ^{13}C NMR (75 MHz, DMSO-*d6*) *δ* 165.5, 152.8, 148.3, 99.5, 59.1, 50.1, 36.7, 31.3, 28.5, 23.7, 22.1, 17.7, 14.2, 14.0; HRMS Calcd for $C_{14}H_{25}N_2O_3$ (M + H$^+$): 269.1860; Found: 269.1863.

5-Ethoxycarbonyl-6-methyl-4-isopropyl-3,4-dihydropyrimidin-*2(1H)*-one

Yellow solid. Mp: 193.4-194.8 °C; IR (KBr): 3234, 2985, 1710, 1642 cm^{-1}; ^{1}H NMR (300 MHz, DMSO-*d6*) *δ* 8.90 (s, 1H), 7.30 (s, 1H), 4.11-4.00 (m, 2H), 3.94 (t, *J* = 3.6 Hz, 1H), 2.17 (s, 3H), 1.70-1.62 (m, 1H), 1.18 (t, *J* = 7.1 Hz, 1H), 0.81 (d, *J* = 6.9 Hz, 3H), 0.74 (d, *J* = 6.9 Hz, 3H); ^{13}C NMR (75 MHz, DMSO-*d6*) *δ* 165.8, 153.2, 148.5, 98.1, 59.0, 55.5, 34.6, 18.5, 17.7, 16.0, 14.2; HRMS Calcd for $C_{11}H_{19}N_2O_3$ (M + H$^+$): 227.1390; Found: 227.1387.

5-Ethoxycarbonyl-6-methyl-4-phenyl-3,4-dihydropyrimidin-*2(1H)*-thione

Yellow solid. Mp: 200.4-202.5 °C; IR (KBr): 3322, 34663176, 3111, 1670, 1575, 1470, 1277, 1197, 1105, 696 cm^{-1}; ^{1}H NMR (300 MHz, DMSO-*d6*) *δ* 10.34 (s, 1H), 9.65 (s, 1H), 7.37-7.21 (m, 5H), 5.18 (d, *J* = 3.6 Hz, 1H), 4.01 (q, *J* = 6.9 Hz, 2H), 2.29 (s, 3H), 1.10 (t, *J* = 6.9 Hz, 3H); ^{13}C NMR (75 MHz, DMSO-*d6*) *δ* 174.2, 165.1, 145.0, 143.5, 128.6, 127.7, 126.4, 100.7, 59.6, 54.1, 17.2, 14.0; HRMS Calcd for $C_{14}H_{17}N_2O_2S$ (M + H$^+$): 277.1005; Found: 277.1006.

5-Ethoxycarbonyl-6-methyl-4-(4-methylphenyl)-3,4-dihydropyrimidin-*2(1H)*-thione

Yellow solid. Mp: 192.3-194.1 °C; IR (KBr): 3255, 1659, 1562 cm^{-1}; ^1H NMR (300 MHz, DMSO-d_6) δ 10.30 (s, 1H), 9.61 (d, J = 1.8 Hz, 1H), 7.16 (m, 4H), 5.13 (d, J = 3.6 Hz, 1H), 4.00 (q, J = 7.2 Hz, 2H), 2.28 (s, 3H), 2.26 (s, 3H), 1.10 (t, J = 7.2 Hz, 3H); ^{13}C NMR (75 MHz, DMSO-d_6) δ 174.2, 165.2, 144.9, 140.6, 136.9, 129.1, 126.3, 100.8, 59.6, 53.8, 20.7, 17.2, 14.1; HRMS Calcd for $C_{15}H_{19}N_2O_2S$ (M + H^+): 291.1162; Found: 291.1160.

5-Ethoxycarbonyl-6-methyl-4-(4-chlorophenyl)-3,4-dihydropyrimidin-*2(1H)*-thione

Colorless solid. Mp: 168.1-169.6 °C; IR (KBr): 3331, 3167, 3105, 2978, 1668, 1570, 1197, 747 cm^{-1}; ^1H NMR (300 MHz, DMSO-d_6) δ 10.39 (s, 1H), 9.67 (s, 1H), 7.44-7.40 (m, 2H), 7.25-7.20 (m, 2H), 5.17 (d, J = 3.6 Hz, 1H), 4.00 (q, J = 7.0 Hz, 2H), 2.29 (s, 3H), 1.09 (t, J = 7.0 Hz, 3H); ^{13}C NMR (75 MHz, DMSO-d_6) δ 174.3, 165.0, 145.4, 142.4, 132.3, 128.6, 128.3, 100.3, 59.7, 53.5, 17.2, 14.0; HRMS Calcd for $C_{14}H_{16}ClN_2O_2S$ (M + H^+): 311.0616; Found: 311.0619.

5-Ethoxycarbony-6-methyl-4-(4-nitrophenyl)-3,4-dihydro-pyrimidin-2-thione

Yellow solid. Mp: 200.0-202.1 °C; IR (KBr): 3324, 3173, 2918, 1679, 1655, 1576, 1522, 1466, 1350, 1201, 1176, 1123 cm^{-1}; ^1H NMR (300 MHz, DMSO-d_6) δ 10.50 (s, 1H), 9.77 (s, 1H), 8.24 (d, J = 8.7 Hz, 2H), 7.49 (d, J = 8.7 Hz, 2H), 5.30 (d, J = 3.6 Hz, 1H), 4.01 (q, J = 6.9 Hz, 2H), 2.31 (s, 3H), 1.10 (t, J = 6.9 Hz, 3H); ^{13}C NMR (75 MHz, DMSO-d_6) δ 174.4, 164.8, 150.4, 147.0, 146.0, 127.8, 124.0, 99.7, 59.8, 53.7, 17.3, 14.0; HRMS Calcd for $C_{14}H_{16}N_3O_4S$ (M + H^+): 322.0856; Found: 322.0854.

5-Methoxycarbonyl-6-methyl-4-phenyl-3,4-dihydropyrimidin-*2(1H)*-one

Yellow solid. Mp: 211.3-213.7 °C; IR (KBr): 3238, 3117, 2933, 1719, 1645, 1222, 1092 cm^{-1}; ^1H NMR (300 MHz, DMSO-d_6) δ 9.23 (s, 1H), 7.77 (s, 1H), 7.35-7.30 (m, 2H), 7.26-7.21 (m, 3H), 5.15 (d, J = 3.3 Hz, 1H), 3.53 (s, 3H), 2.26 (s, 3H); ^{13}C NMR (75 MHz, DMSO-d_6) δ 165.9, 152.2, 148.7, 144.7, 128.5, 127.4, 126.2, 99.1, 53.9, 50.9, 17.9; HRMS Calcd for $C_{13}H_{15}N_2O_3$ (M + H^+): 247.1077; Found: 247.1079.

5-Methoxycarbonyl-6-methyl-4-(4-methoxyphenyl)-3,4-dihydropyrimidin-*2(1H)*-o

ne

Yellow solid. Mp: 192.1-194.2 °C; IR (KBr): 3232, 3104, 3003, 2950, 2837, 1698, 1645, 1609, 1512 cm^{-1}; ^{1}H NMR (300 MHz, DMSO-*d6*) δ 9.19 (s, 1H), 7.69 (s, 1H), 7.14 (d, J = 8.4 Hz, 2H), 6.87 (d, J = 8.4 Hz, 2H), 5.09 (d, J = 3.0 Hz, 1H), 3.72 (s, 3H), 3.52 (s, 3H), 2.25 (s, 3H); ^{13}C NMR (75 MHz, DMSO-*d6*) δ 165.9, 158.5, 152.2, 148.4, 136.9, 127.3, 113.8, 99.3, 55.1, 53.2, 50.8, 17.8; HRMS Calcd for $C_{14}H_{17}N_2O_4$ (M + H^+): 277.1183; Found: 277.1186.

5-Methoxycarbonyl-6-methyl-4-(4-methylphenyl)-3,4-dihydropyrimidin-*2(1H)*-one

Yellow solid. Mp: 204.0-206.4 °C; IR (KBr): 3175, 1691, 1634 cm^{-1}; ^{1}H NMR (300 MHz, DMSO-*d6*) δ 9.19 (s, 1H), 7.71 (s, 1H), 7.11 (s, 4H), 5.11 (d, J = 3.3 Hz, 1H), 3.52 (s, 3H), 2.26 (s, 3H), 2.24 (s, 3H); ^{13}C NMR (75 MHz, DMSO-*d6*) δ 165.9, 152.2, 148.5, 141.8, 136.4, 129.0, 126.1, 99.2, 53.5, 50.8, 20.7, 17.8; HRMS Calcd for $C_{14}H_{17}N_2O_3$ (M + H^+): 261.1234; Found: 261.1236.

5-Methoxycarbonyl-6-methyl-4-(4-chlorophenyl)-3,4-dihydropyrimidin-*2(1H)*-one

Yellow solid. Mp: 204.3-207.1 °C; IR (KBr): 3238, 3114, 2950, 2875, 1702, 1645 cm^{-1}; ^{1}H NMR (300 MHz, DMSO-*d6*) δ 9.28 (s, 1H), 7.80 (s, 1H), 7.39 (d, J = 8.4 Hz, 2H), 7.24 (d, J = 8.7 Hz, 2H), 5.14 (d, J = 3.3 Hz, 1H), 3.53 (s, 3H), 2.25 (s, 3H); ^{13}C NMR (75 MHz, DMSO-*d6*) δ 165.7, 152.0, 149.0, 143.6, 131.9, 128.5, 128.1, 98.6, 53.3, 50.9, 17.9; HRMS Calcd for $C_{13}H_{14}ClN_2O_3$ (M + H^+): 281.0687; Found: 281.0689.

5-Methoxycarbonyl-6-methyl-4-(4-nitrophenyl)-3,4-dihydropyrimidin-*2(1H)*-one

Yellow solid. Mp: 235.5-237.3 °C; IR (KBr): 3366, 3233, 3113, 2951, 1703, 1643, 1598, 1520 cm^{-1}; ^{1}H NMR (300 MHz, DMSO-*d6*) δ 9.38 (s, 1H), 8.21 (d, J = 8.9 Hz, 2H), 7.91 (s, 1H), 7.50 (d, J = 8.9 Hz, 2H), 5.27 (d, J = 3.3 Hz, 1H), 3.53 (s, 3H), 2.27 (s, 3H); ^{13}C NMR (75 MHz, DMSO-*d6*) δ 165.6, 151.8, 151.7, 149.6, 146.7, 127.6, 123.9, 98.0, 53.5, 50.9, 17.9; HRMS Calcd for $C_{13}H_{14}N_3O_5$ (M + H^+): 292.0928; Found: 292.0925.

5-Methoxycarbonyl-6-methyl-4-phenyl-3,4-dihydropyrimidin-*2(1H)*-thione

Yellow solid. Mp: 202.1-204.5 °C; IR (KBr): 3340, 1662, 1600, 1566 cm^{-1}; ^{1}H NMR (300 MHz, DMSO-d_6) δ 10.37 (s, 1H), 9.68 (d, J = 1.8 Hz, 1H), 7.38-7.32 (m, 2H), 7.30-7.26 (m, 1H), 7.25-7.21 (m, 2H), 5.18 (d, J = 3.6 Hz, 1H), 3.56 (s, 3H), 2.30 (s, 3H); ^{13}C NMR (75 MHz, DMSO-d_6) δ 174.3, 165.7, 145.3, 143.3, 128.7, 127.7, 128.3, 100.5, 53.9, 51.1, 17.3; HRMS Calcd for $C_{13}H_{15}N_2O_2S$ (M + H^+): 263.0849; Found: 263.0846.

5-Methoxycarbonyl-6-methyl-4-(4-methylphenyl)-3,4-dihydropyrimidin-*2(1H)*-thione

Yellow solid. Mp: 155.3-156.9 °C; IR (KBr): 3334, 3200, 2970, 1669, 1570, 1470, 1183 cm^{-1}; ^{1}H NMR (300 MHz, DMSO-d_6) δ 10.34 (s, 1H), 9.65 (d, J = 1.8 Hz, 1H), 7.16-7.08 (m, 4H), 5.13 (d, J = 3.6 Hz, 1H), 3.54 (s, 3H), 2.29 (s, 3H), 2.26 (s, 3H); ^{13}C NMR (75 MHz, DMSO-d_6) δ 174.2, 165.7, 145.2, 140.4, 136.9, 129.1, 126.2, 100.5, 53.6, 51.1, 20.7, 17.2; HRMS Calcd for $C_{14}H_{17}N_2O_2S$ (M + H^+): 277.1005; Found: 277.1001.

5-Methoxycarbonyl-6-methyl-4-(4-nitrophenyl)-3,4-dihydropyrimidin-*2(1H)*-thione

Yellow solid. Mp: 122.2-124.7 °C; IR (KBr): 3178, 3105, 2997, 2952, 1693, 1567, 1516 cm^{-1}; ^{1}H NMR (300 MHz, DMSO-d_6) δ 10.52 (s, 1H), 9.78 (s, 1H), 8.23 (d, J = 8.7 Hz, 2H), 7.49 (d, J = 8.7 Hz, 2H), 5.31 (d, J = 3.6 Hz, 1H), 3.56 (s, 3H), 2.31 (s, 3H); ^{13}C NMR (75 MHz, DMSO-d_6) δ 174.5, 165.4, 150.2, 147.0, 146.2, 127.8, 124.1, 99.5, 53.6, 51.3, 17.4; HRMS Calcd for $C_{13}H_{14}N_3O_4S$ (M + H^+): 308.0700; Found: 308.0702.

5-Acetyl-6-methyl-4-phenyl-3,4-dihydropyrimidin-*2(1H)*-one

Yellow solid. Mp: 238.5-240.9 °C; IR (KBr): 3287, 3241, 2914, 1706, 603, 1466, 1248, 764 cm^{-1}; ^{1}H NMR (300 MHz, DMSO-d_6) δ 9.21 (s, 1H), 7.86 (s, 1H), 7.35-7.30 (m, 2H), 7.26-7.22 (m, 3H), 5.26 (d, J = 3.3 Hz, 1H), 3.54 (s, 3H), 2.29 (s, 3H), 2.10 (s, 3H); ^{13}C NMR (75 MHz, DMSO-d_6) δ 194.3, 152.2, 148.2, 144.3, 128.6, 127.4, 126.5, 109.6, 53.9, 30.4, 19.0; HRMS Calcd for $C_{13}H_{15}N_2O_2$ (M + H^+): 231.1128; Found: 231.1124.

5-Acetyl-6-methyl-4-(4-methylphenyl)-3,4-dihydropyrimidin-*2(1H)*-one

Yellow solid. Mp: 204.2-206.5 °C; IR (KBr): 3288, 3120, 2920, 1699, 1616, 1236,

1139, 765, 561 cm^{-1}; ^{1}H NMR (300 MHz, DMSO-*d*$_6$) δ 9.16 (s, 1H), 7.78 (s, 1H), 7.13 (s, 4H), 5.22 (d, *J* = 3.0 Hz, 1H), 2.28 (s, 3H), 2.26 (s, 3H), 2.08 (s, 3H); ^{13}C NMR (75 MHz, DMSO-*d*$_6$) δ 194.4, 152.2, 148.0, 141.3, 136.5, 129.1, 126.4, 109.6, 53.6, 30.3, 20.7, 18.9; HRMS Calcd for $C_{14}H_{17}N_2O_2$ (M + H$^+$): 245.1285; Found: 245.1288.

5-Acetyl-6-methyl-4-(4-nitrophenyl)-3,4-dihydropyrimidin-*2(1H)*-one

Yellow solid. Mp: 263.3-264.8 °C; IR (KBr): 3339, 3254, 3138, 1709, 1672, 1607, 1514, 1445, 1348, 1236, 1103, 762 cm^{-1}; ^{1}H NMR (300 MHz, DMSO-*d*$_6$) δ 9.34 (s, 1H), 9.20 (d, *J* = 8.7 Hz, 2H), 7.99 (s, 1H), 7.50 (d, *J* = 8.7 Hz, 2H), 5.38 (d, *J* = 3.6 Hz, 1H), 2.31 (s, 3H), 2.18 (s, 3H); ^{13}C NMR (75 MHz, DMSO-*d*$_6$) δ 193.9, 152.0, 151.6, 149.1, 146.7, 127.7, 123.8, 109.5, 53.2, 30.7, 19.1; HRMS Calcd for $C_{13}H_{14}N_3O_3$ (M + H$^+$): 276.0979; Found: 276.0981.

5-Acetyl-6-methyl-4-phenyl-3,4-dihydropyrimidin-*2(1H)*-thione

Yellow solid. Mp: 228.1-230.3 °C; IR (KBr): 3323, 3180, 2984, 1669, 1578, 1463, 1272, 1201, 758 cm^{-1}; ^{1}H NMR (300 MHz, DMSO-*d*$_6$) δ 10.28 (s, 1H), 9.76 (s, 1H), 7.38-7.22 (m, 5H), 5.29 (d, *J* = 3.9 Hz, 1H), 2.33 (s, 3H), 2.16 (s, 3H); ^{13}C NMR (75 MHz, DMSO-*d*$_6$) δ 194.8, 174.1, 144.6, 142.9, 128.6, 127.7, 126.6, 110.5, 53.8, 30.4, 18.3; HRMS Calcd for $C_{13}H_{15}N_2OS$ (M + H$^+$): 247.0900; Found: 247.0901.

5-Acetyl-6-methyl-4-(4-methylphenyl)-3,4-dihydropyrimidin-*2(1H)*-thione

Yellow solid. Mp: 215.7-217.3 °C; ^{1}H NMR (300 MHz, DMSO-*d*$_6$) δ 10.25 (s, 1H), 9.72 (s, 1H), 7.16-7.09 (m, 4H), 5.25 (d, *J* = 3.9 Hz, 1H), 2.32 (s, 3H), 2.26 (s, 3H), 2.13 (s, 3H); ^{13}C NMR (75 MHz, DMSO-*d*$_6$) δ 194.8, 174.0, 144.4, 140.0, 137.0, 129.2, 126.5, 110.4, 53.6, 30.3, 20.7, 18.2; HRMS Calcd for $C_{14}H_{17}N_2OS$ (M + H$^+$): 261.1056; Found: 261.1059.

5-Acetyl-6-methyl-4-(4-nitrophenyl)-3,4-dihydropyrimidin-*2(1H)*-thione

Yellow solid. Mp: 207.3-209.5 °C; ^{1}H NMR (300 MHz, DMSO-*d*$_6$) δ 10.46 (s, 1H), 9.89 (d, *J* = 2.4 Hz, 1H), 8.22 (d, *J* = 8.7 Hz, 2H), 7.48 (d, *J* = 8.7 Hz, 2H), 5.41 (d, *J* = 3.9 Hz, 1H), 2.36 (s, 3H), 2.23 (s, 3H); ^{13}C NMR (75 MHz, DMSO-*d*$_6$) δ 194.6, 174.6, 150.0,

146.9, 145.6, 127.8, 124.0, 110.3, 53.1, 30.8, 18.5; HRMS Calcd for $C_{13}H_{14}N_3O_3S$ (M + H^+): 292.0750; Found: 292.0753.

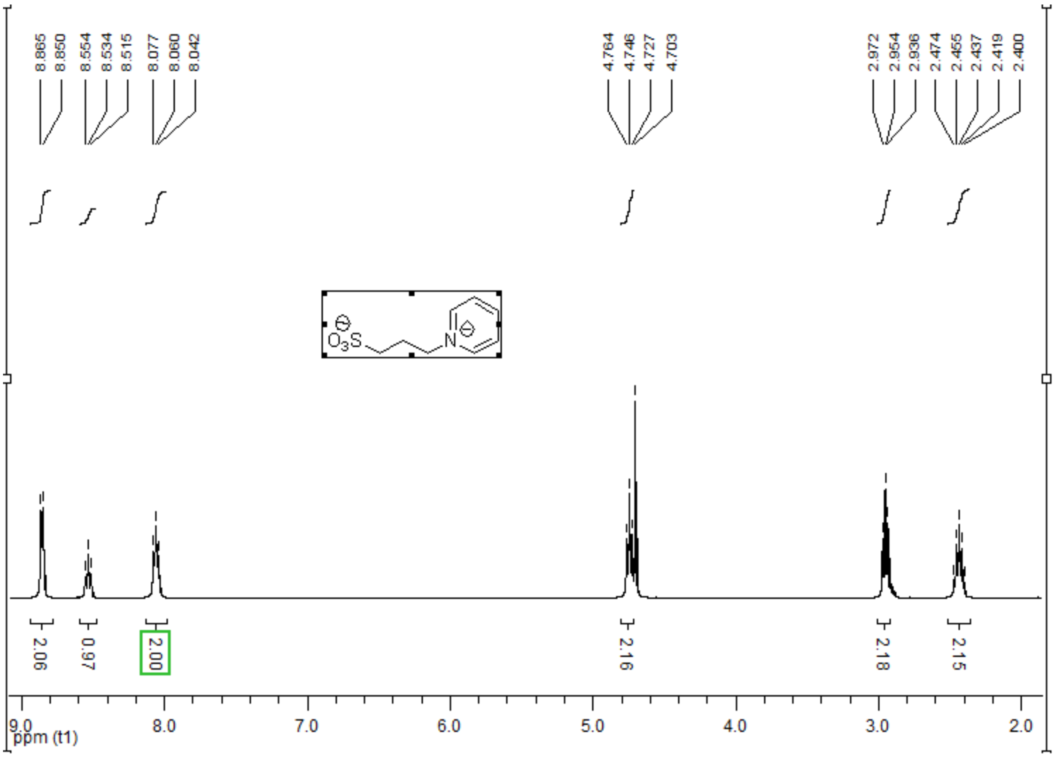
8.865
8.850
8.554
8.534
8.515
8.077
8.060
8.042
4.764
4.746
4.727
4.703
2.972
2.954
2.936
2.474
2.455
2.437
2.419
2.400
2.06
0.97
2.00
2.16
2.18
2.15
9.0
8.0
7.0
6.0
5.0
4.0
3.0
2.0
ppm (t1)

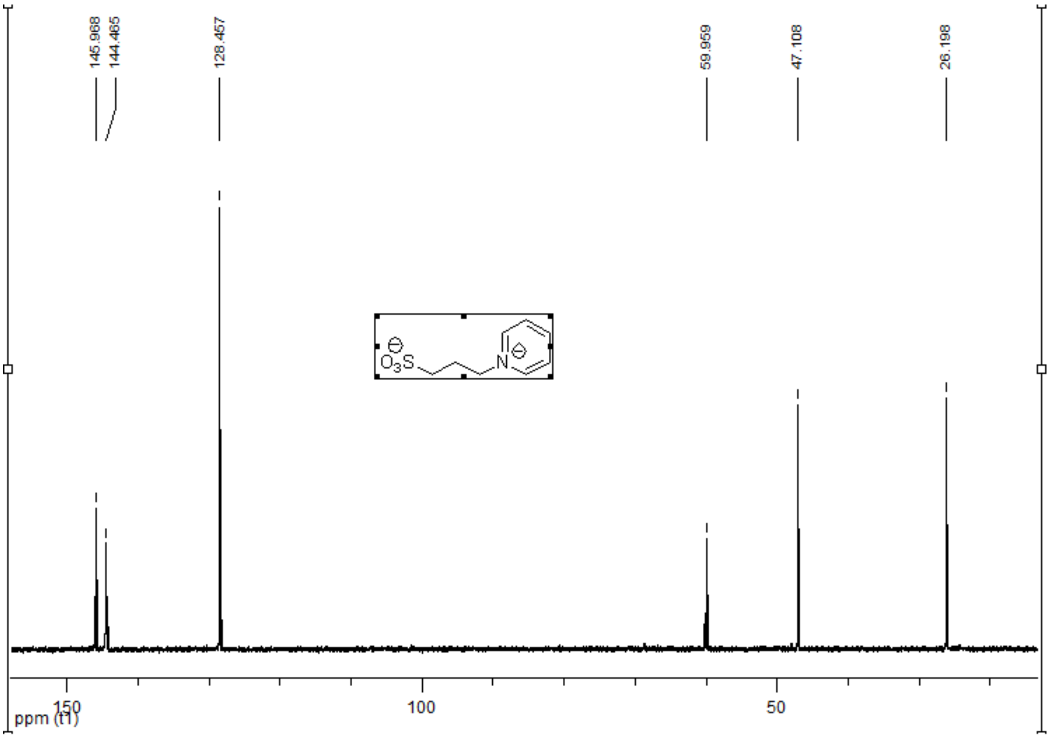
145.968
144.465
128.457
59.959
47.108
26.198
150
100
50
ppm (t1)

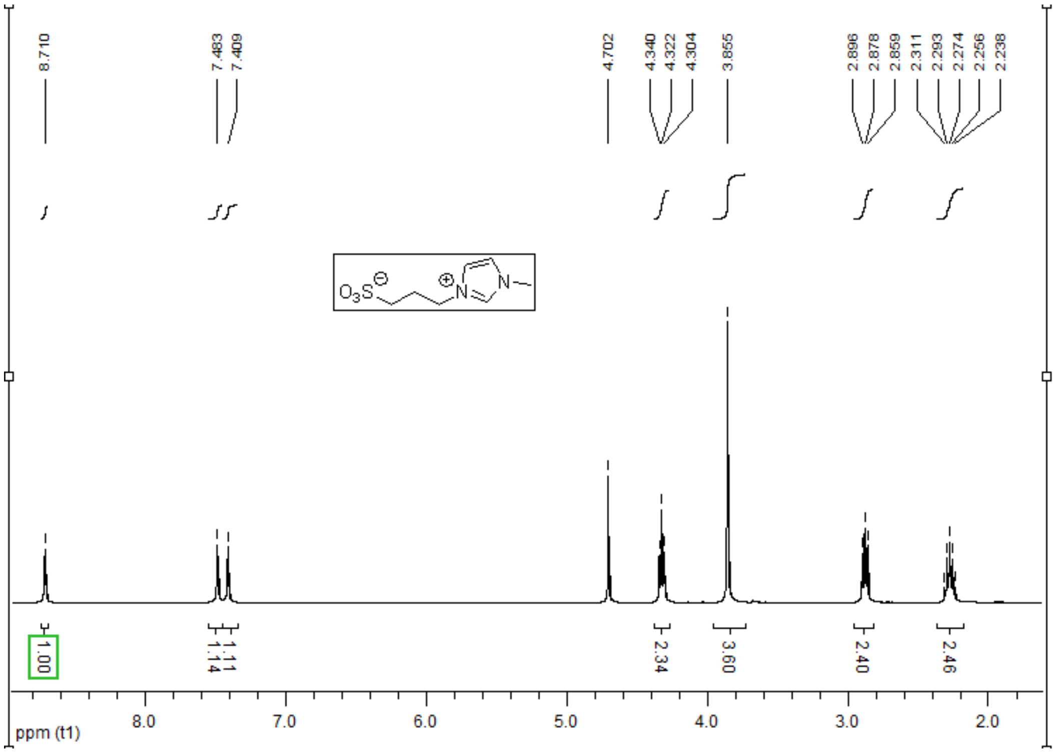
8.710
7.483
7.409
4.702
4.340
4.322
4.304
3.855
2.896
2.878
2.859
2.311
2.293
2.274
2.256
2.238
1.00
1.14
1.11
2.34
3.60
2.40
2.46
8.0
7.0
6.0
5.0
4.0
3.0
2.0
ppm (t1)

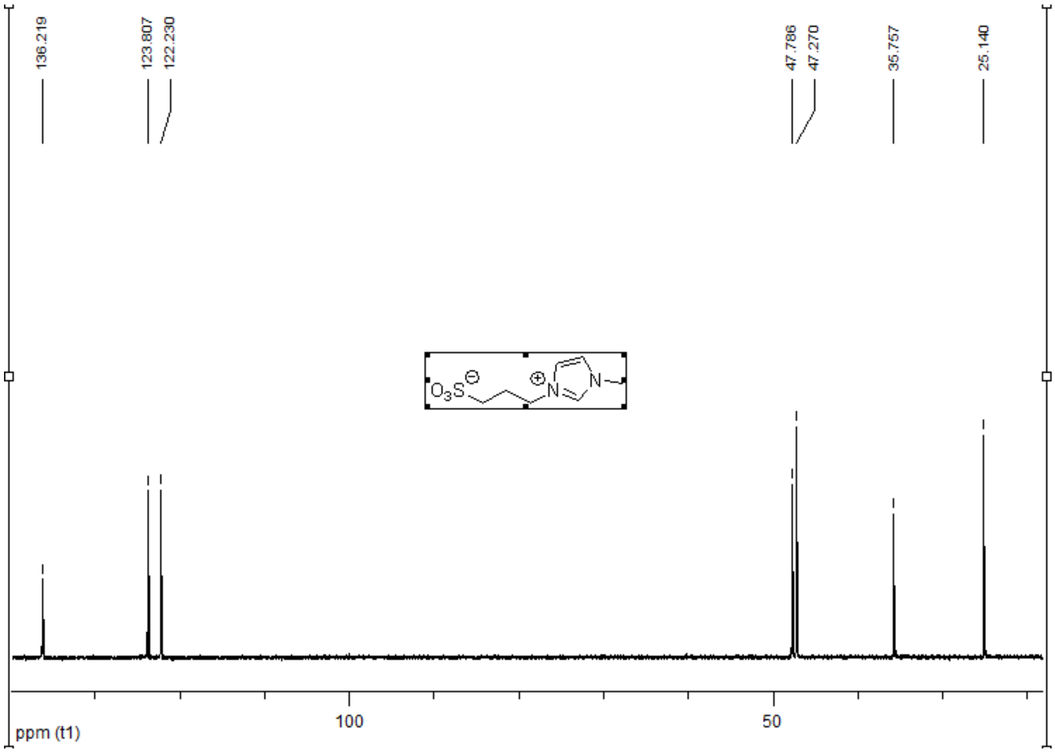
136.219
123.807
122.230
47.786
47.270
35.757
25.140
100
50
ppm (t1)

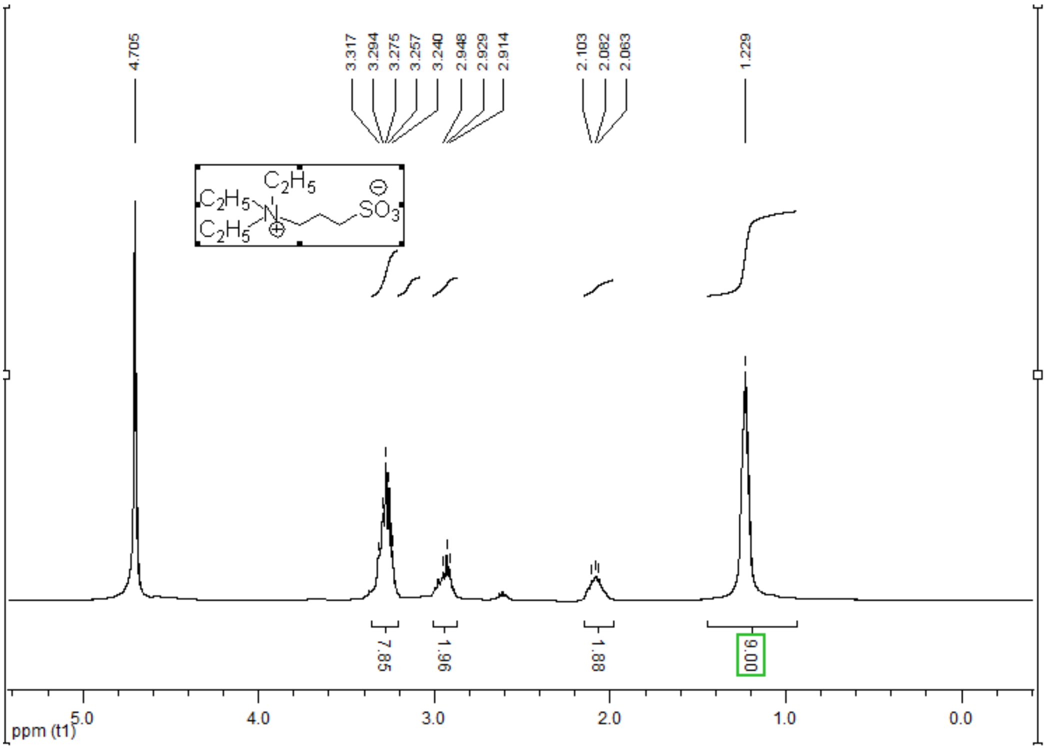
4.705
3.317
3.294
3.275
3.257
3.240
2.948
2.929
2.914
2.103
2.082
2.063
1.229
7.85
1.96
1.88
9.00
ppm (t1)
5.0
4.0
3.0
2.0
1.0
0.0

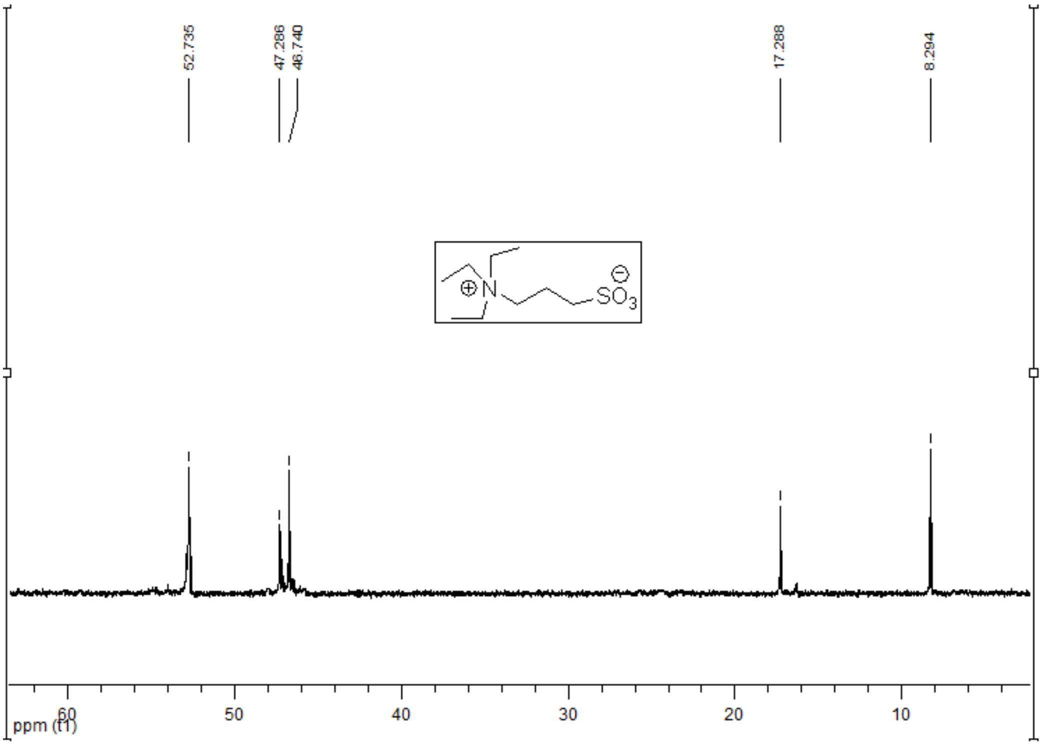
52.735
47.286
46.740
17.288
8.294
ppm (t1)
60
50
40
30
20
10

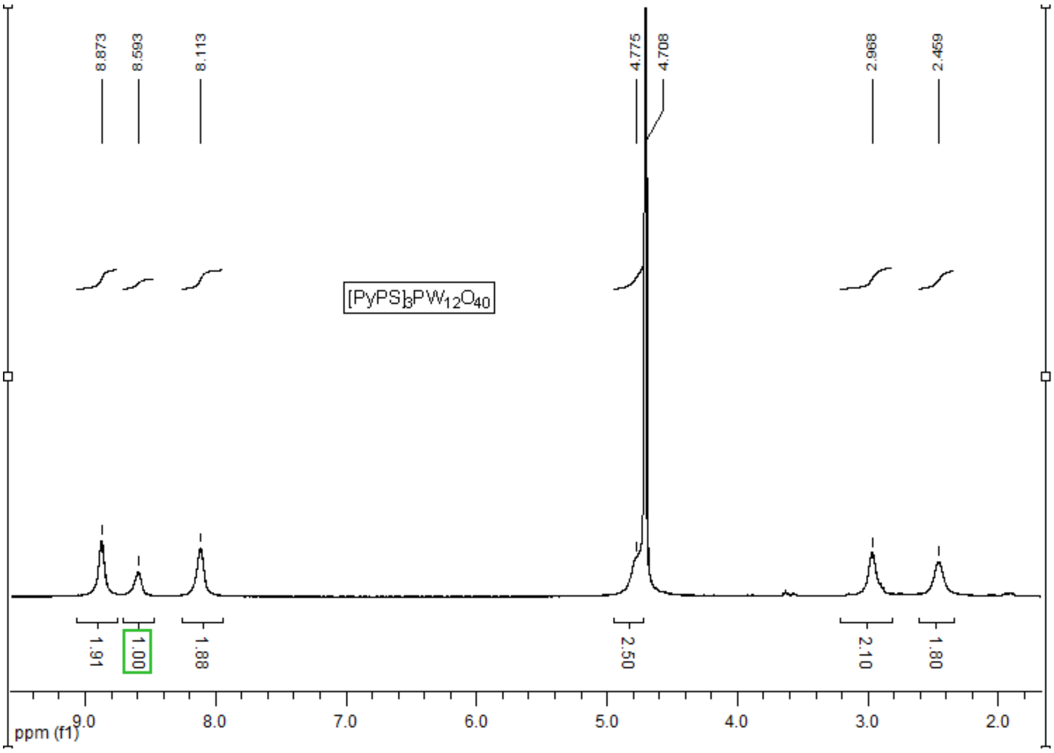
8.873
8.593
8.113
4.775
4.708
2.968
2.459
[PyPS]3PW12O40
1.91
1.00
1.88
2.50
2.10
1.80
ppm (f1)
9.0
8.0
7.0
6.0
5.0
4.0
3.0
2.0

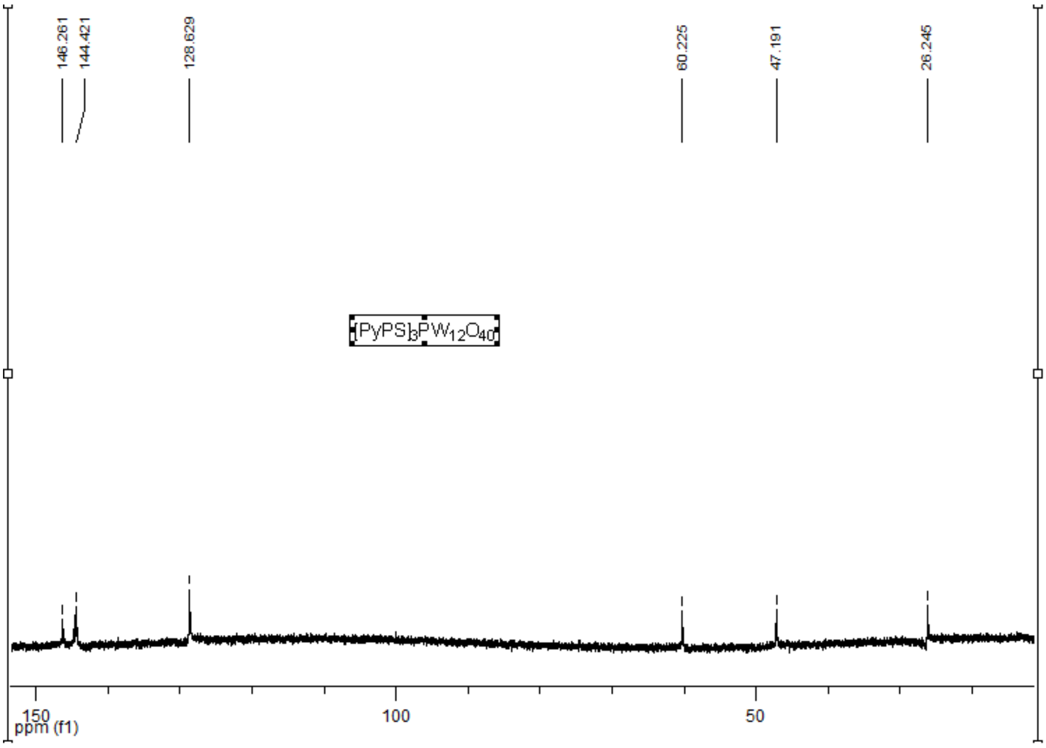
146.261
144.421
128.629
60.225
47.191
26.245
[PyPS]3PW12O40
150
ppm (f1)
100
50

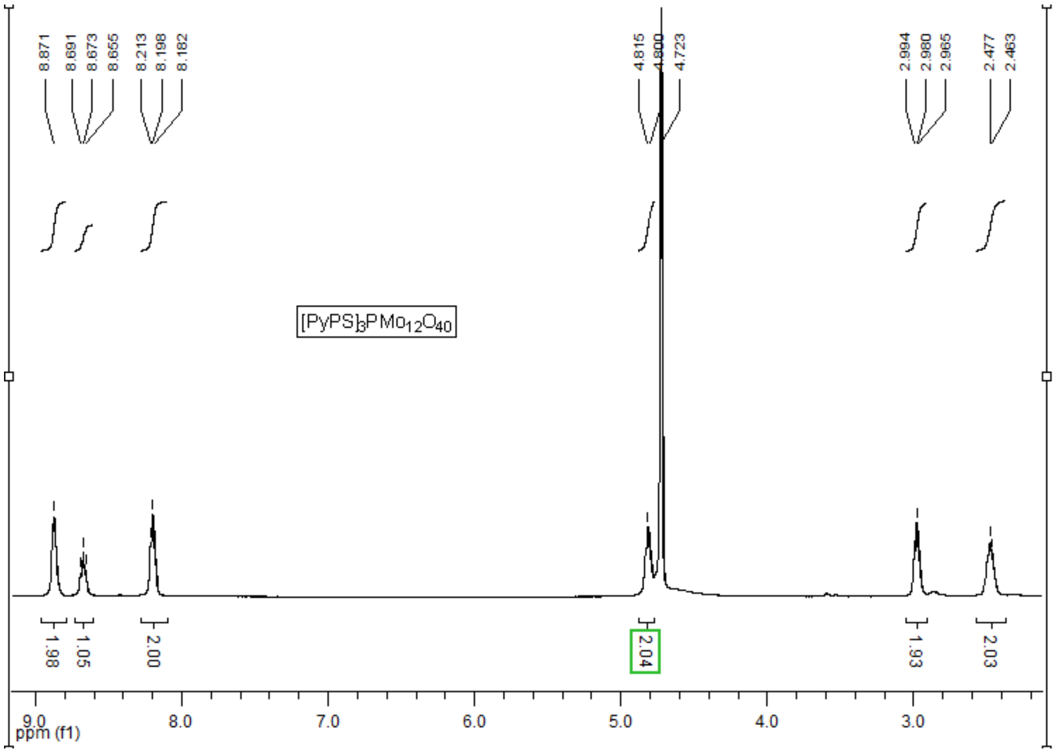
8.871
8.691
8.673
8.655
8.213
8.198
8.182
4.815
4.800
4.723
2.994
2.980
2.965
2.477
2.463
[PyPS]3PMo12O40
1.98
1.05
2.00
2.04
1.93
2.03
9.0
8.0
7.0
6.0
5.0
4.0
3.0
ppm (f1)

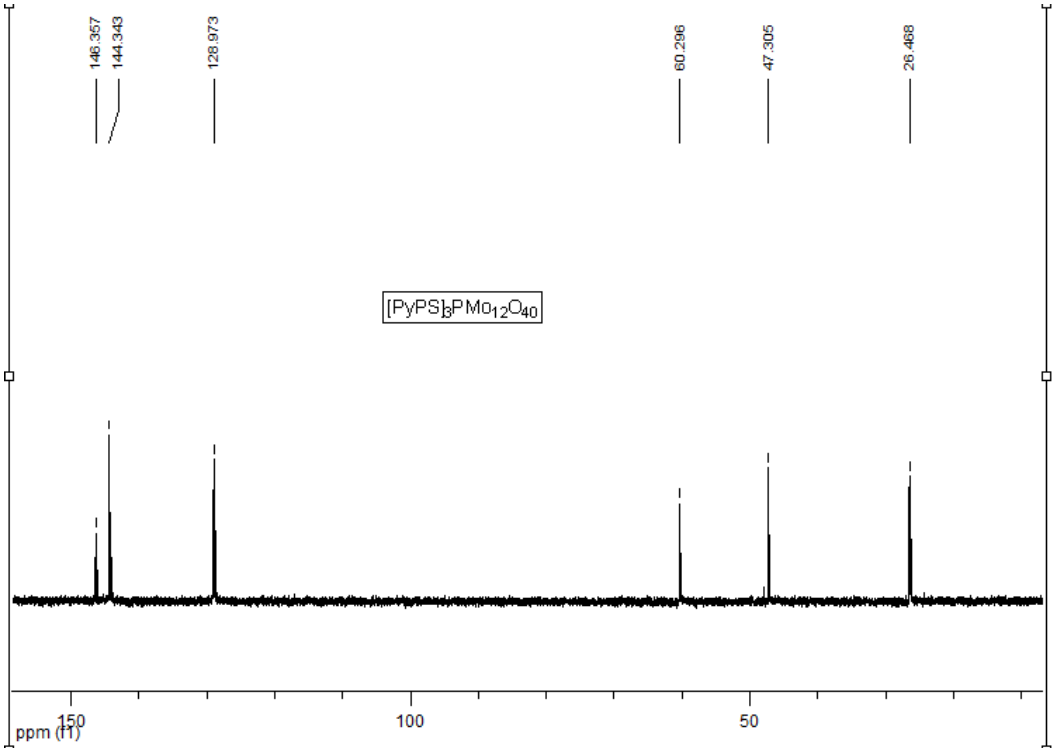
146.357
144.343
128.973
60.296
47.305
26.468
[PyPS]3PMo12O40
150
100
50
ppm (f1)

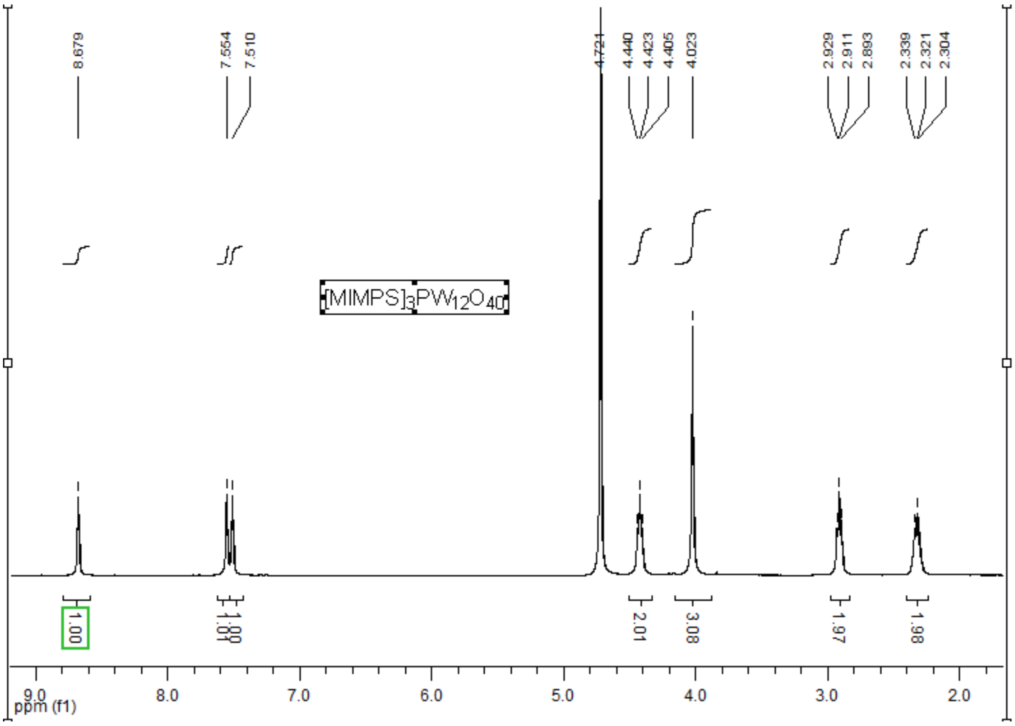
8.679
7.554
7.510
4.721
4.440
4.423
4.405
4.023
2.929
2.911
2.893
2.339
2.321
2.304
[MIMPS]3PW12O40
1.00
1.89
2.01
3.08
1.97
1.98
9.0
8.0
7.0
6.0
5.0
4.0
3.0
2.0
ppm (f1)

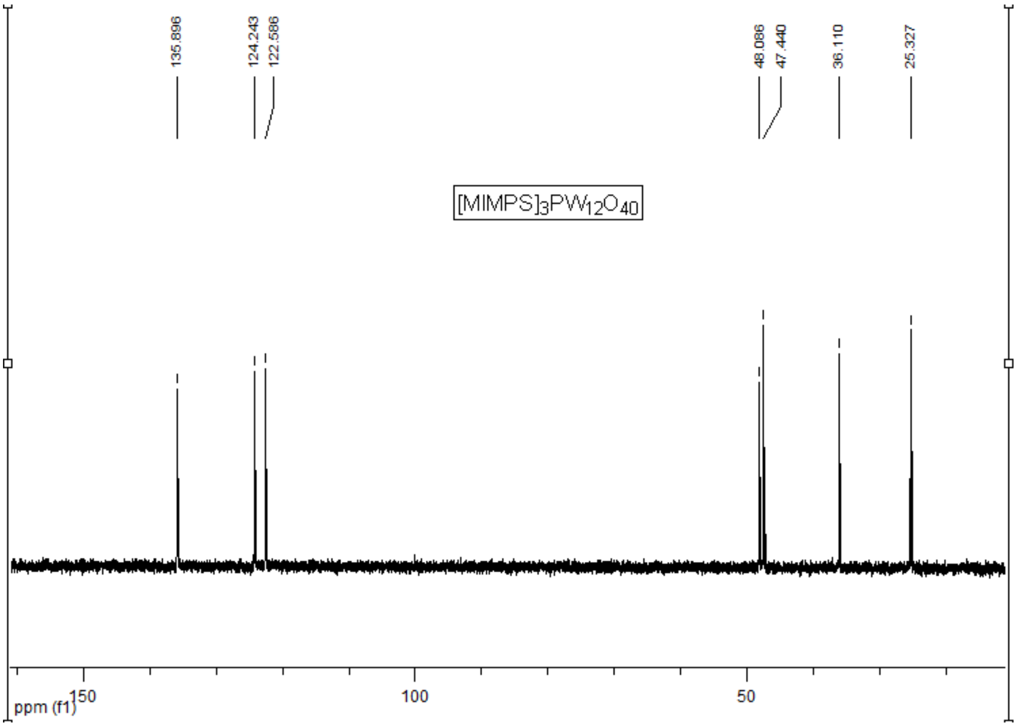
135.896
124.243
122.586
48.086
47.440
36.110
25.327
[MIMPS]3PW12O40
ppm (f1)
150
100
50

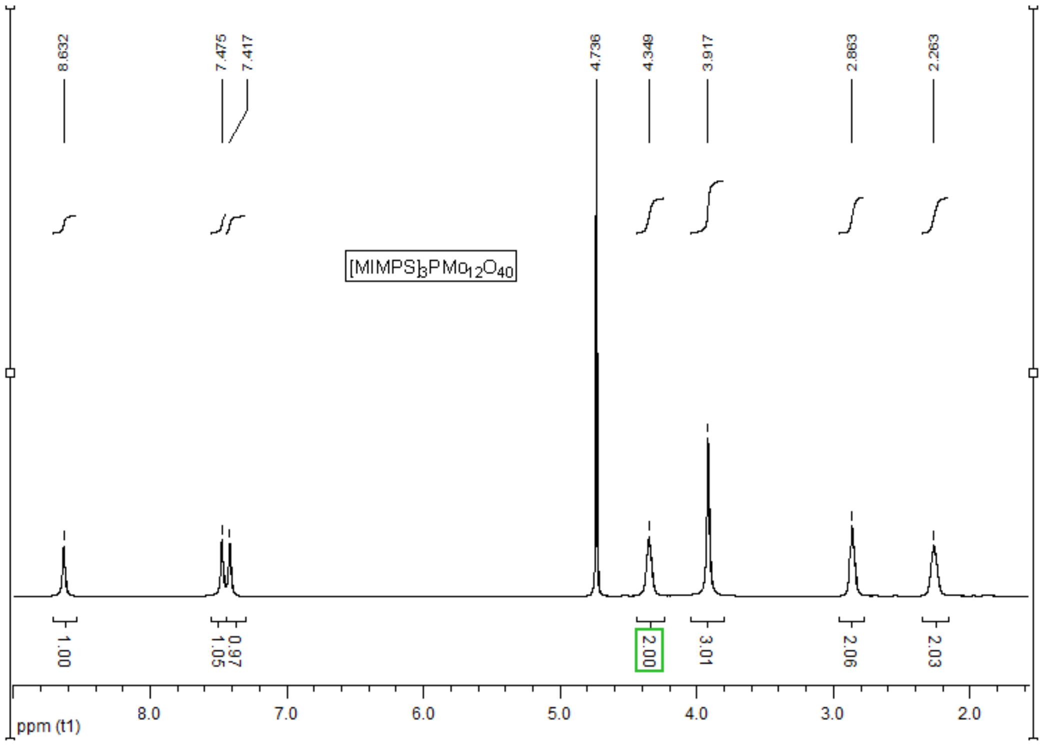
8.632
7.475
7.417
4.736
4.349
3.917
2.863
2.263
[MIMPS]3PMo12O40
1.00
1.05
0.97
2.00
3.01
2.06
2.03
8.0
7.0
6.0
5.0
4.0
3.0
2.0
ppm (t1)

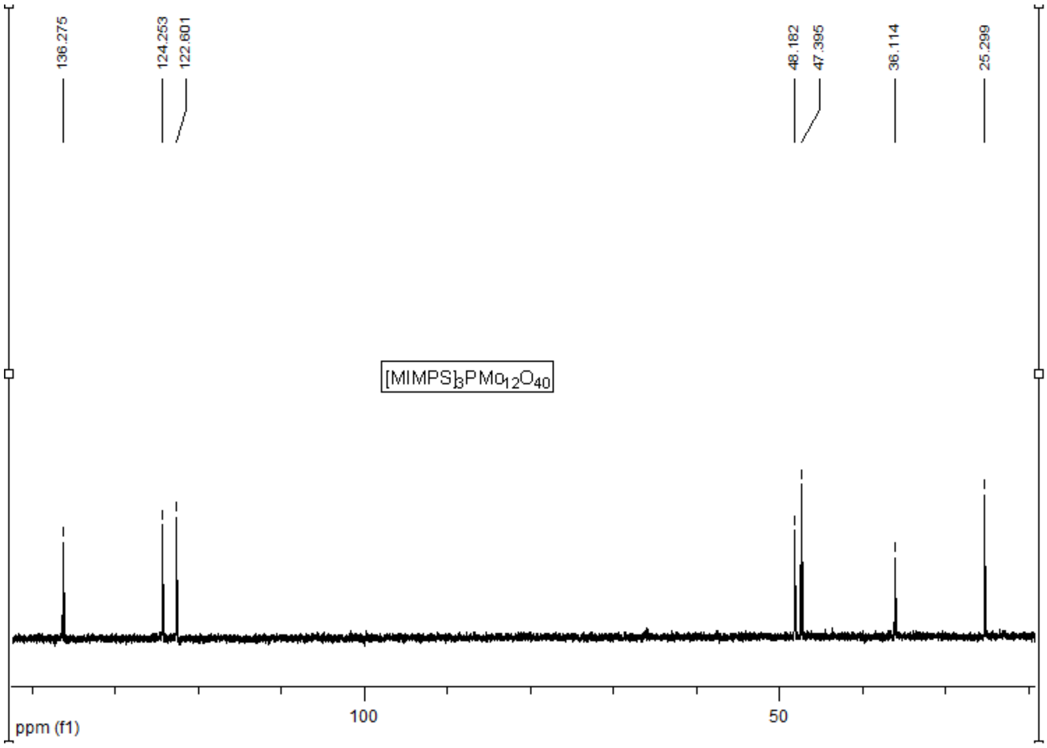
136.275
124.253
122.601
48.182
47.395
36.114
25.299
[MIMPS]3PMo12O40
100
50
ppm (f1)

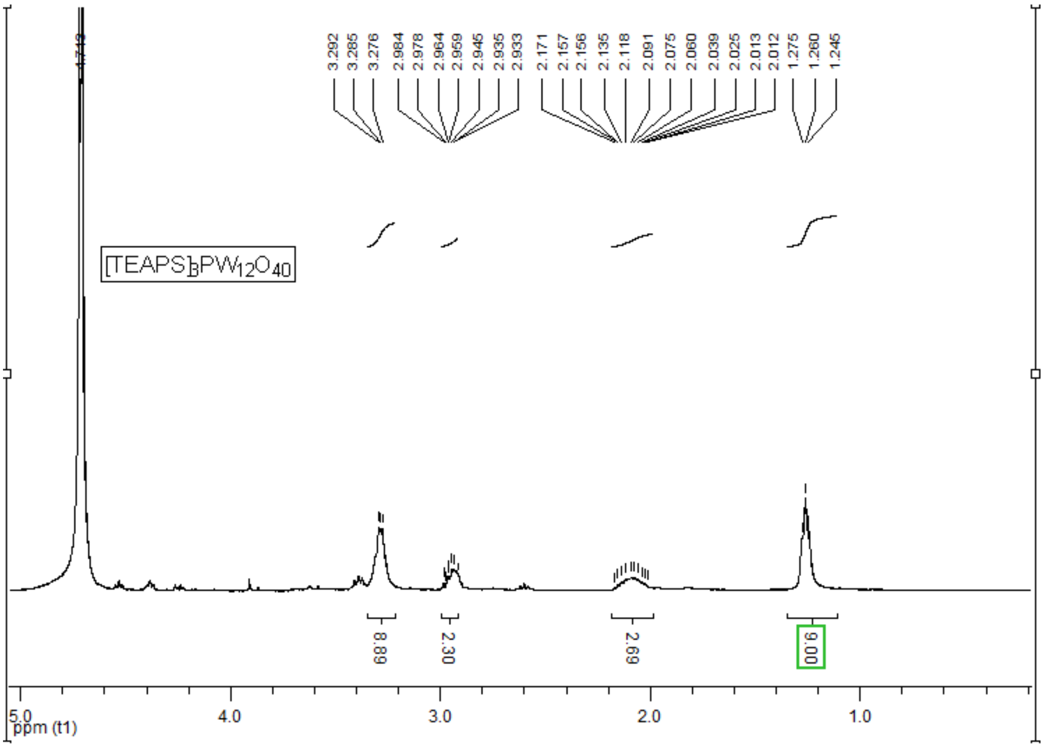
[TEAPS]3PW12O40
4.713
3.292
3.285
3.276
2.984
2.978
2.964
2.959
2.945
2.935
2.933
2.171
2.157
2.156
2.135
2.118
2.091
2.075
2.060
2.039
2.025
2.013
2.012
1.275
1.260
1.245
8.89
2.30
2.69
9.00
5.0
4.0
3.0
2.0
1.0
ppm (t1)

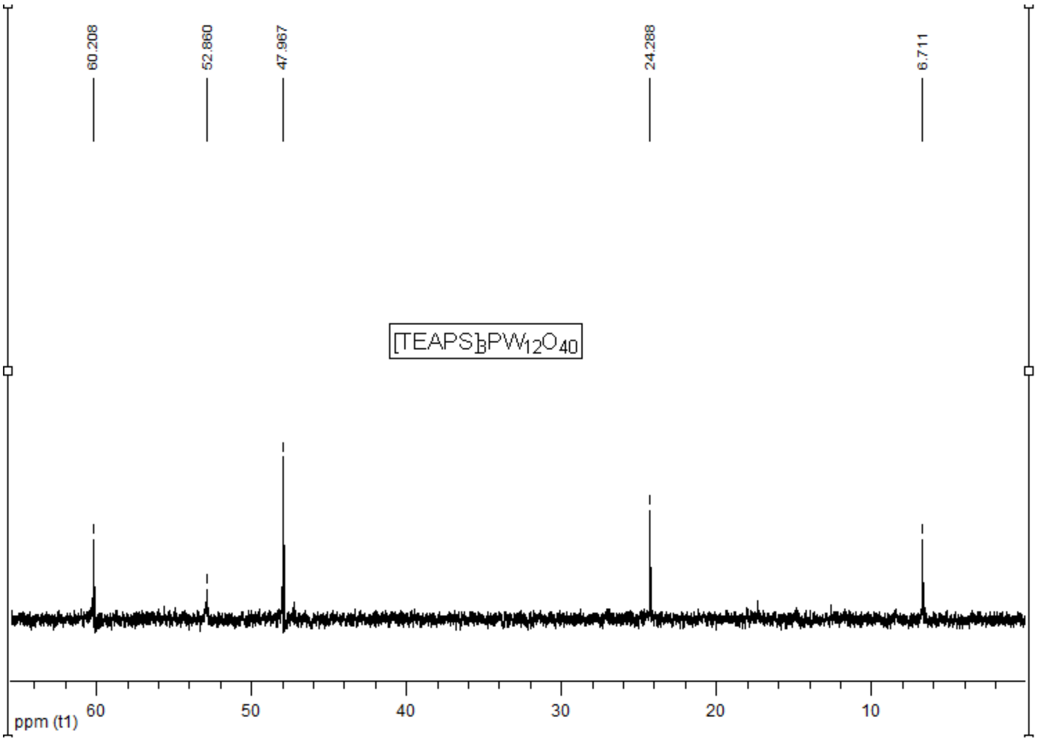
[TEAPS]3PW12O40
60.208
52.860
47.967
24.288
6.711
60
50
40
30
20
10
ppm (t1)

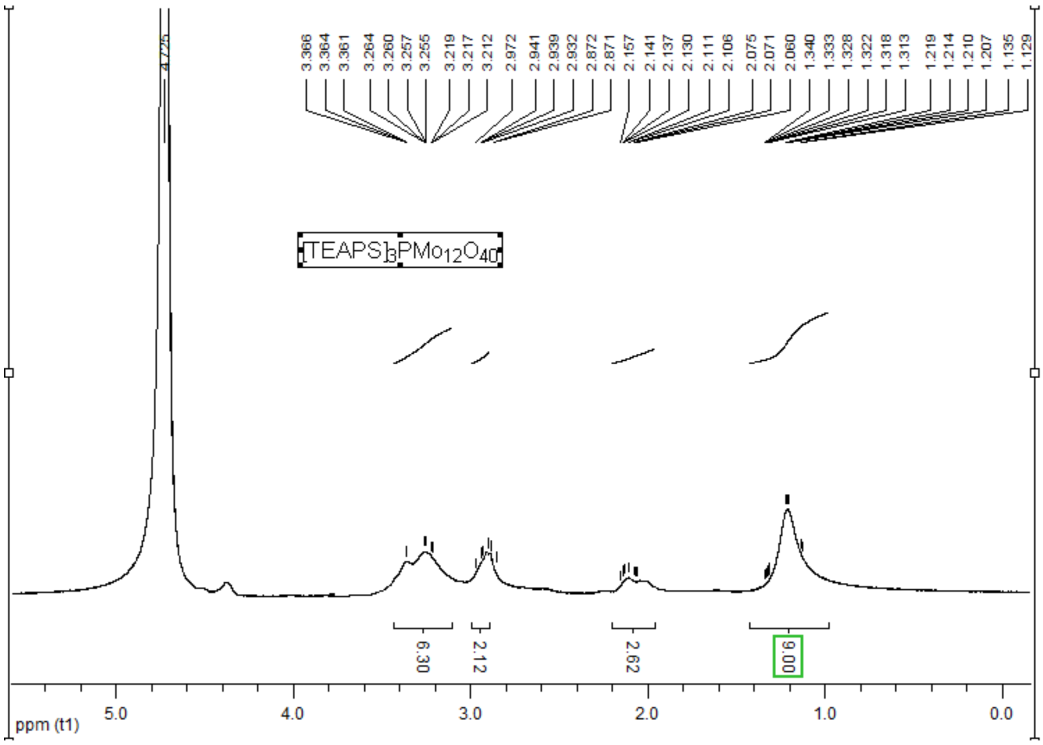
4.725
3.366
3.364
3.361
3.264
3.260
3.257
3.255
3.219
3.217
3.212
2.972
2.941
2.939
2.932
2.872
2.871
2.157
2.141
2.137
2.130
2.111
2.106
2.075
2.071
2.060
1.340
1.333
1.328
1.322
1.318
1.313
1.219
1.214
1.210
1.207
1.135
1.129
[TEAPS]3PMo12O40
6.30
2.12
2.62
9.00
ppm (t1)
5.0
4.0
3.0
2.0
1.0
0.0

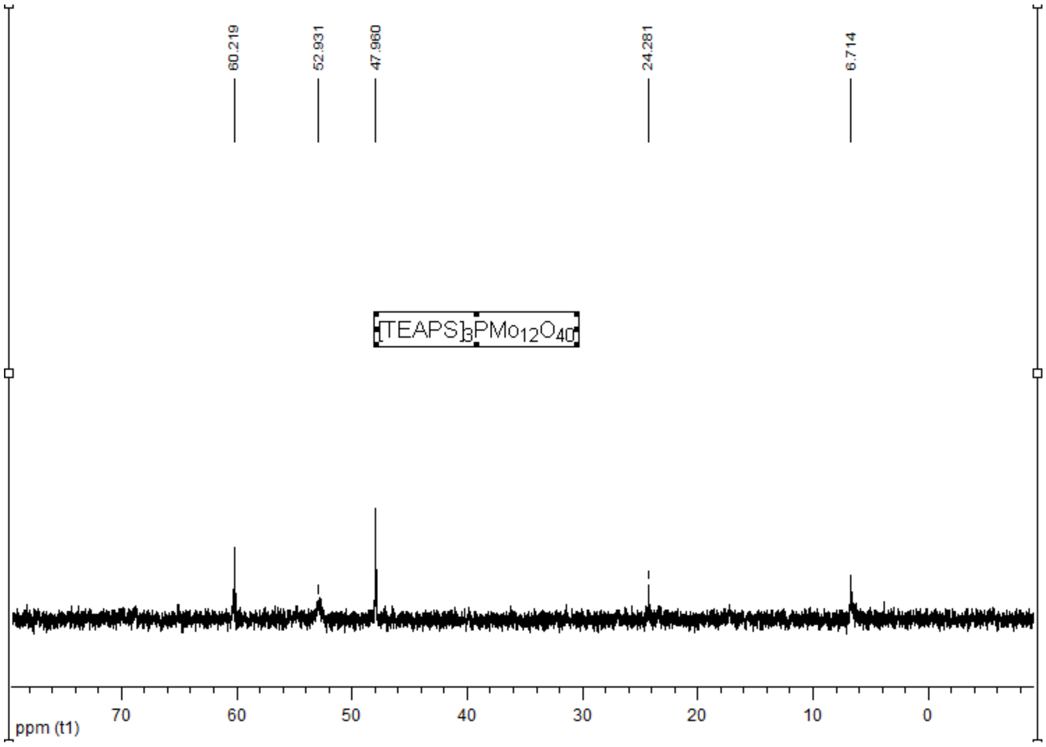
60.219
52.931
47.960
24.281
6.714
[TEAPS]3PMo12O40
ppm (t1)
70
60
50
40
30
20
10
0

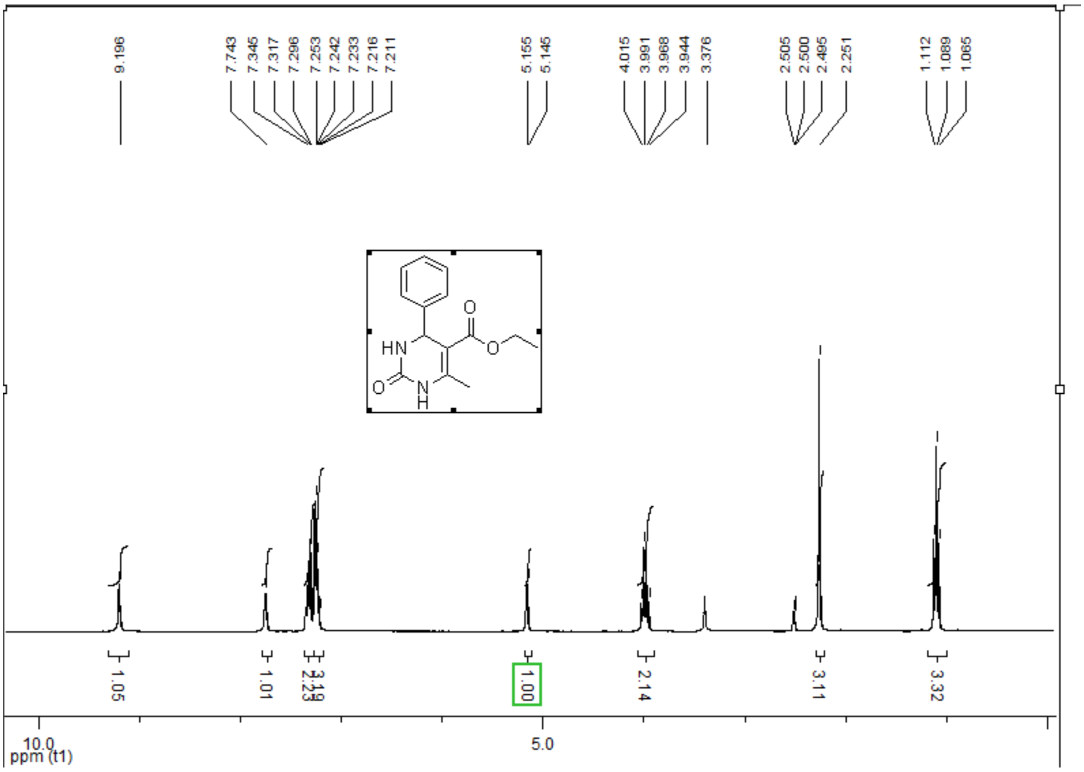
9.196
7.743 7.345 7.317 7.296 7.253 7.242 7.233 7.216 7.211
5.155 5.145
4.015 3.991 3.968 3.944 3.376
2.505 2.500 2.495 2.251
1.112 1.089 1.065
HN
O
O
N
H
O
1.05
1.01
2.23
3.19
1.00
2.14
3.11
3.32
10.0
5.0
ppm (t1)

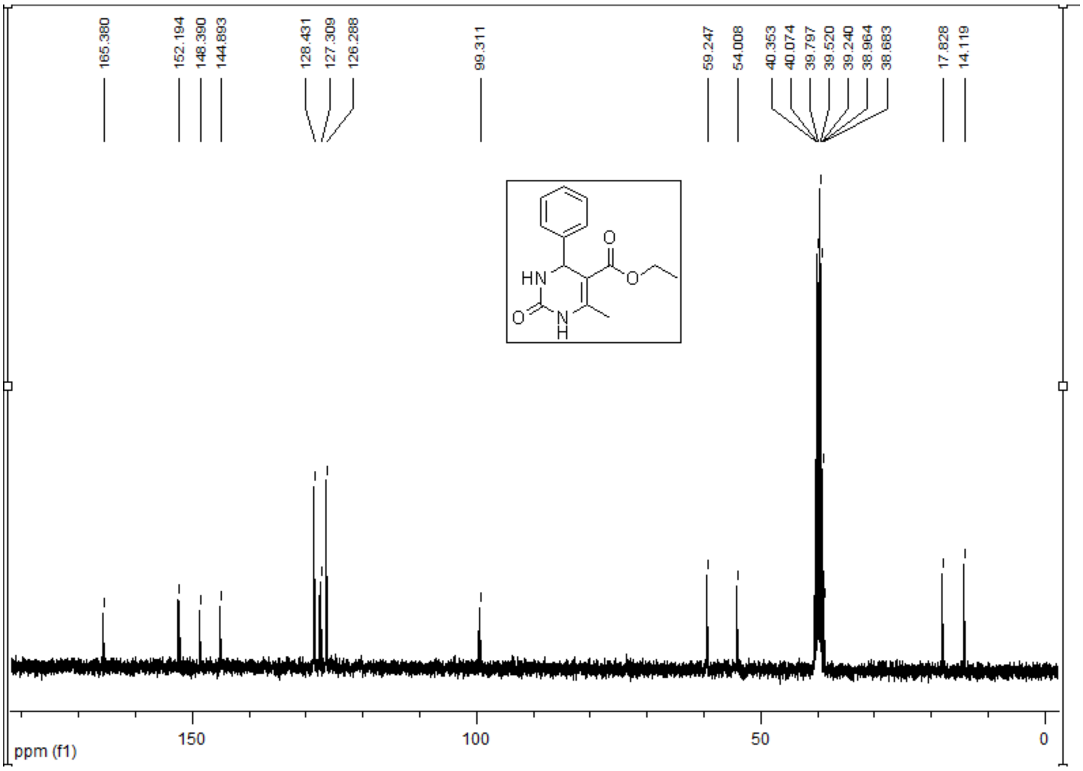
165.380
152.194 148.390 144.893
128.431 127.309 126.288
99.311
59.247 54.008
40.353 40.074 39.797 39.520 39.240 38.964 38.683
17.828 14.119
HN
O
O
N
H
O
150
100
50
0
ppm (f1)

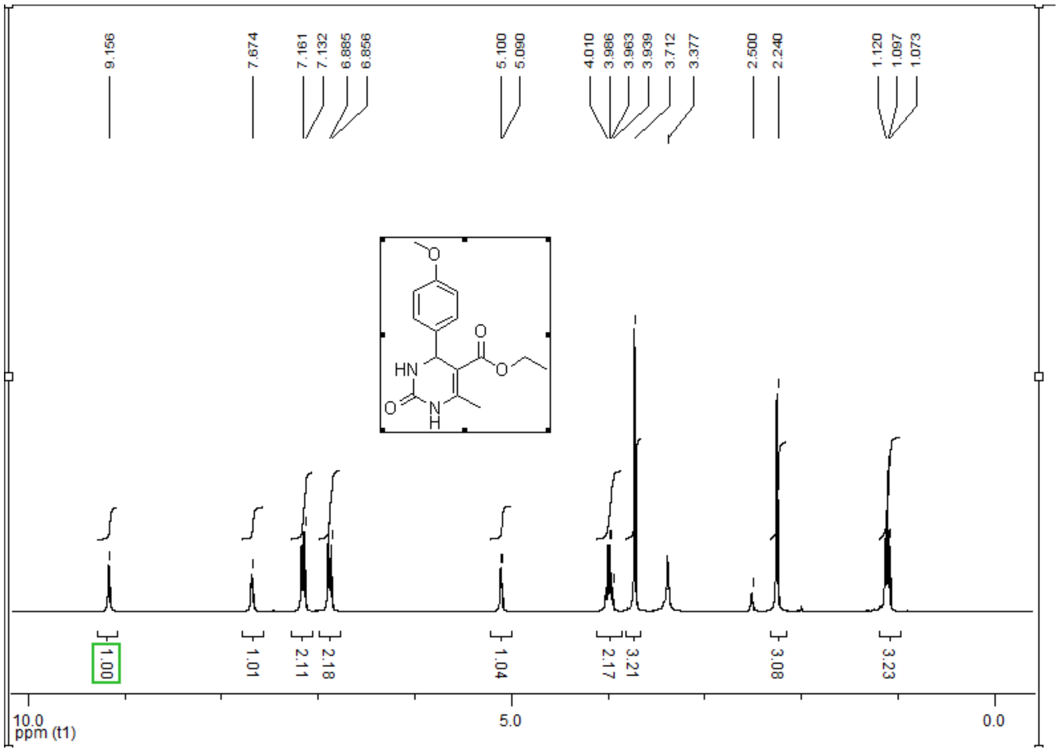

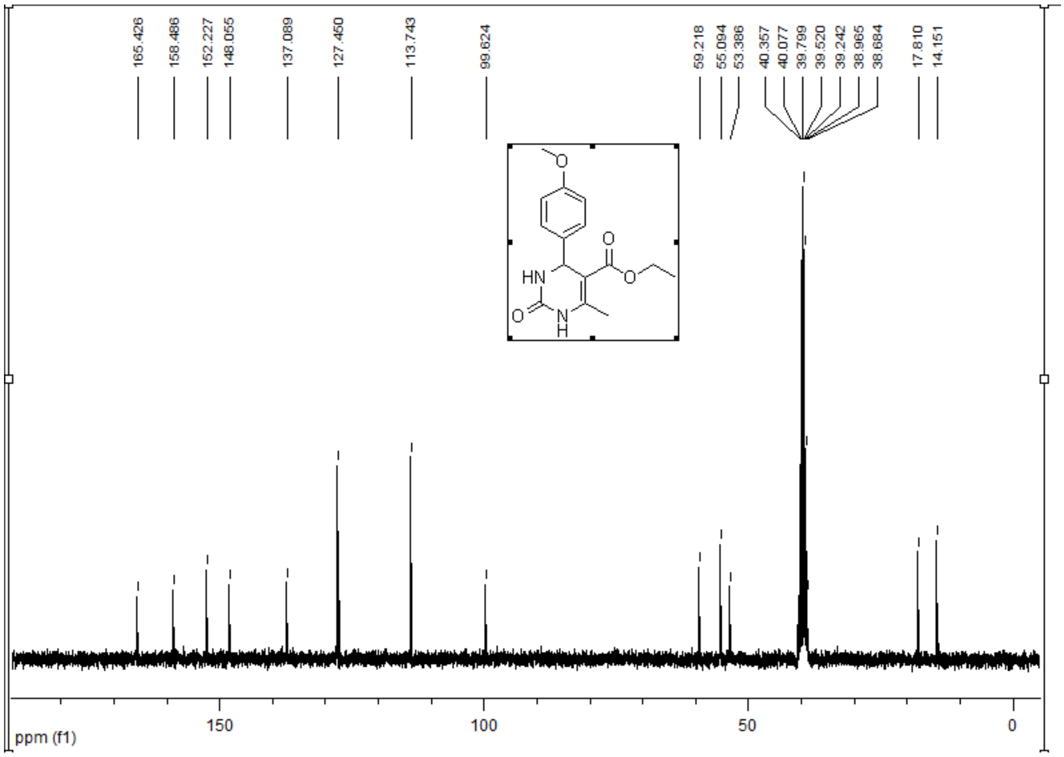

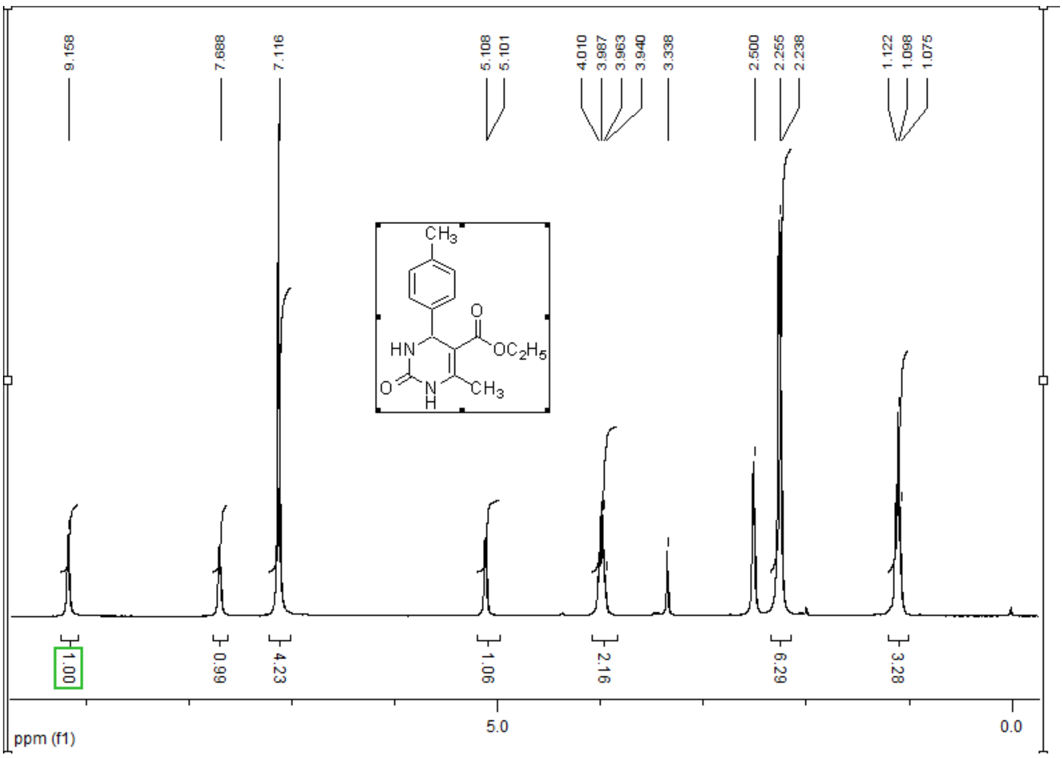
9.158
7.688
7.116
5.108
5.101
4.010
3.987
3.963
3.940
3.338
2.500
2.255
2.238
1.122
1.098
1.075
CH3
O
HN
OC2H5
O
N
H
CH3
1.00
0.99
4.23
1.06
2.16
6.29
3.28
5.0
0.0
ppm (f1)

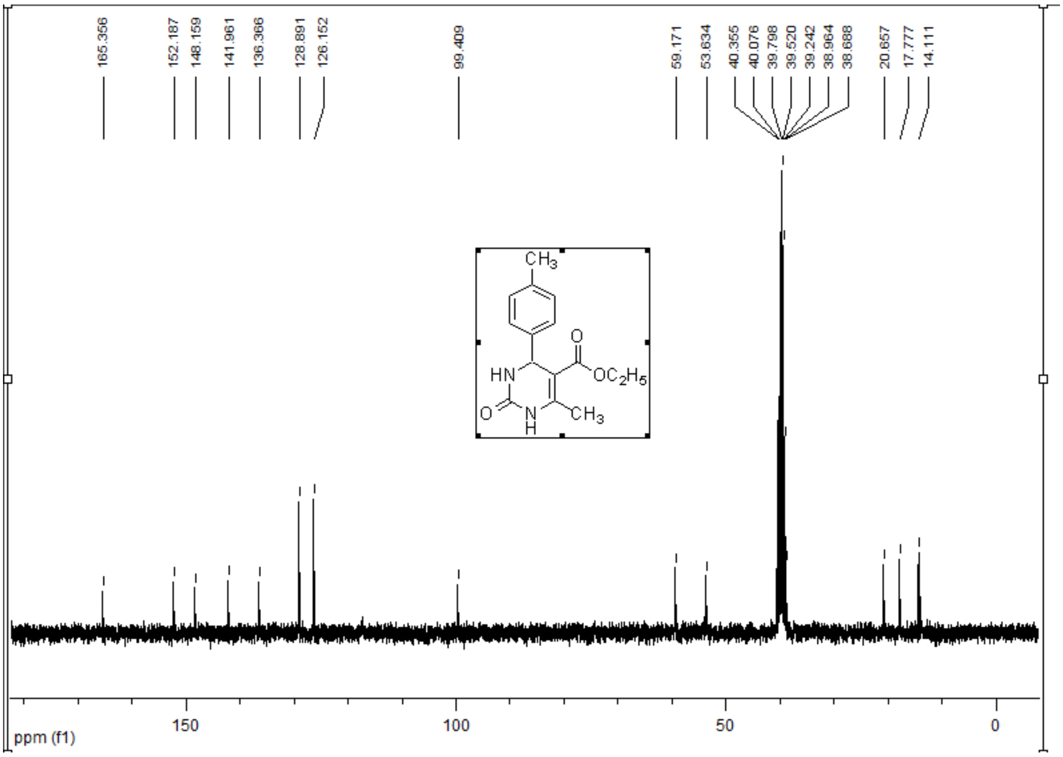
165.356
152.187
148.159
141.961
136.366
128.891
126.152
99.409
59.171
53.634
40.355
40.076
39.798
39.520
39.242
38.964
38.688
20.657
17.777
14.111
CH3
O
HN
OC2H5
O
N
H
CH3
150
100
50
0
ppm (f1)

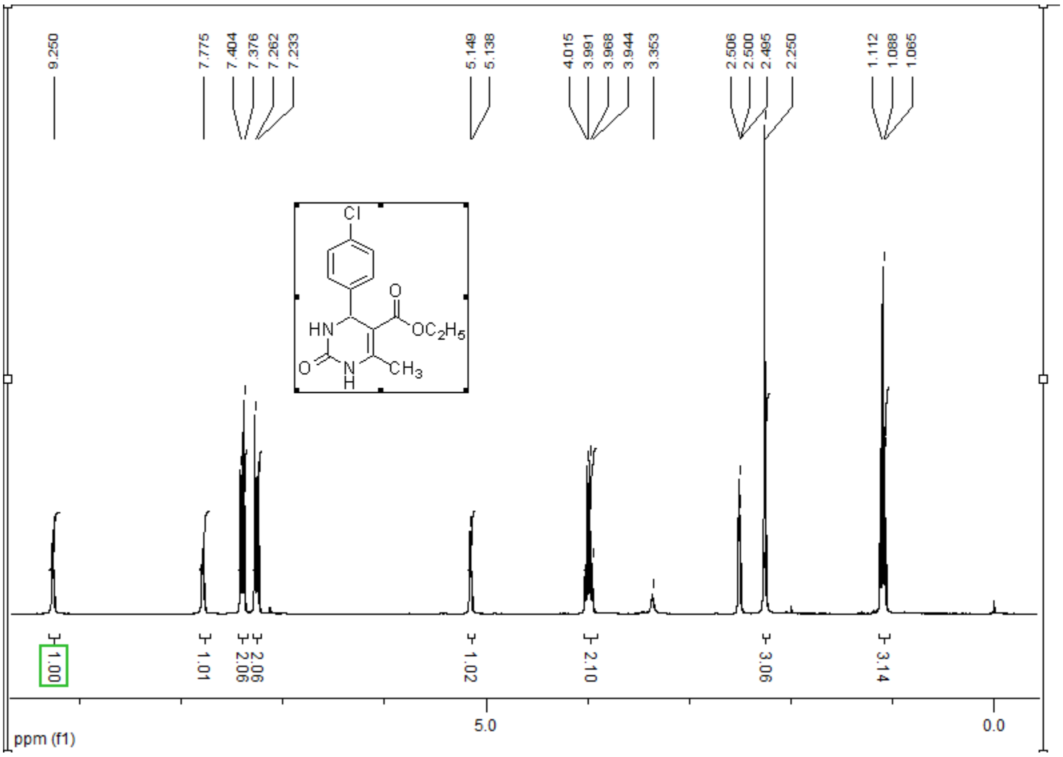
9.250
7.775
7.404
7.376
7.262
7.233
5.149
5.138
4.015
3.991
3.968
3.944
3.353
2.506
2.500
2.495
2.250
1.112
1.088
1.065
Cl
O
HN
OC2H5
O
N
H
CH3
1.00
1.01
2.06
2.06
1.02
2.10
3.06
3.14
5.0
0.0
ppm (f1)

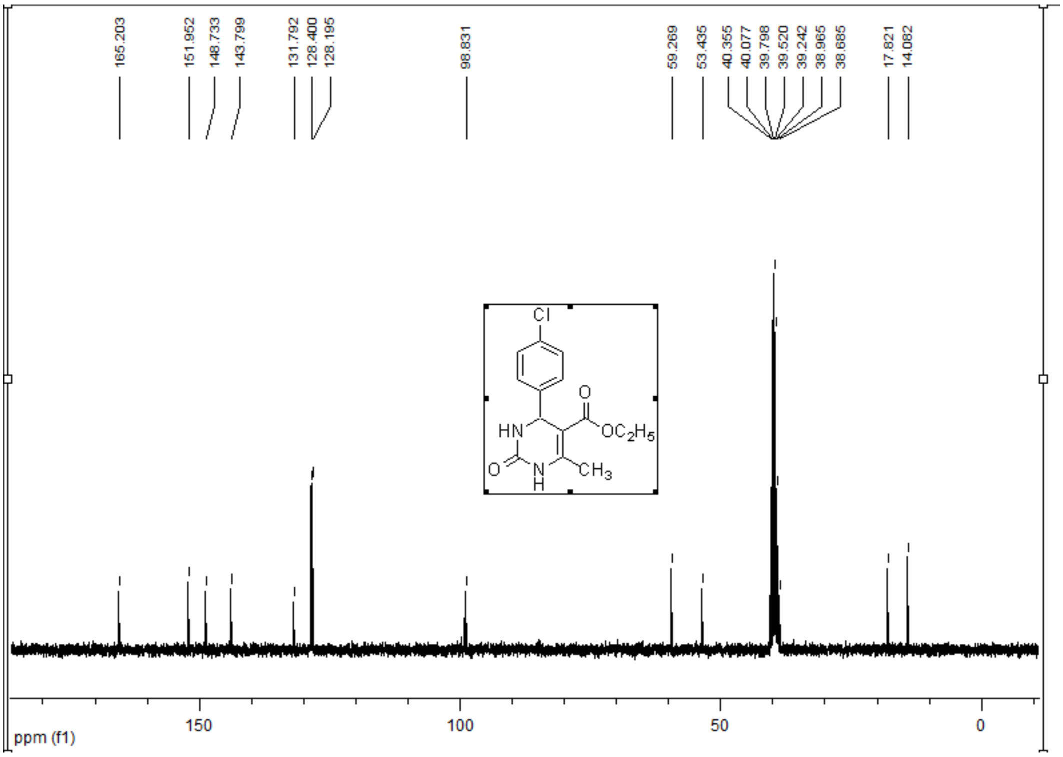
165.203
151.952
148.733
143.799
131.792
128.400
128.195
98.831
59.269
53.435
40.355
40.077
39.798
39.520
39.242
38.965
38.685
17.821
14.082
Cl
O
HN
OC2H5
O
N
H
CH3
150
100
50
0
ppm (f1)

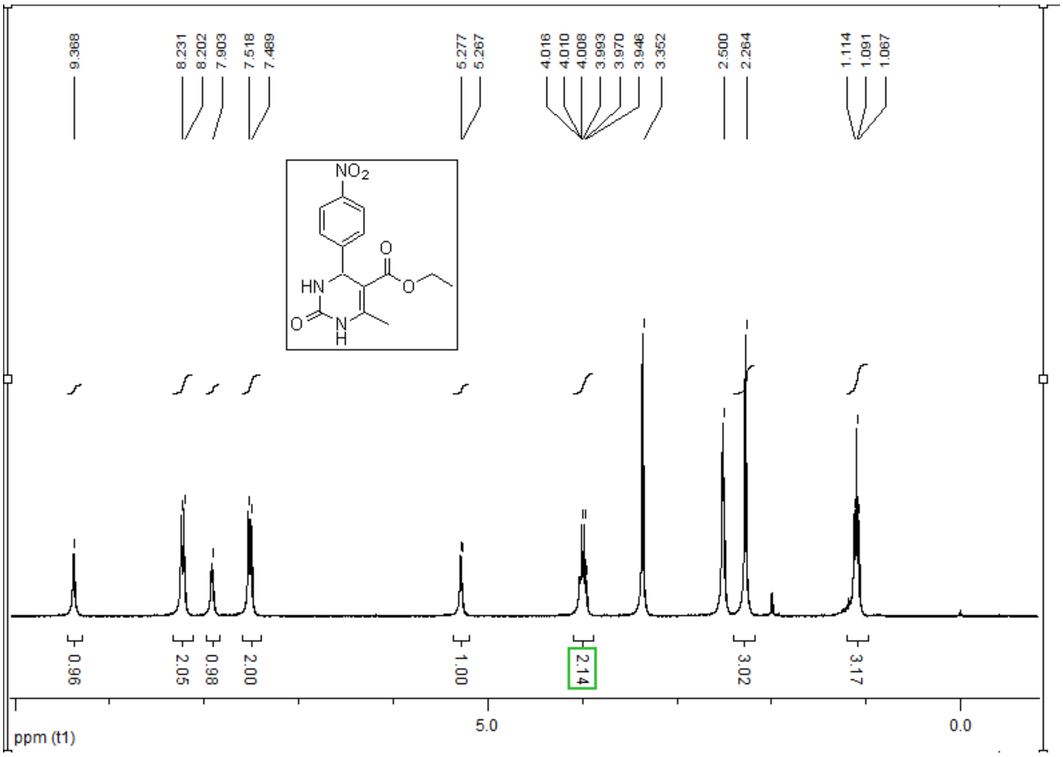
9.368
8.231
8.202
7.903
7.518
7.489
5.277
5.267
4.016
4.010
4.008
3.993
3.970
3.946
3.352
2.500
2.264
1.114
1.091
1.067
NO_2
O
HN
O
O
N
H
0.96
2.05
0.98
2.00
1.00
2.14
3.02
3.17
5.0
0.0
ppm (t1)

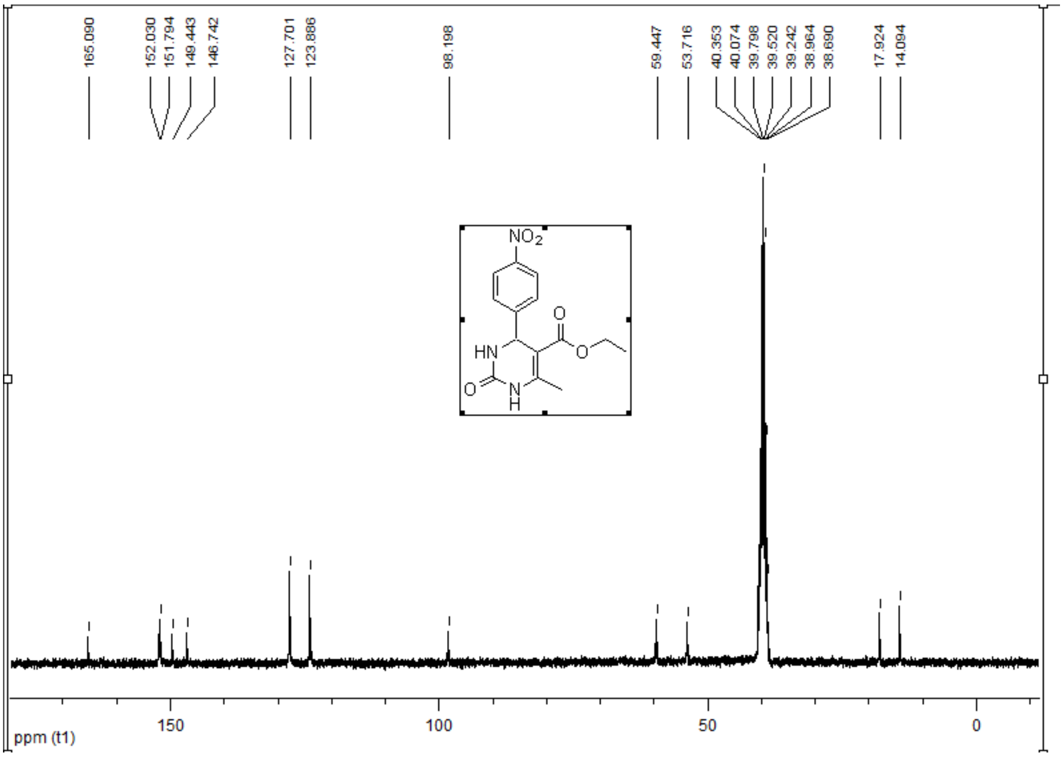
165.090
152.030
151.794
149.443
146.742
127.701
123.886
98.198
59.447
53.716
40.353
40.074
39.798
39.520
39.242
38.964
38.690
17.924
14.094
NO_2
O
HN
O
O
N
H
150
100
50
0
ppm (t1)

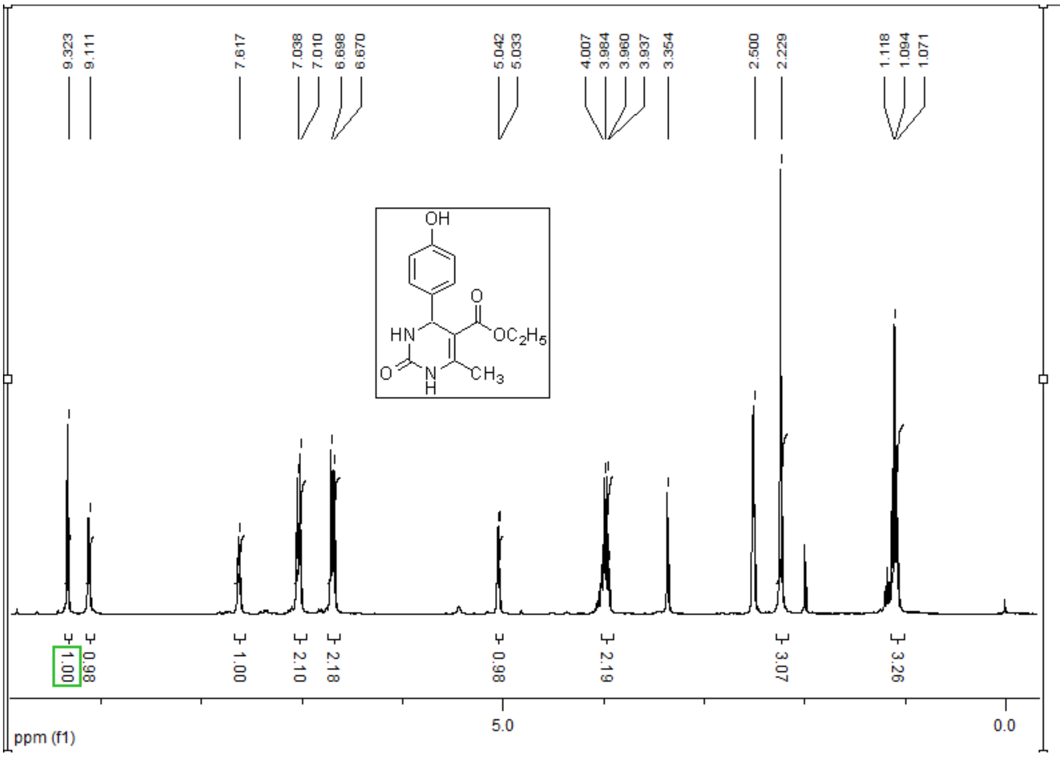
9.323
9.111
7.617
7.038
7.010
6.698
6.670
5.042
5.033
4.007
3.984
3.960
3.937
3.354
2.500
2.229
1.118
1.094
1.071
OH
O
HN
OC2H5
O
N
H
CH3
1.00
0.98
1.00
2.10
2.18
0.98
2.19
3.07
3.26
5.0
0.0
ppm (f1)

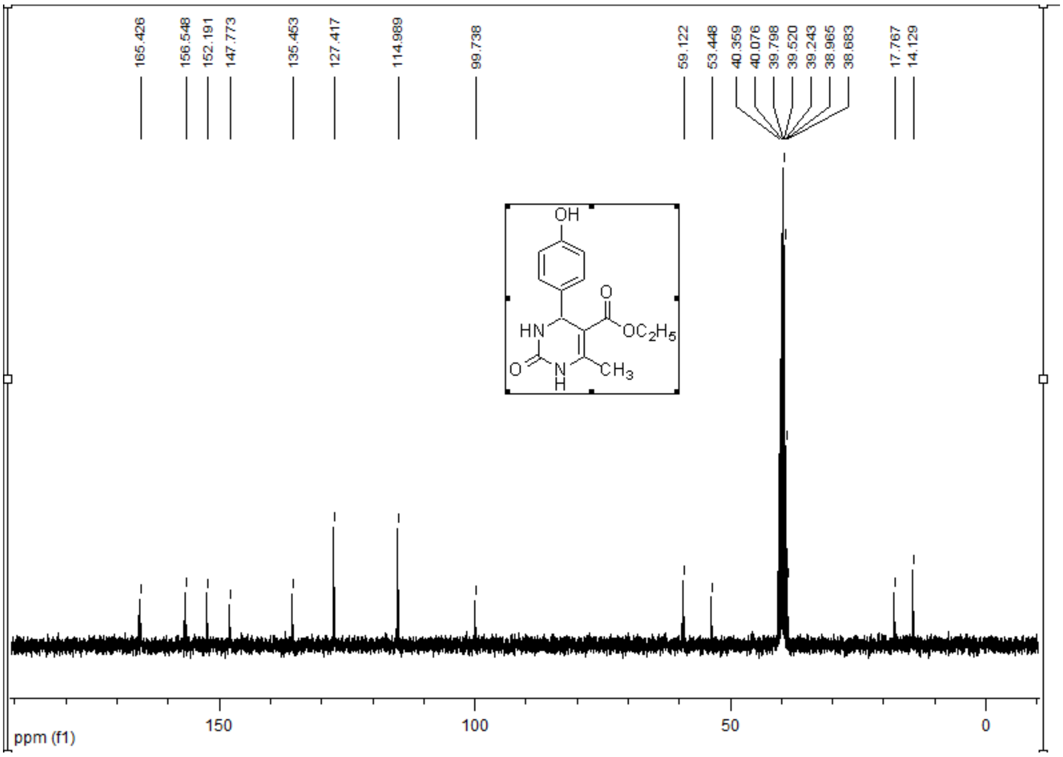
165.426
156.548
152.191
147.773
135.453
127.417
114.989
99.738
59.122
53.448
40.359
40.076
39.798
39.520
39.243
38.965
38.683
17.767
14.129
OH
O
HN
OC2H5
O
N
H
CH3
150
100
50
0
ppm (f1)

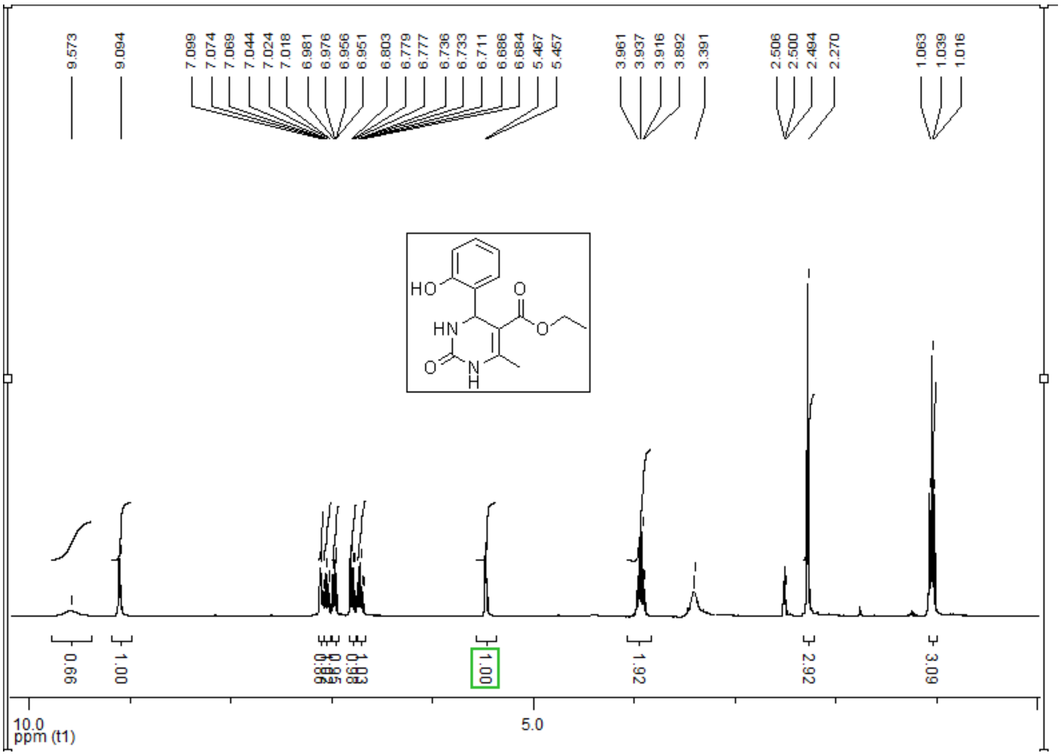
HO
HN
O
N
H
O
O
ppm (t1)
10.0
5.0

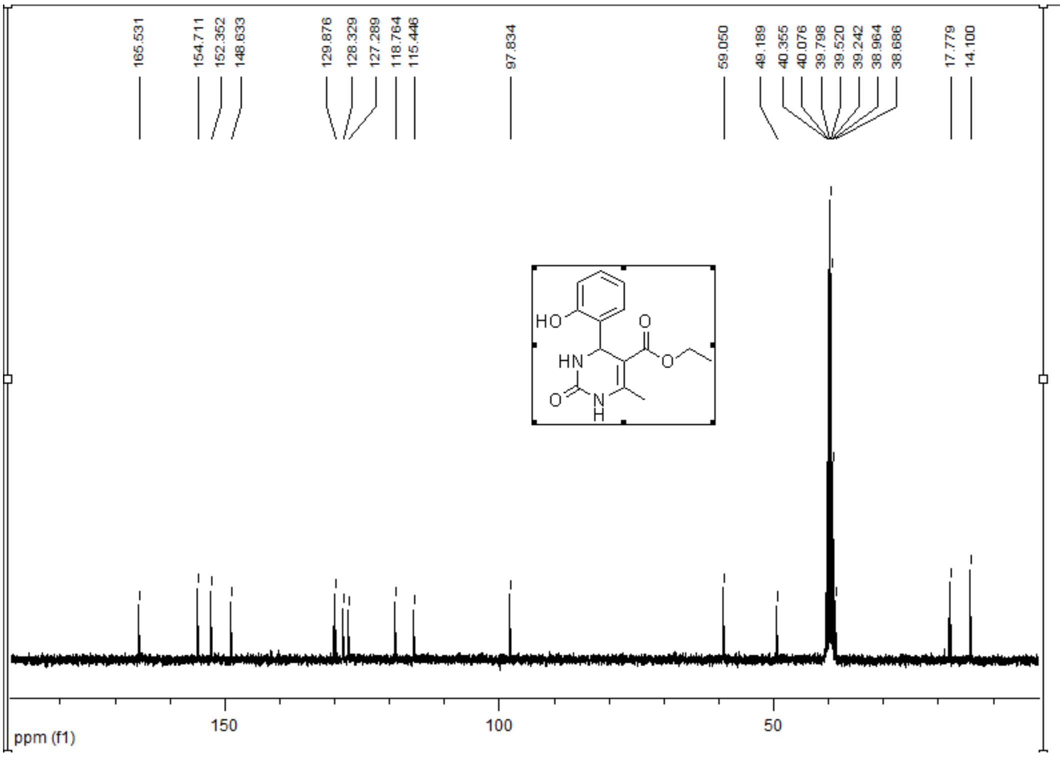
HO
HN
O
N
H
O
O
ppm (f1)
150
100
50

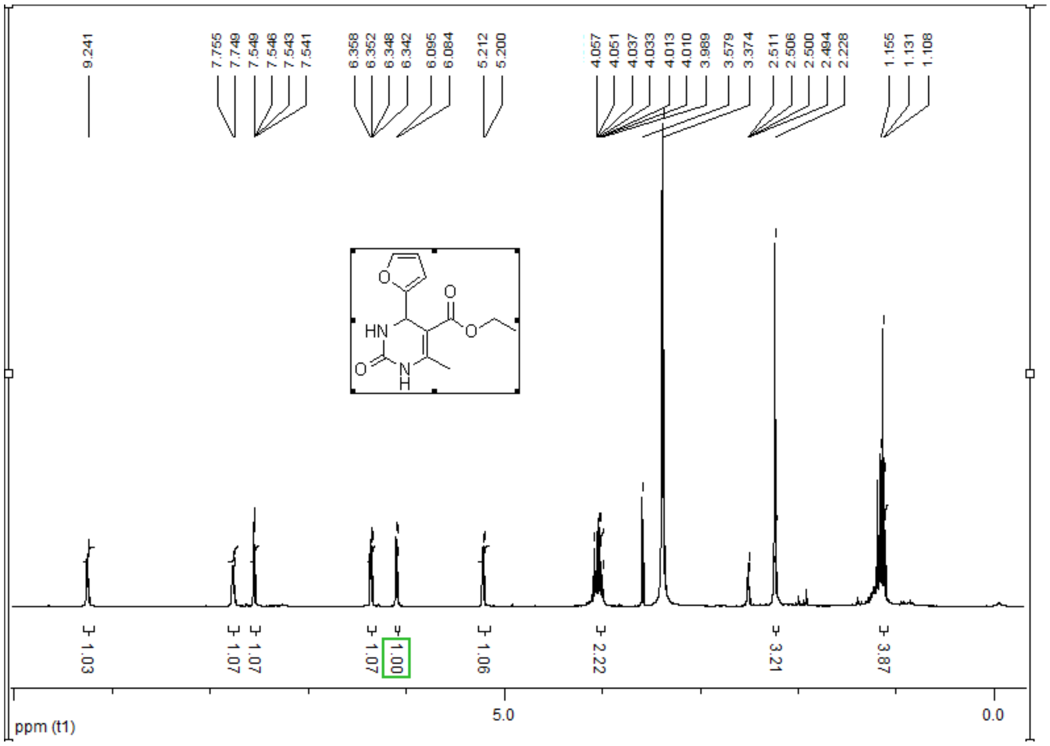
9.241
7.755
7.749
7.549
7.546
7.543
7.541
6.358
6.352
6.348
6.342
6.095
6.084
5.212
5.200
4.057
4.051
4.037
4.033
4.013
4.010
3.989
3.579
3.374
2.511
2.506
2.500
2.494
2.228
1.155
1.131
1.108
HN
O
O
O
O
N
H
1.03
1.07
1.07
1.07
1.00
1.06
2.22
3.21
3.87
5.0
0.0
ppm (t1)

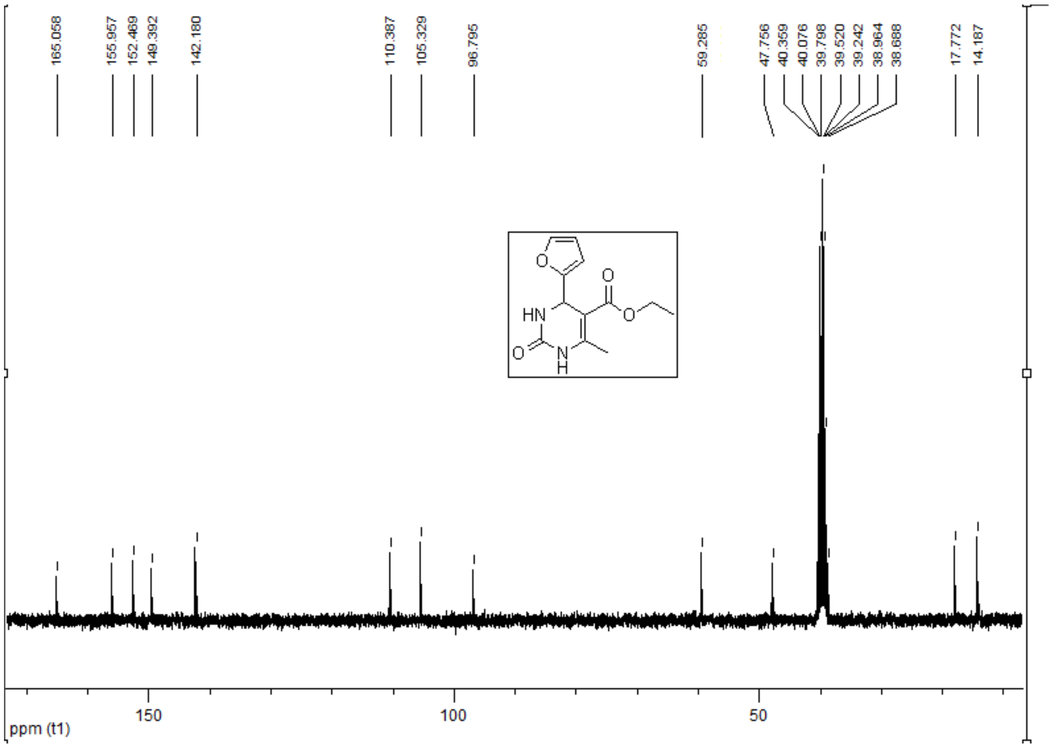
165.058
155.957
152.469
149.392
142.180
110.387
105.329
96.795
59.285
47.756
40.359
40.076
39.798
39.520
39.242
38.964
38.688
17.772
14.187
HN
O
O
O
O
N
H
150
100
50
ppm (t1)

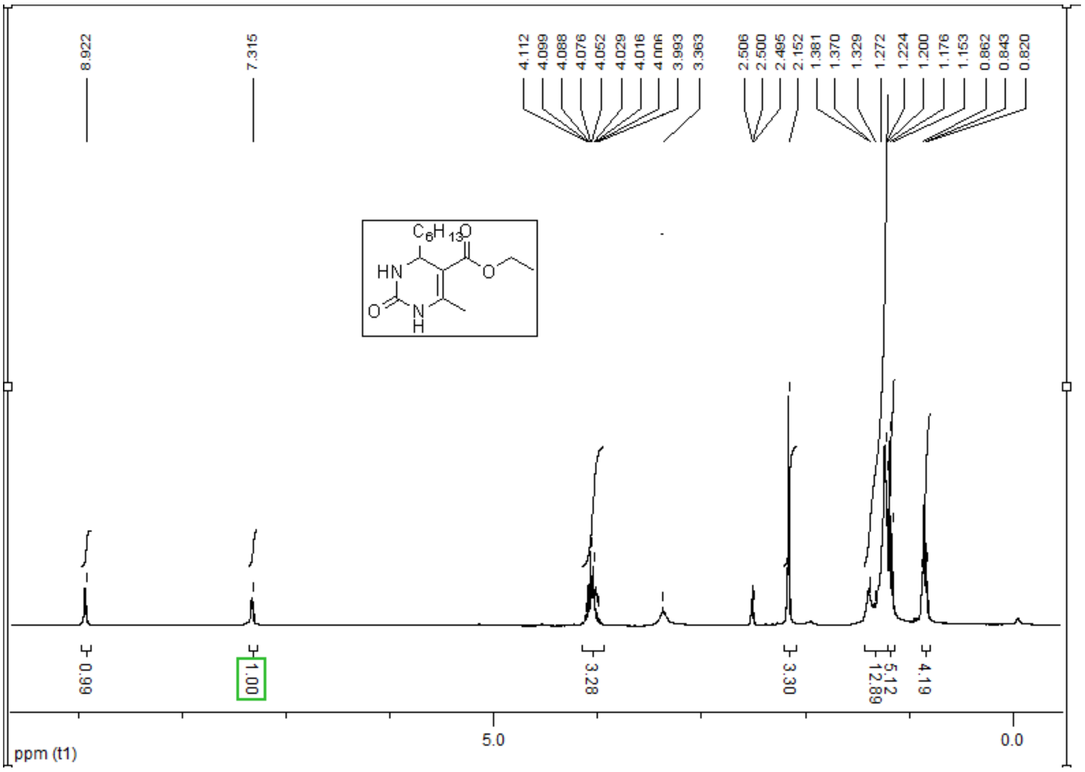

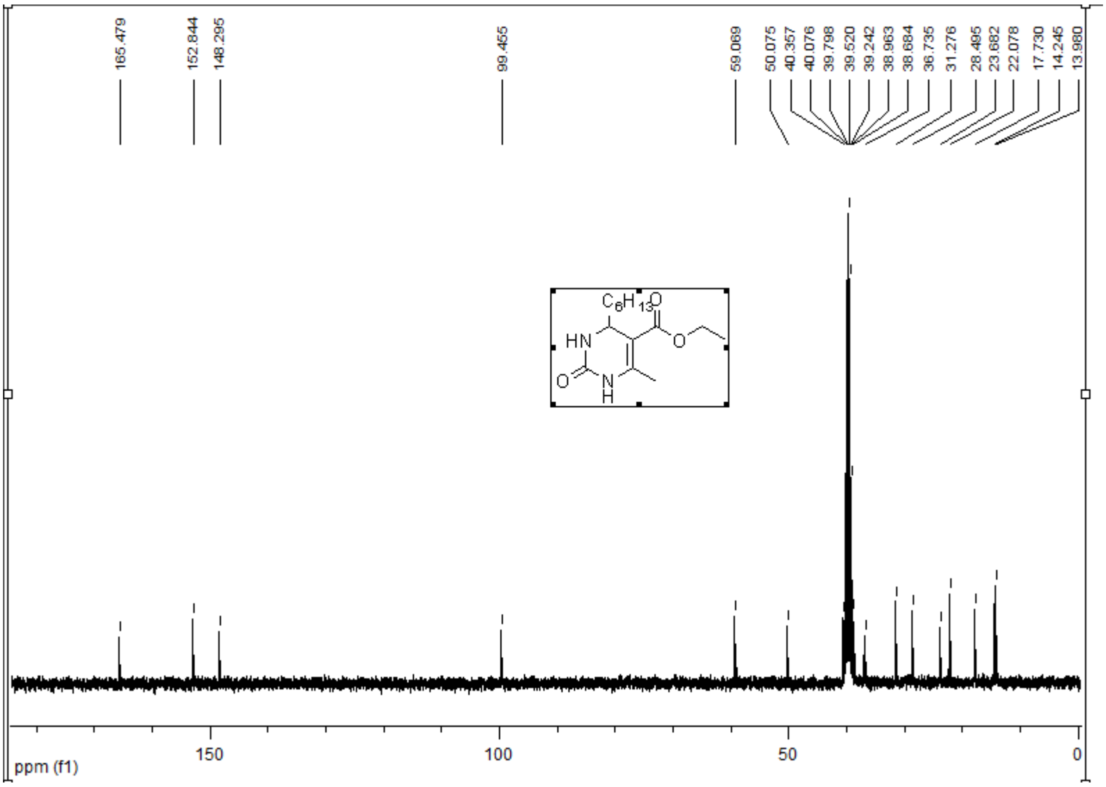

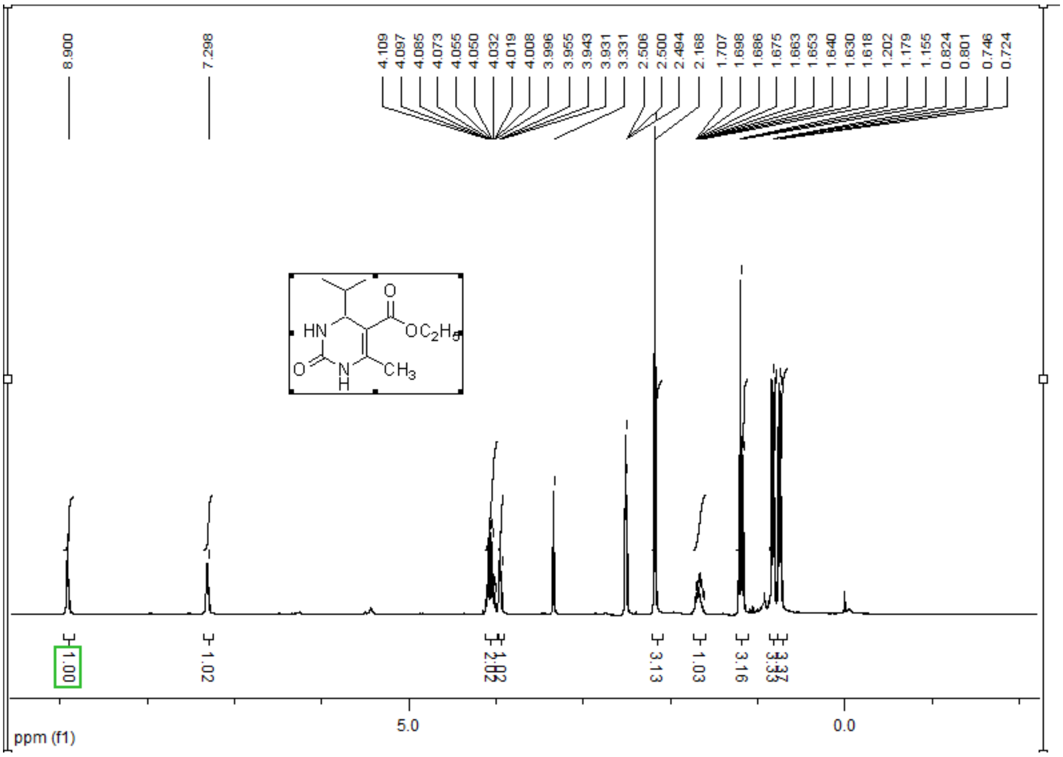
8.900
7.298
4.109
4.097
4.085
4.073
4.055
4.050
4.032
4.019
4.008
3.996
3.955
3.943
3.931
3.331
2.506
2.500
2.494
2.168
1.707
1.698
1.686
1.675
1.663
1.653
1.640
1.630
1.618
1.202
1.179
1.155
0.824
0.801
0.746
0.724
HN
O
OC2H5
O
N
H
CH3
1.00
1.02
3.13
1.03
3.16
5.0
0.0
ppm (f1)

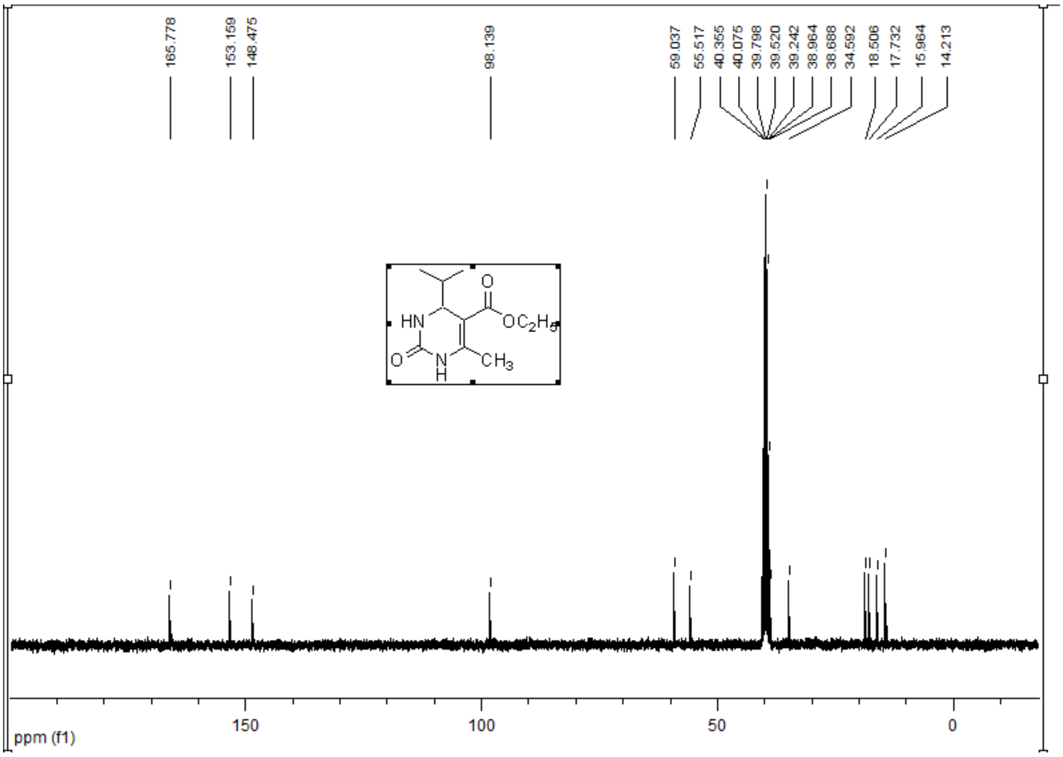
165.778
153.159
148.475
98.139
59.037
55.517
40.355
40.075
39.798
39.520
39.242
38.964
38.688
34.592
18.506
17.732
15.964
14.213
HN
O
OC2H5
O
N
H
CH3
150
100
50
0
ppm (f1)

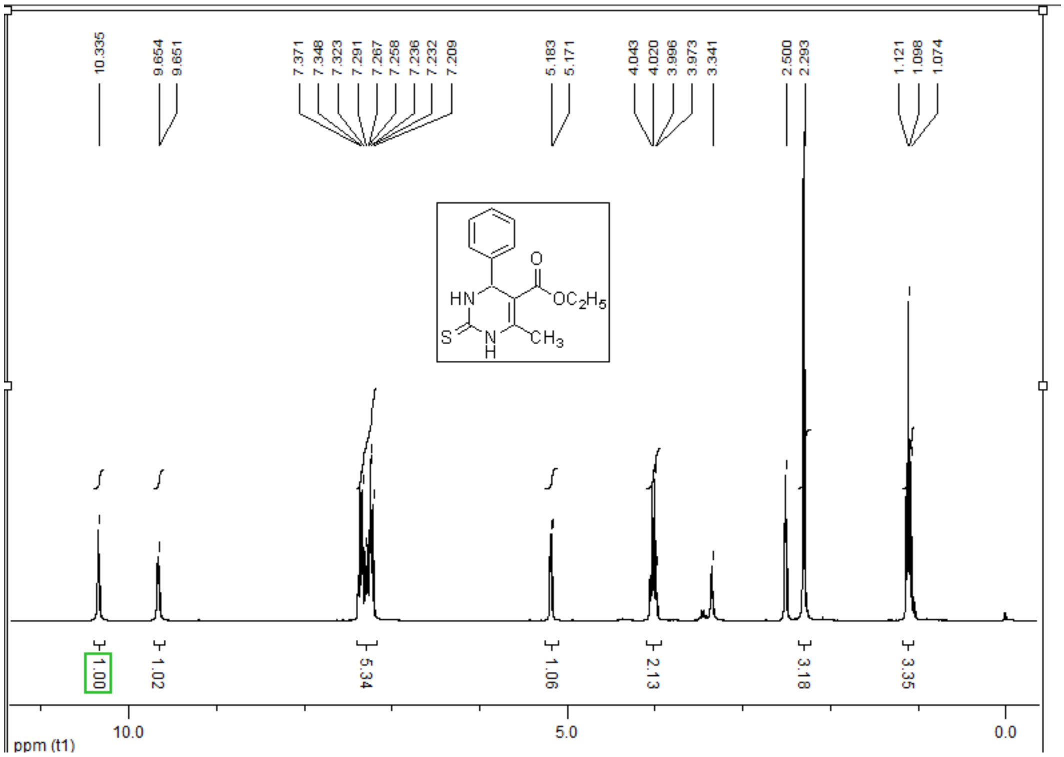
10.335
9.654
9.651
7.371
7.348
7.323
7.291
7.267
7.258
7.236
7.232
7.209
5.183
5.171
4.043
4.020
3.996
3.973
3.341
2.500
2.293
1.121
1.098
1.074
HN
O
OC2H5
S
N
H
CH3
1.00
1.02
5.34
1.06
2.13
3.18
3.35
10.0
5.0
0.0
ppm (t1)

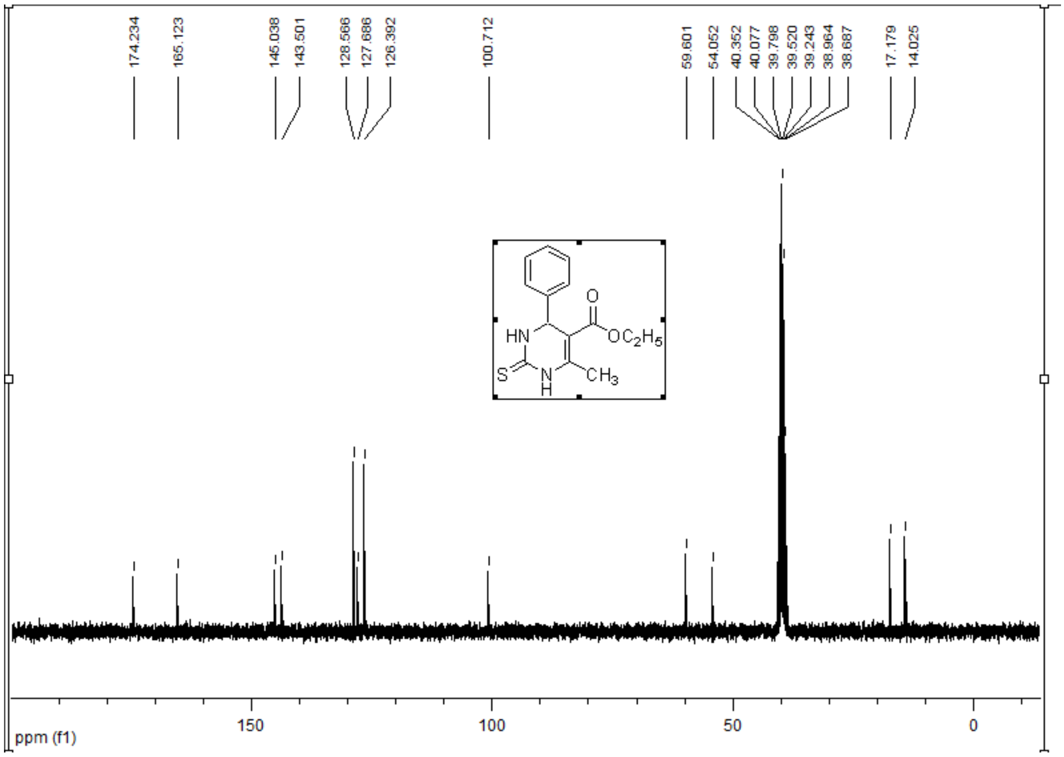
174.234
165.123
145.038
143.501
128.566
127.686
126.392
100.712
59.601
54.052
40.352
40.077
39.798
39.520
39.243
38.964
38.687
17.179
14.025
HN
O
OC2H5
S
N
H
CH3
150
100
50
0
ppm (f1)

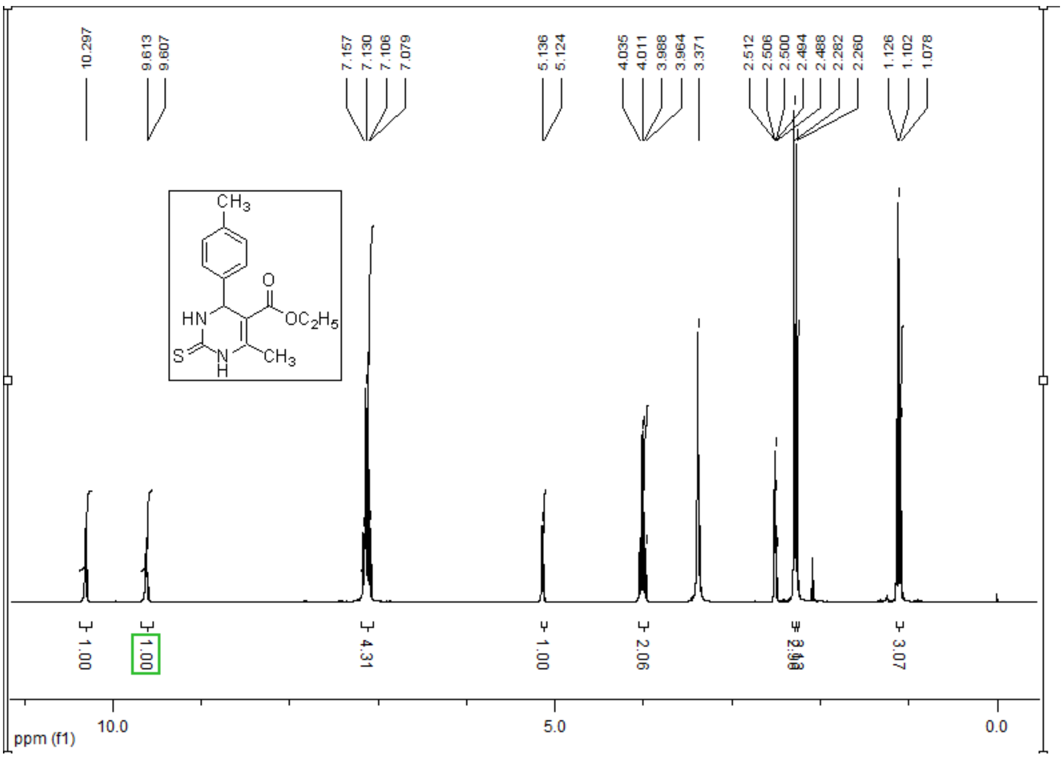
10.297
9.613
9.607
7.157
7.130
7.106
7.079
5.136
5.124
4.035
4.011
3.988
3.964
3.371
2.512
2.506
2.500
2.494
2.488
2.282
2.260
1.126
1.102
1.078
CH3
O
HN
OC2H5
S
N
H
CH3
1.00
1.00
4.31
1.00
2.06
3.07
10.0
5.0
0.0
ppm (f1)

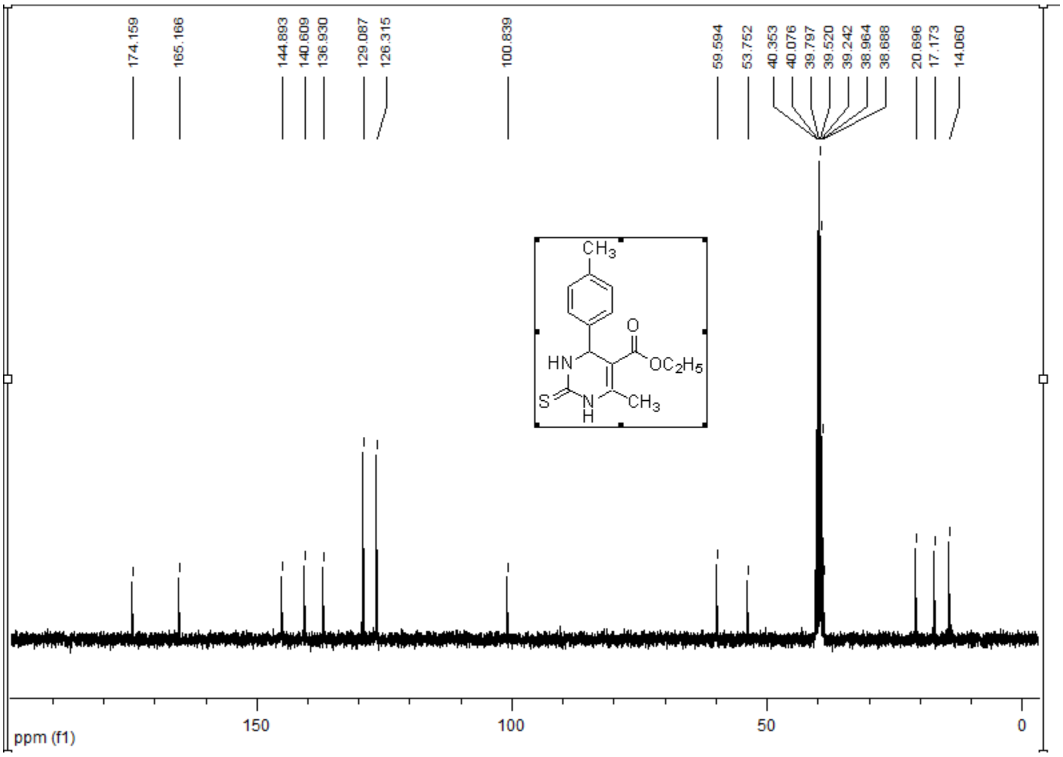
174.159
165.166
144.893
140.609
136.930
129.087
126.315
100.839
59.594
53.752
40.353
40.076
39.797
39.520
39.242
38.964
38.688
20.696
17.173
14.060
CH3
O
HN
OC2H5
S
N
H
CH3
150
100
50
0
ppm (f1)

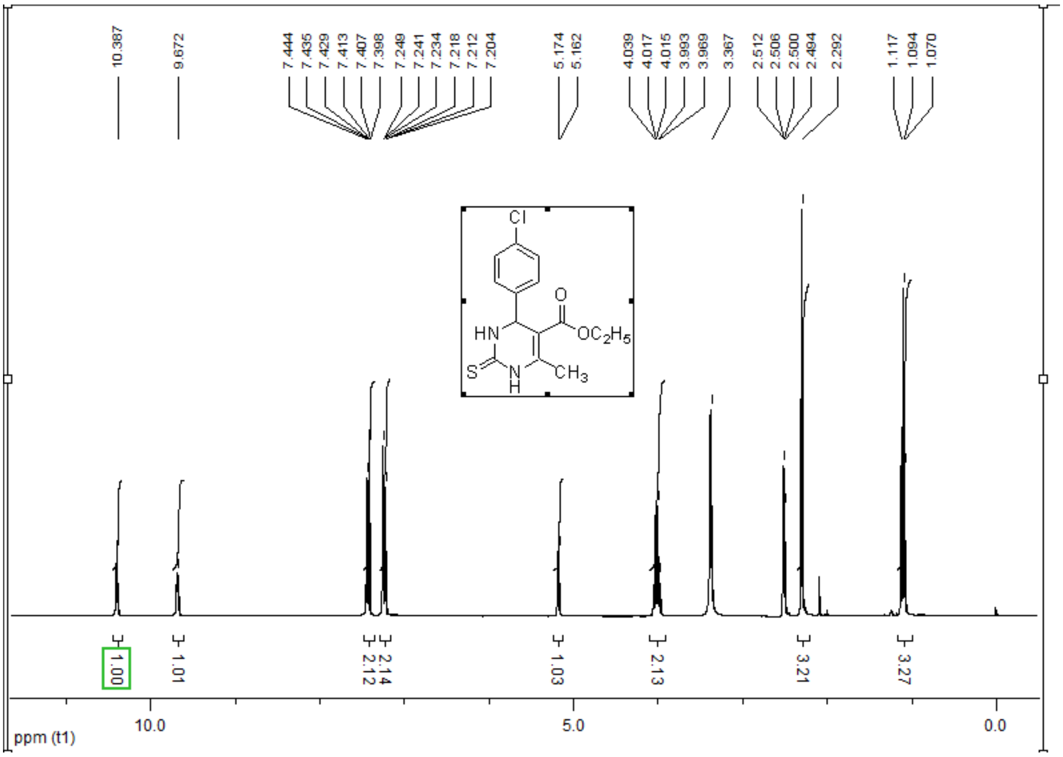
10.387
9.672
7.444
7.435
7.429
7.413
7.407
7.398
7.249
7.241
7.234
7.218
7.212
7.204
5.174
5.162
4.039
4.017
4.015
3.993
3.969
3.367
2.512
2.506
2.500
2.494
2.292
1.117
1.094
1.070
Cl
O
HN
OC_2H_5
S
N
H
CH_3
1.00
1.01
2.12
2.14
1.03
2.13
3.21
3.27
10.0
5.0
0.0
ppm (t1)

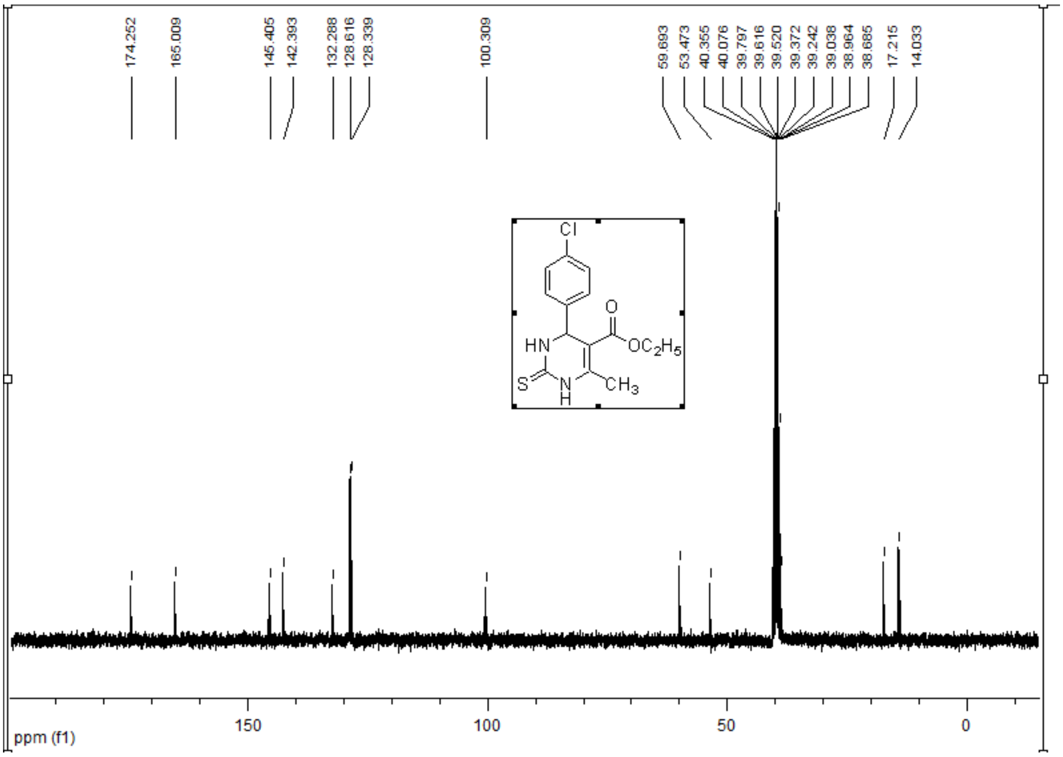
174.252
165.009
145.405
142.393
132.288
128.616
128.339
100.309
59.693
53.473
40.355
40.076
39.797
39.618
39.520
39.372
39.242
39.038
38.964
38.685
17.215
14.033
Cl
O
HN
OC_2H_5
S
N
H
CH_3
150
100
50
0
ppm (f1)

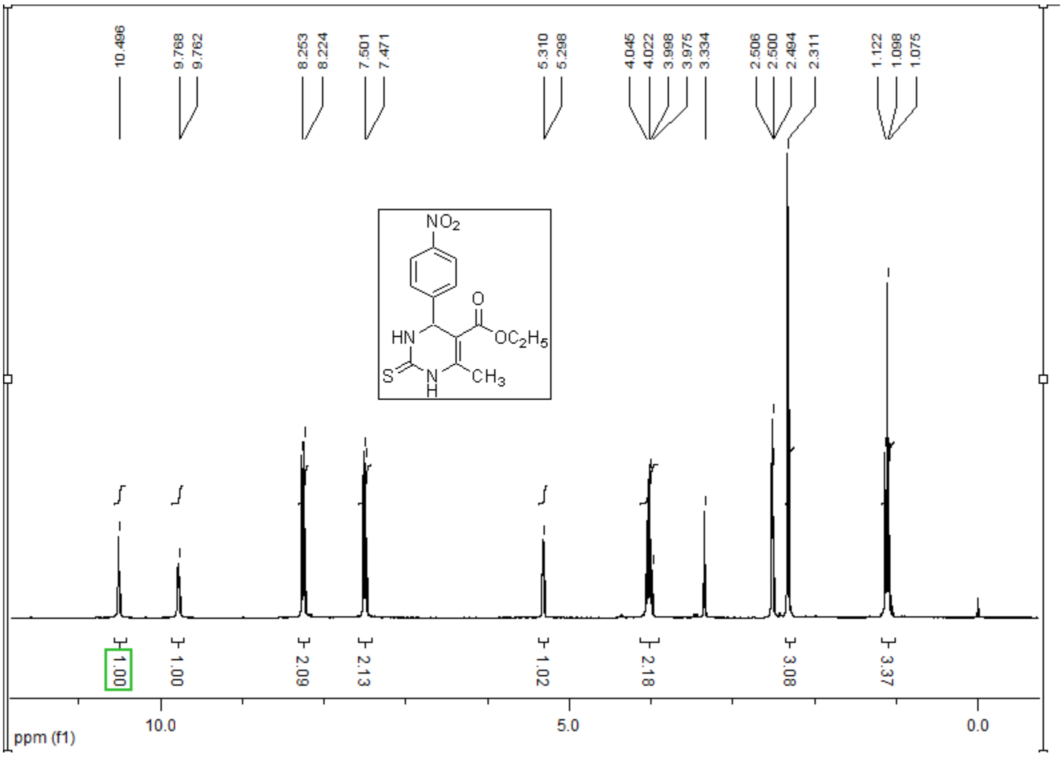
10.496
9.768
9.762
8.253
8.224
7.501
7.471
5.310
5.298
4.045
4.022
3.998
3.975
3.334
2.506
2.500
2.494
2.311
1.122
1.098
1.075
1.00
1.00
2.09
2.13
1.02
2.18
3.08
3.37
10.0
5.0
0.0
ppm (f1)

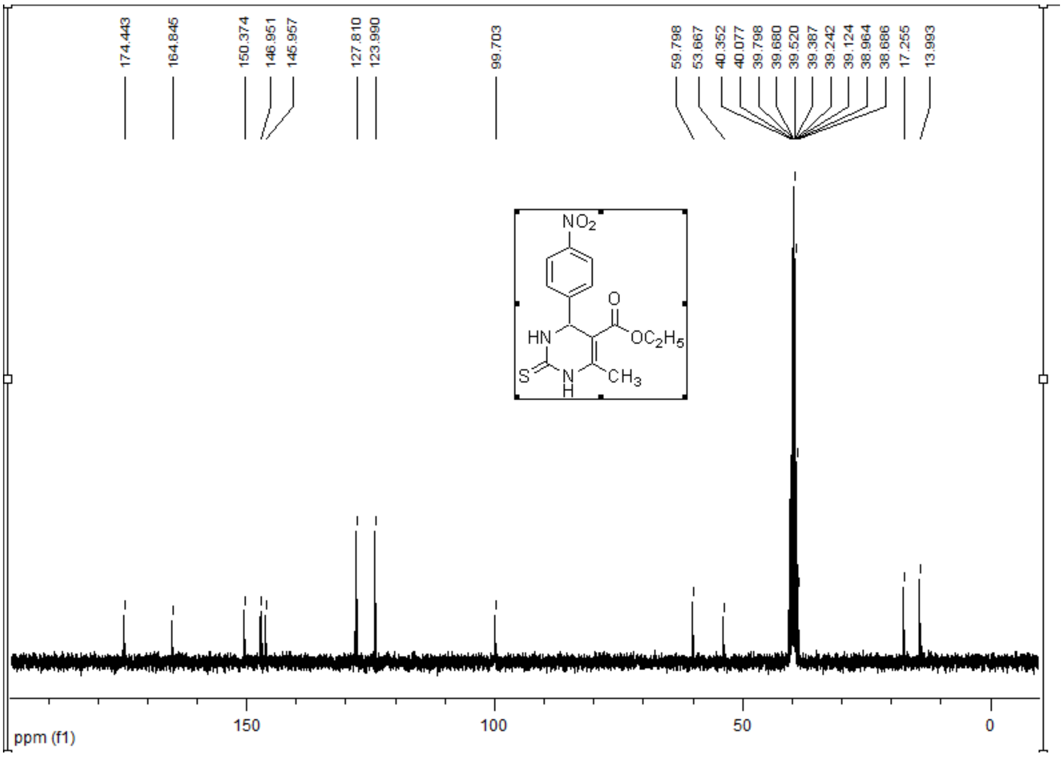
174.443
164.845
150.374
146.951
145.957
127.810
123.990
99.703
59.798
53.667
40.352
40.077
39.798
39.680
39.520
39.387
39.242
39.124
38.964
38.686
17.255
13.993
150
100
50
0
ppm (f1)

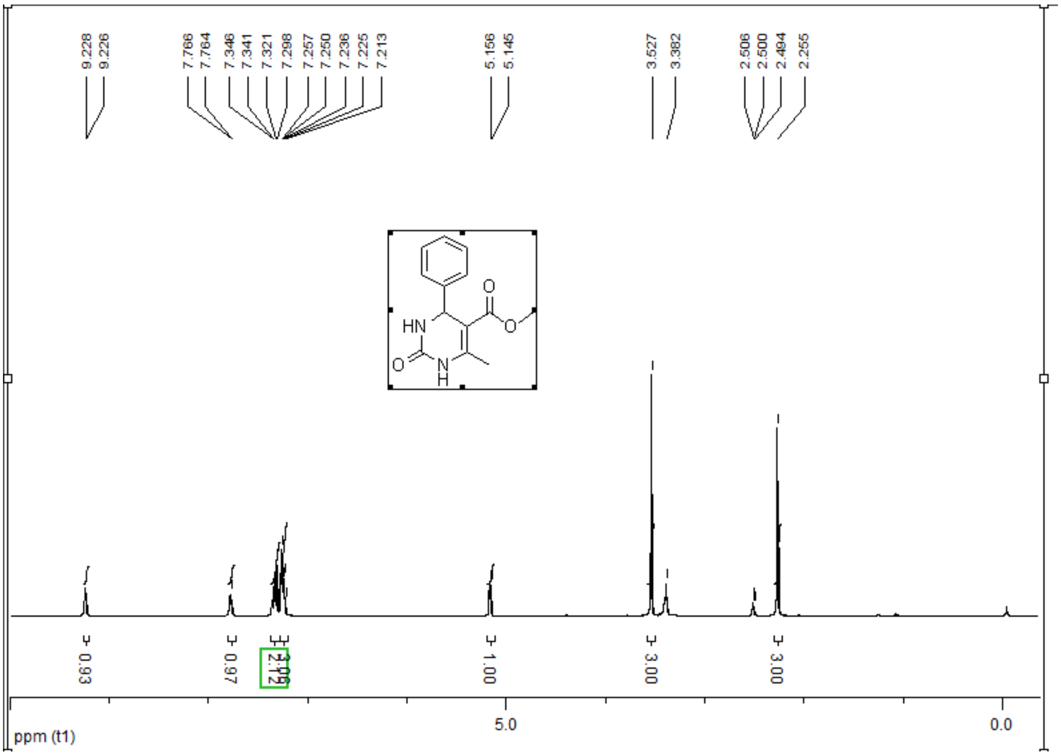

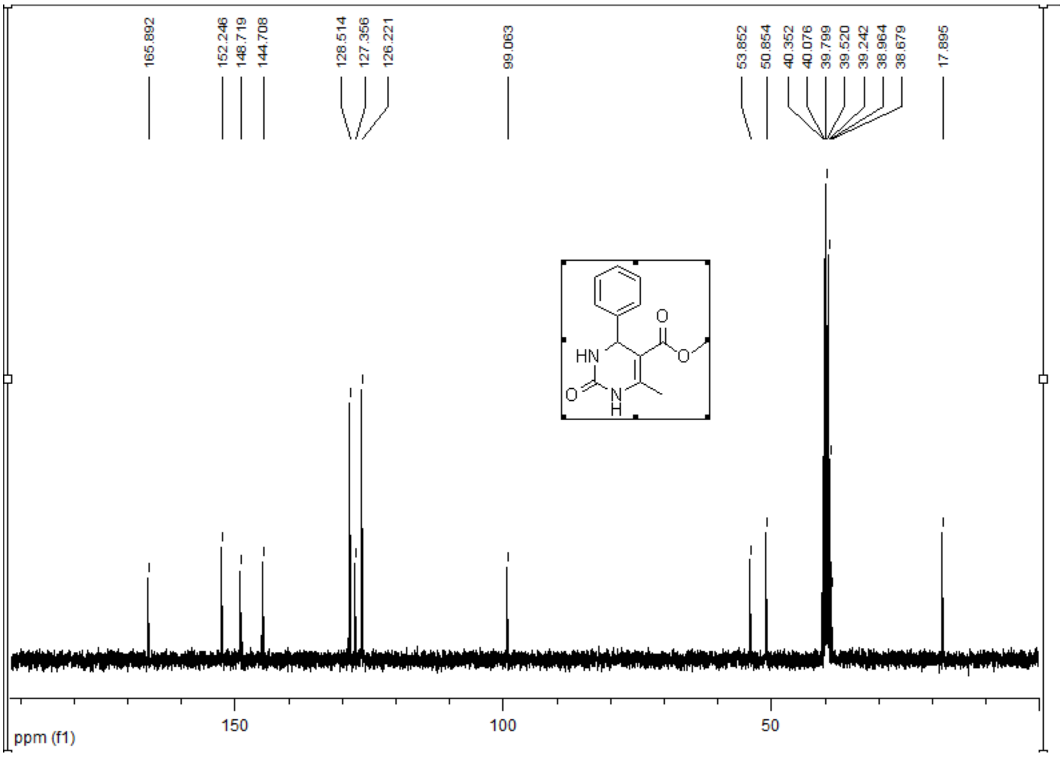

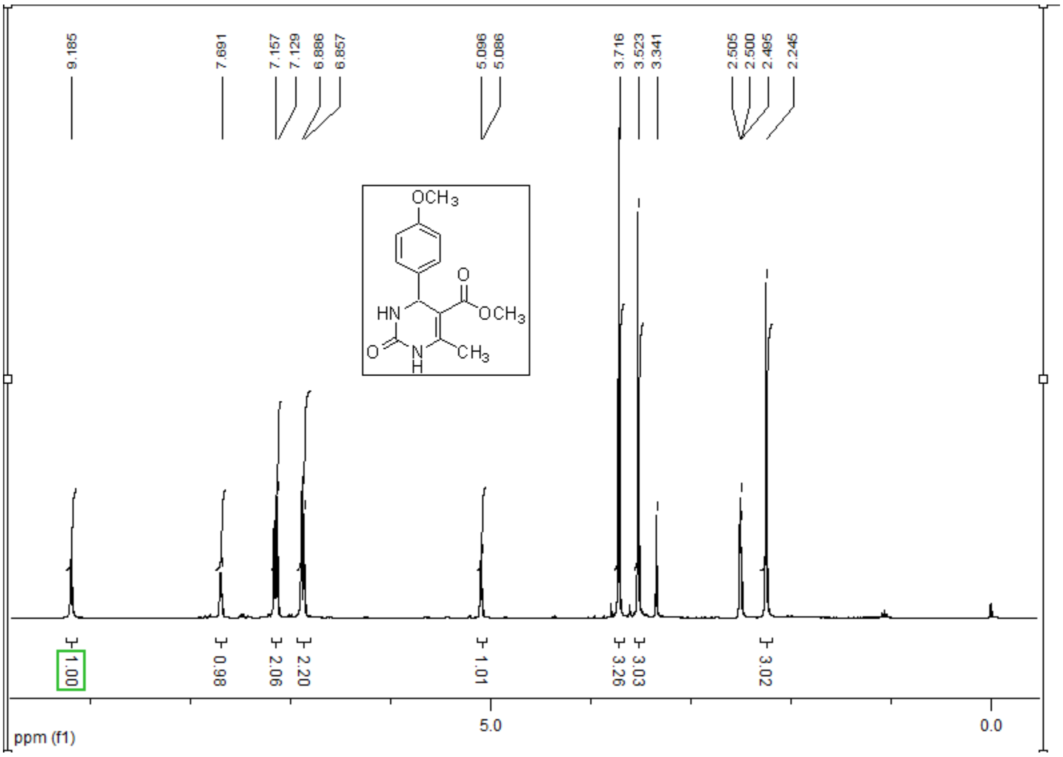
9.185
7.691
7.157
7.129
6.886
6.857
5.096
5.086
3.716
3.523
3.341
2.505
2.500
2.495
2.245
OCH3
O
HN
OCH3
O
N
H
CH3
1.00
0.98
2.06
2.20
1.01
3.26
3.03
3.02
5.0
0.0
ppm (f1)

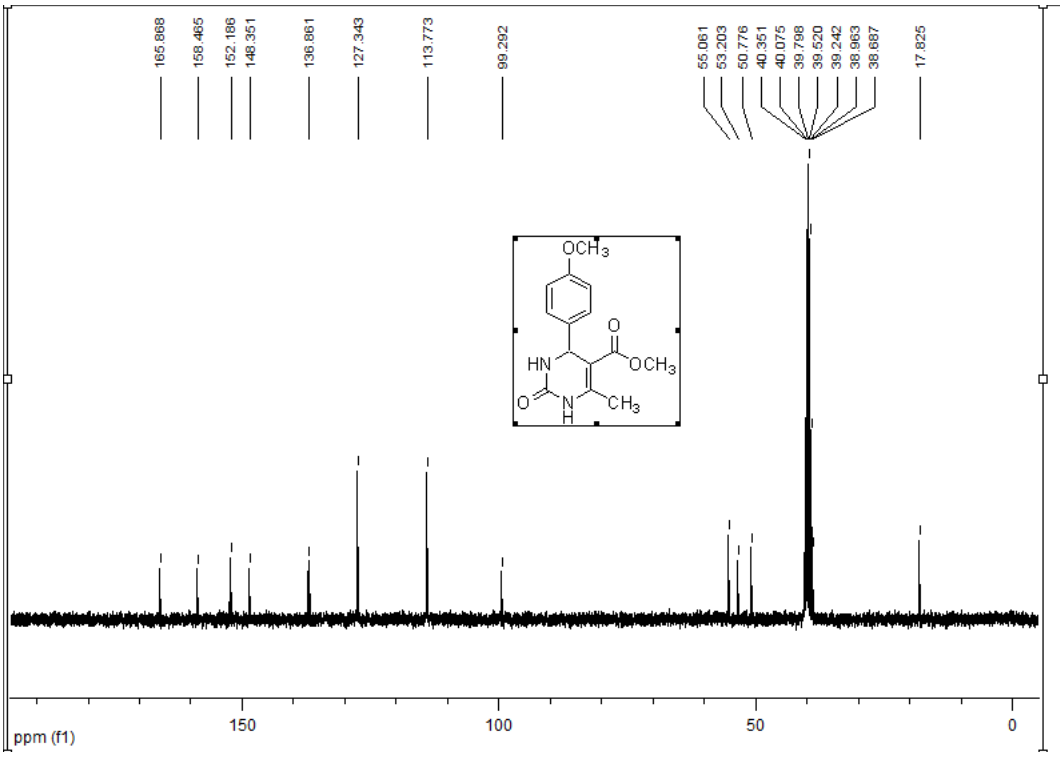
165.868
158.465
152.186
148.351
136.861
127.343
113.773
99.292
55.061
53.203
50.776
40.351
40.075
39.798
39.520
39.242
38.963
38.687
17.825
OCH3
O
HN
OCH3
O
N
H
CH3
150
100
50
0
ppm (f1)

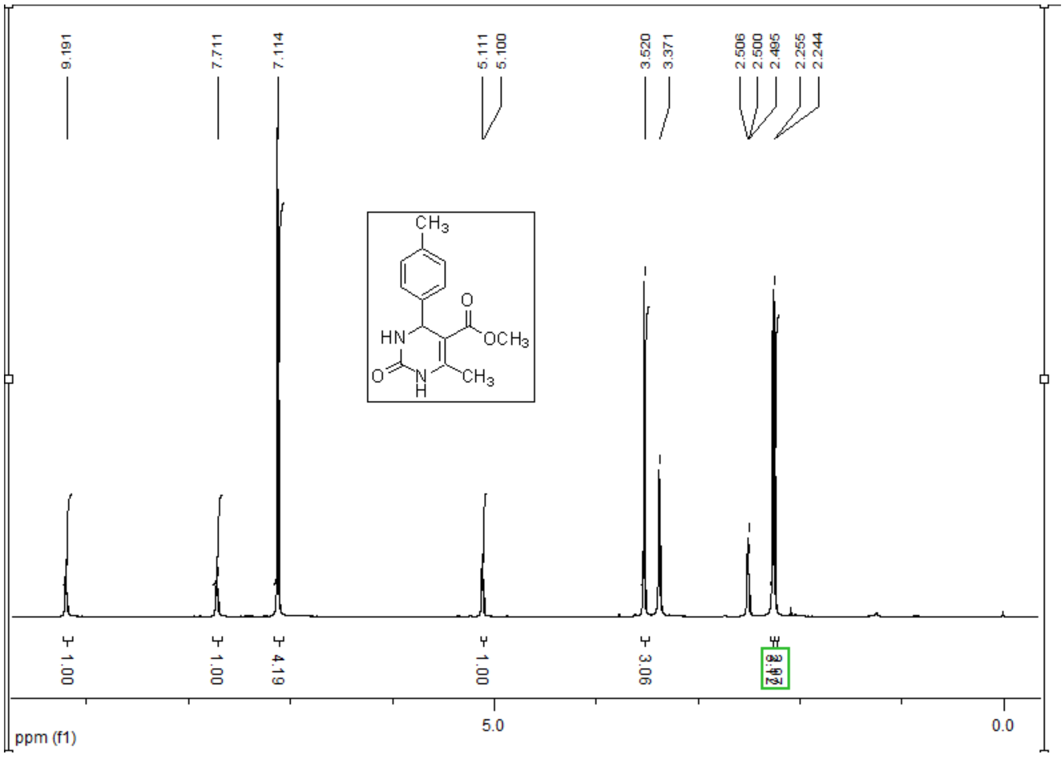
9.191
7.711
7.114
5.111
5.100
3.520
3.371
2.506
2.500
2.495
2.255
2.244
1.00
1.00
4.19
1.00
3.06
5.0
0.0
ppm (f1)

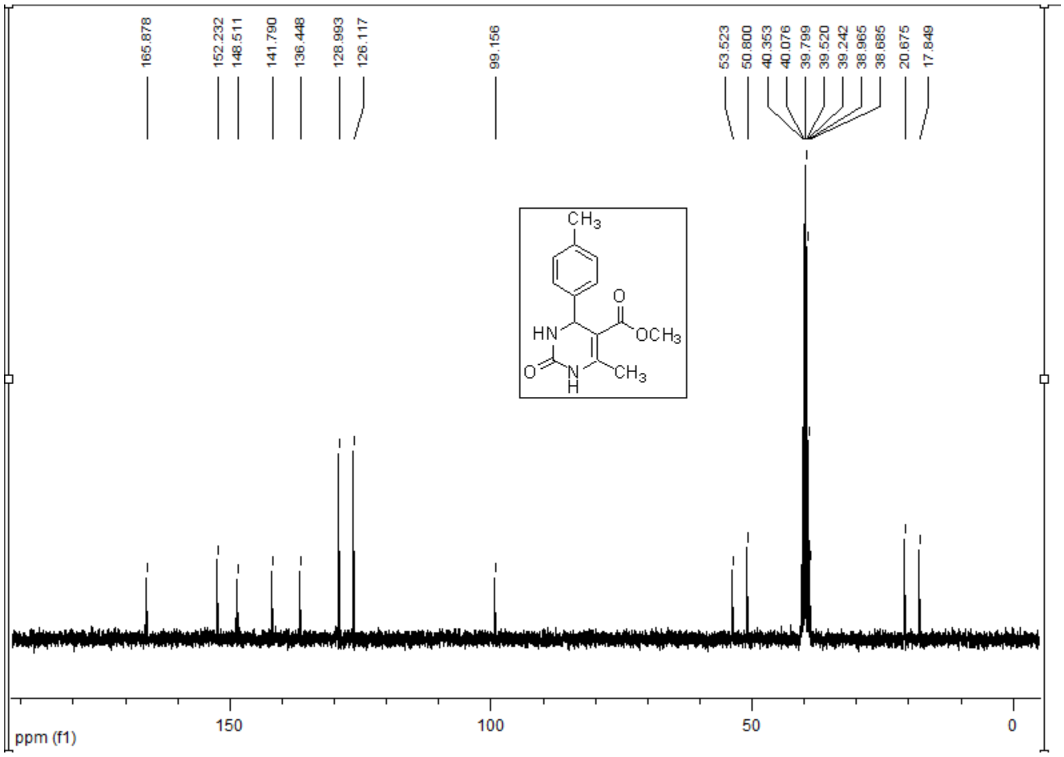
165.878
152.232
148.511
141.790
136.448
128.993
126.117
99.156
53.523
50.800
40.353
40.076
39.799
39.520
39.242
38.965
38.685
20.675
17.849
150
100
50
0
ppm (f1)

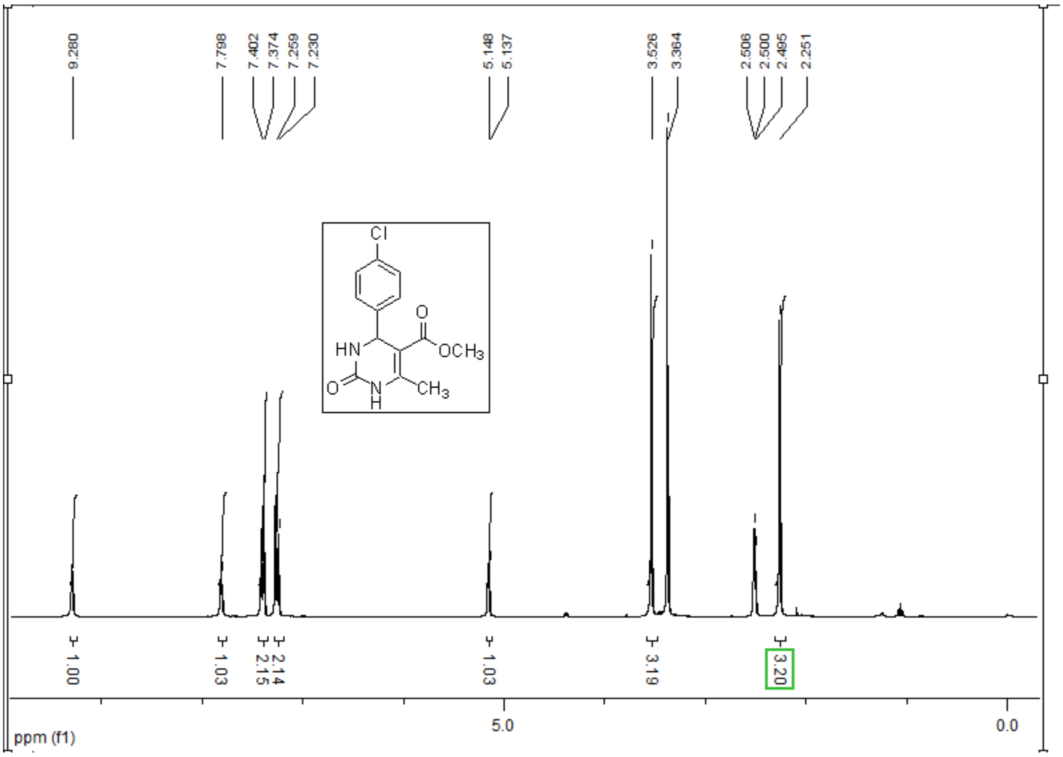
9.280
7.798
7.402
7.374
7.259
7.230
5.148
5.137
3.526
3.364
2.506
2.500
2.495
2.251
Cl
O
HN
OCH3
O
N
H
CH3
1.00
1.03
2.15
2.14
1.03
3.19
3.20
5.0
0.0
ppm (f1)

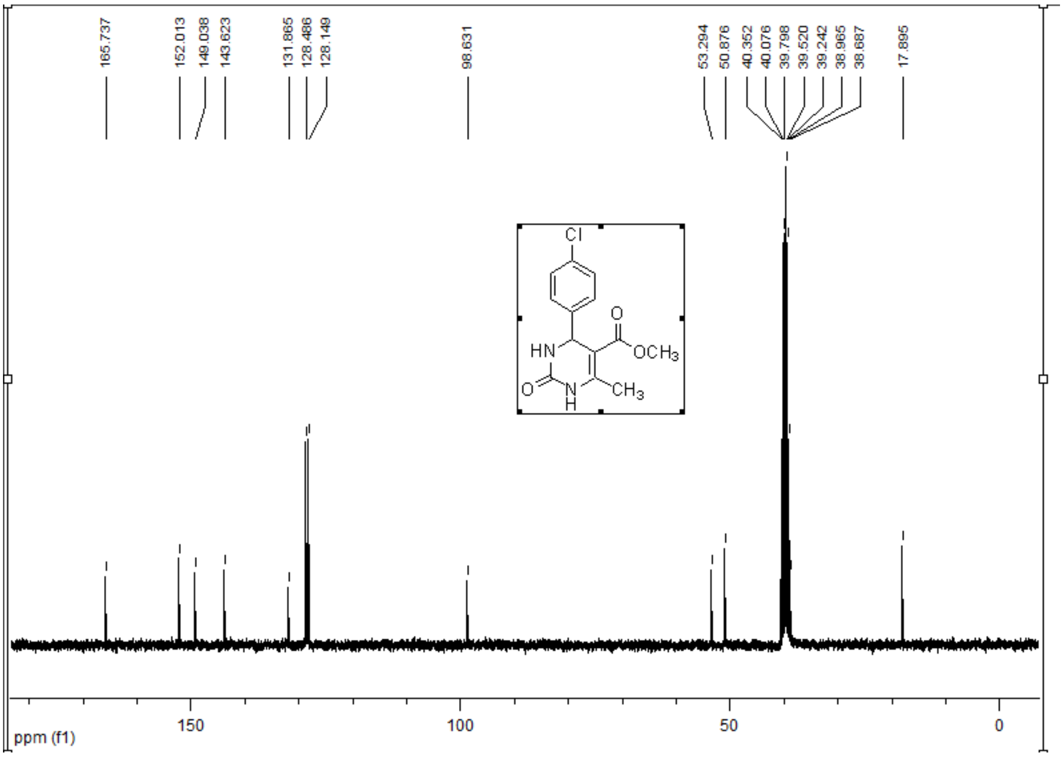
165.737
152.013
149.038
143.623
131.865
128.486
128.149
98.631
53.294
50.876
40.352
40.076
39.798
39.520
39.242
38.965
38.687
17.895
Cl
O
HN
OCH3
O
N
H
CH3
150
100
50
0
ppm (f1)

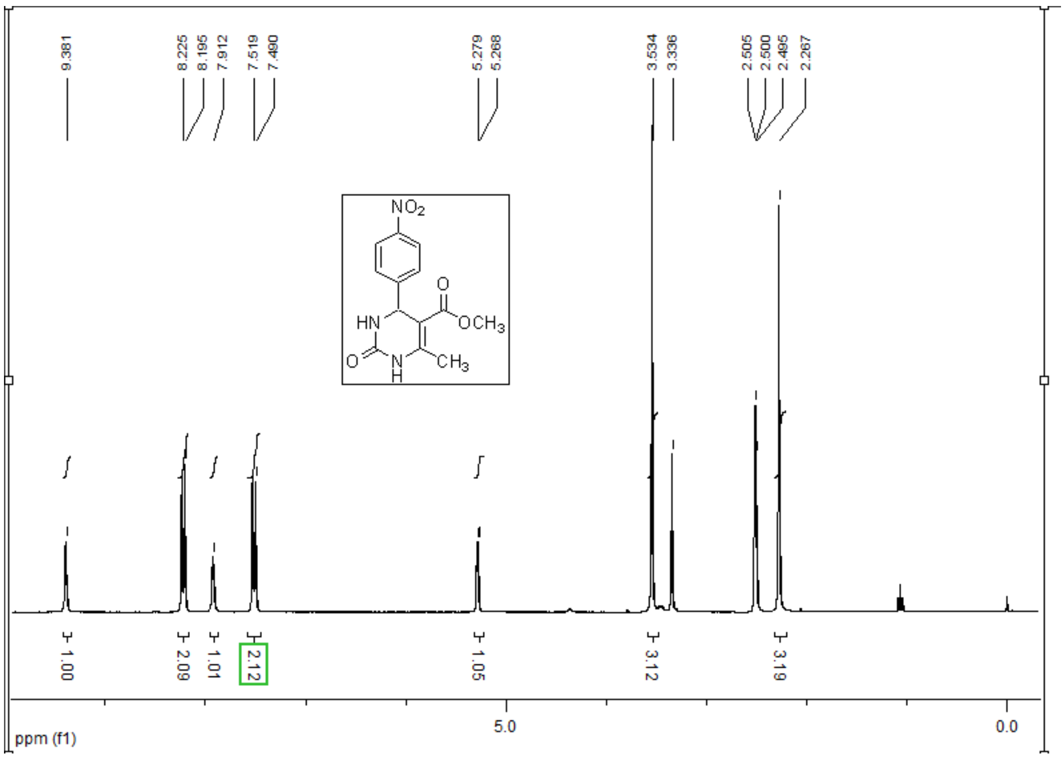
9.381
8.225
8.195
7.912
7.519
7.490
5.279
5.268
3.534
3.336
2.505
2.500
2.495
2.267
NO_2
HN
O
N
H
O
OCH_3
CH_3
1.00
2.09
1.01
2.12
1.05
3.12
3.19
5.0
0.0
ppm (f1)

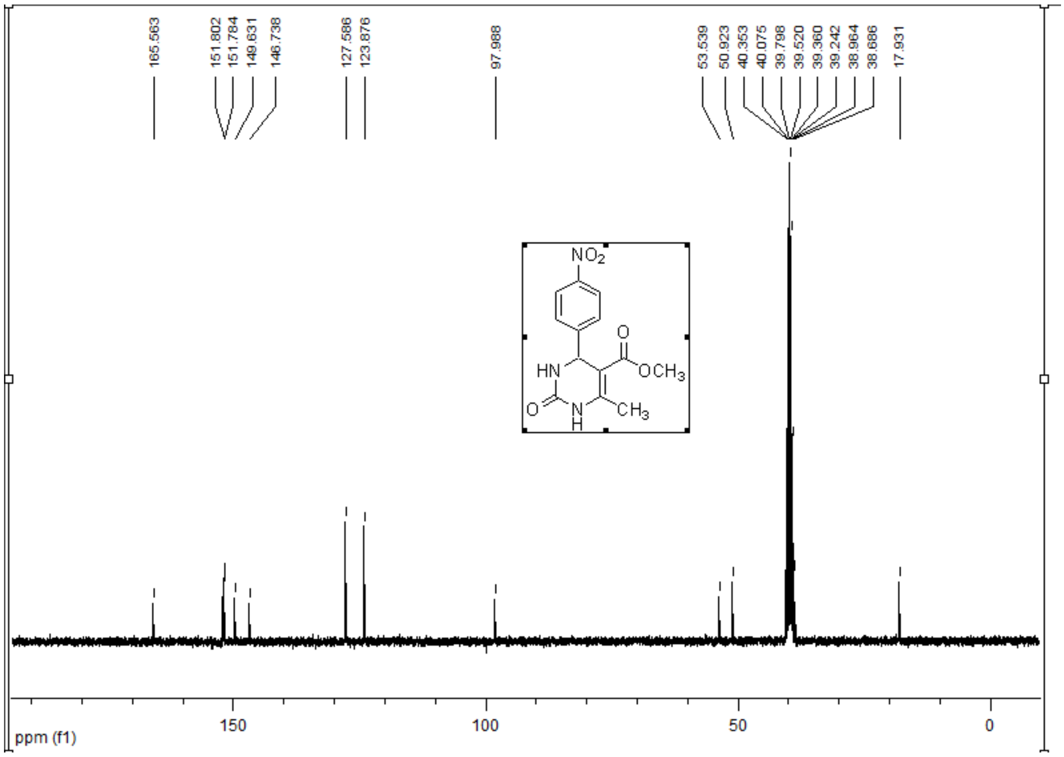
165.563
151.802
151.784
149.631
146.738
127.586
123.876
97.988
53.539
50.923
40.353
40.075
39.798
39.520
39.360
39.242
38.964
38.686
17.931
NO_2
HN
O
N
H
O
OCH_3
CH_3
150
100
50
0
ppm (f1)

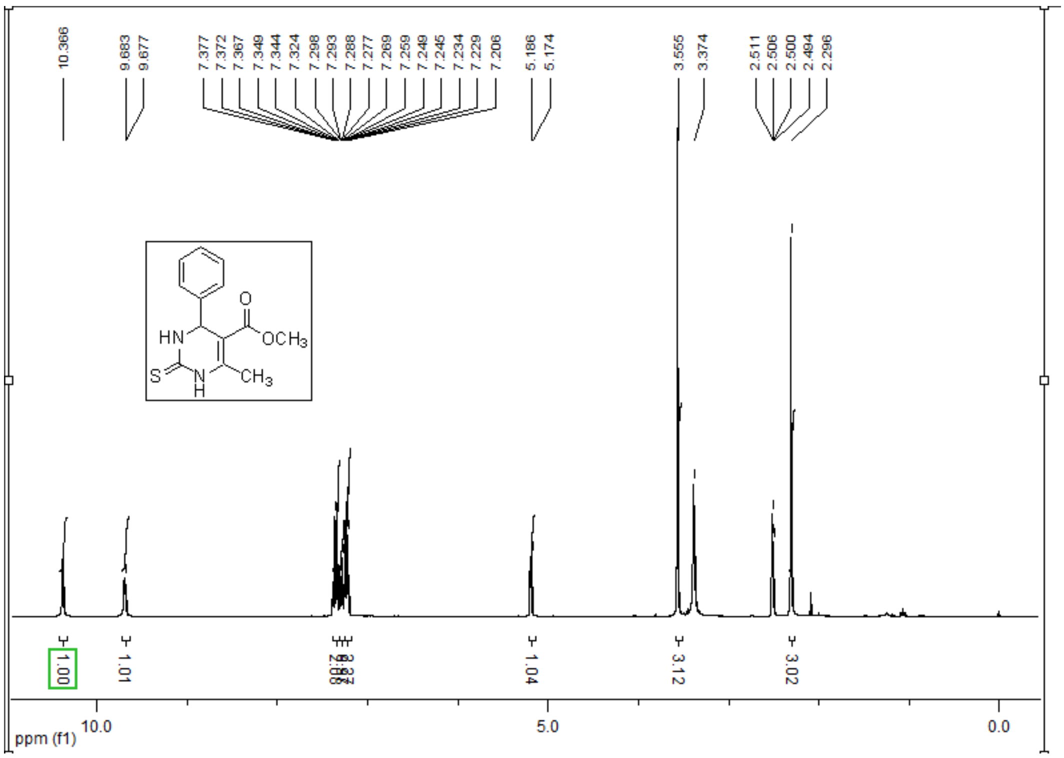
10.366
9.683
9.677
7.377
7.372
7.367
7.349
7.344
7.324
7.298
7.293
7.288
7.277
7.269
7.259
7.249
7.245
7.234
7.229
7.206
5.186
5.174
3.555
3.374
2.511
2.506
2.500
2.494
2.296
HN
S
N
H
O
OCH3
CH3
1.00
1.01
1.04
3.12
3.02
ppm (f1)
10.0
5.0
0.0

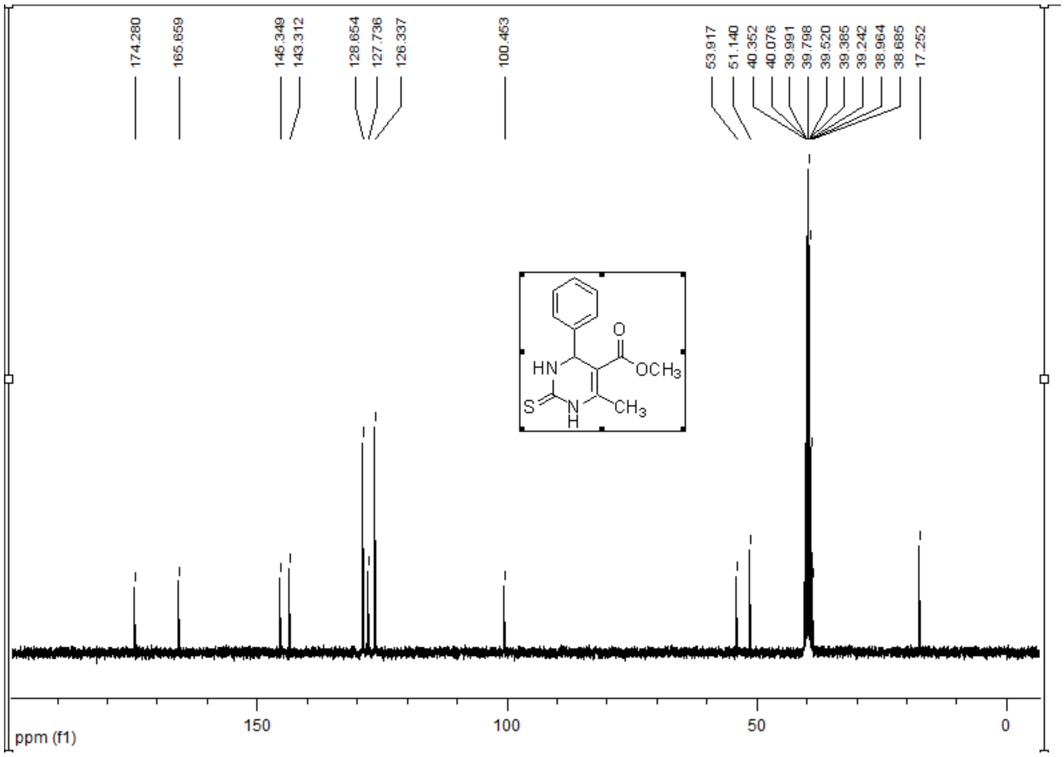
174.280
165.659
145.349
143.312
128.654
127.736
126.337
100.453
53.917
51.140
40.352
40.076
39.991
39.798
39.520
39.385
39.242
38.964
38.685
17.252
HN
S
N
H
O
OCH3
CH3
ppm (f1)
150
100
50
0

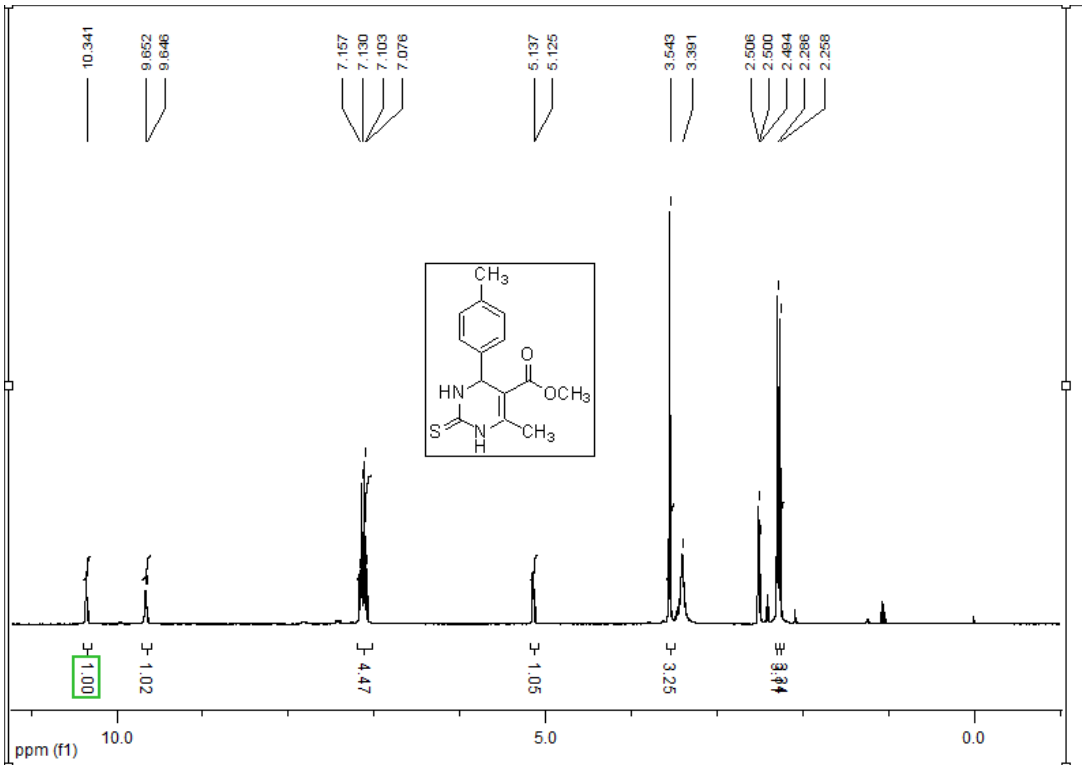
10.341
9.652
9.646
7.157
7.130
7.103
7.076
5.137
5.125
3.543
3.391
2.506
2.500
2.494
2.286
2.258
CH3
O
HN
OCH3
S
N
H
CH3
1.00
1.02
4.47
1.05
3.25
10.0
5.0
0.0
ppm (f1)

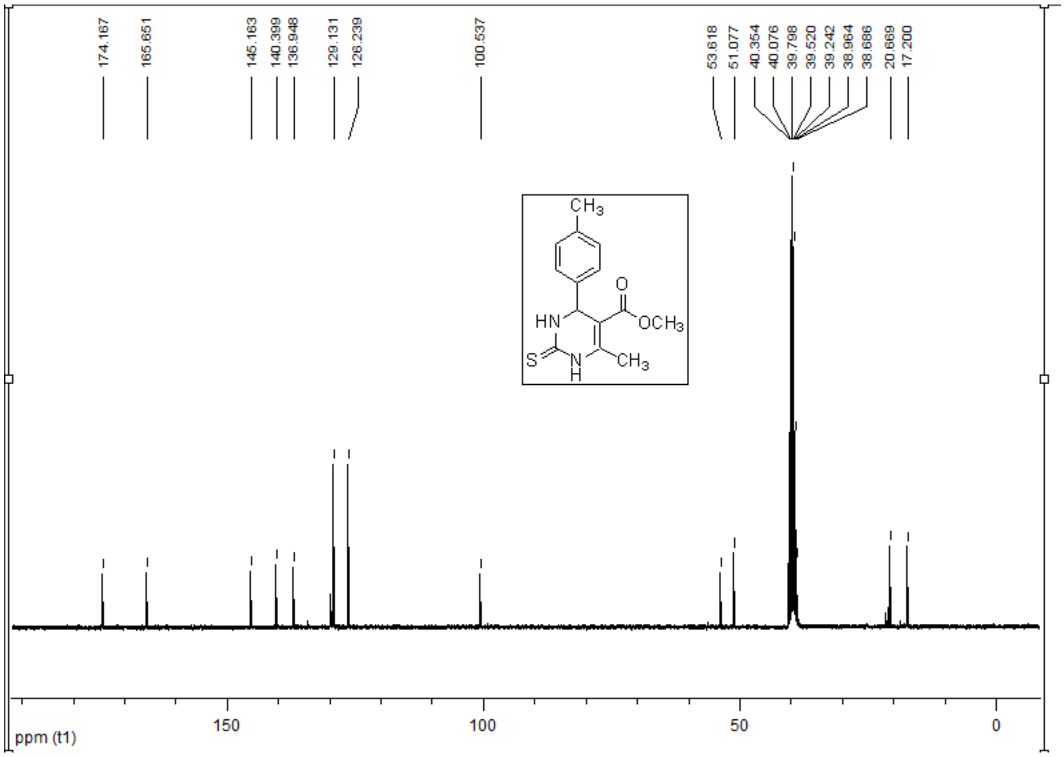
174.167
165.651
145.163
140.399
136.948
129.131
126.239
100.537
53.618
51.077
40.354
40.076
39.798
39.520
39.242
38.964
38.686
20.669
17.200
CH3
O
HN
OCH3
S
N
H
CH3
150
100
50
0
ppm (t1)

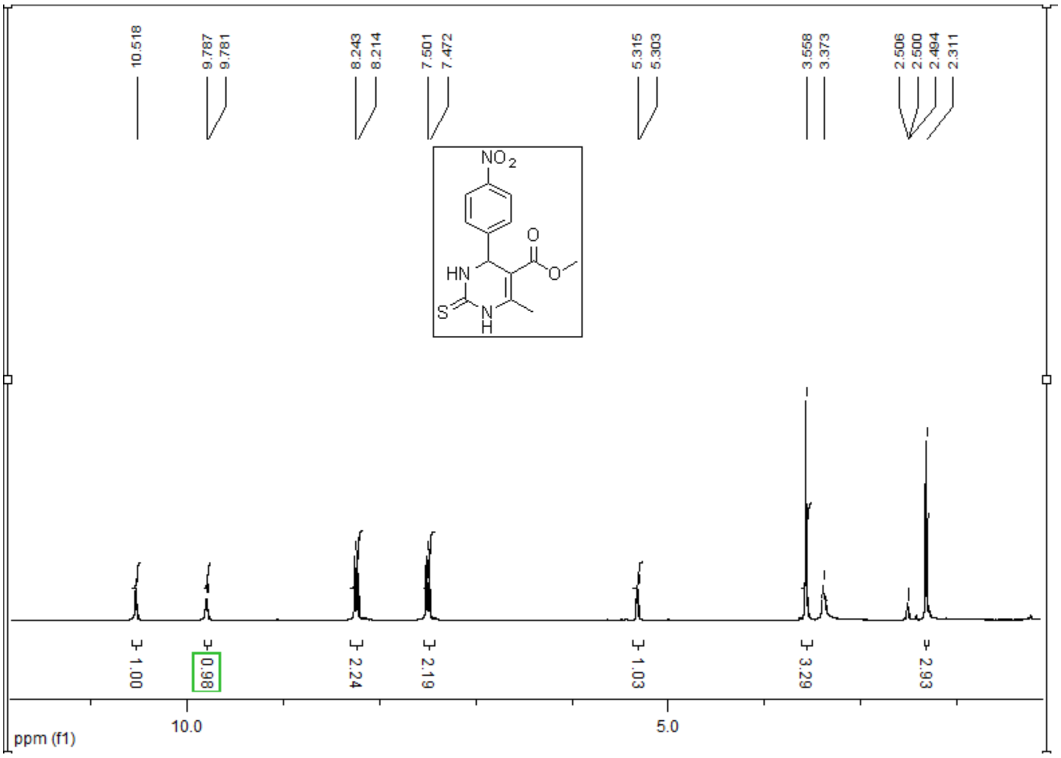
10.518
9.787
9.781
8.243
8.214
7.501
7.472
5.315
5.303
3.558
3.373
2.506
2.500
2.494
2.311
1.00
0.98
2.24
2.19
1.03
3.29
2.93
10.0
5.0
ppm (f1)

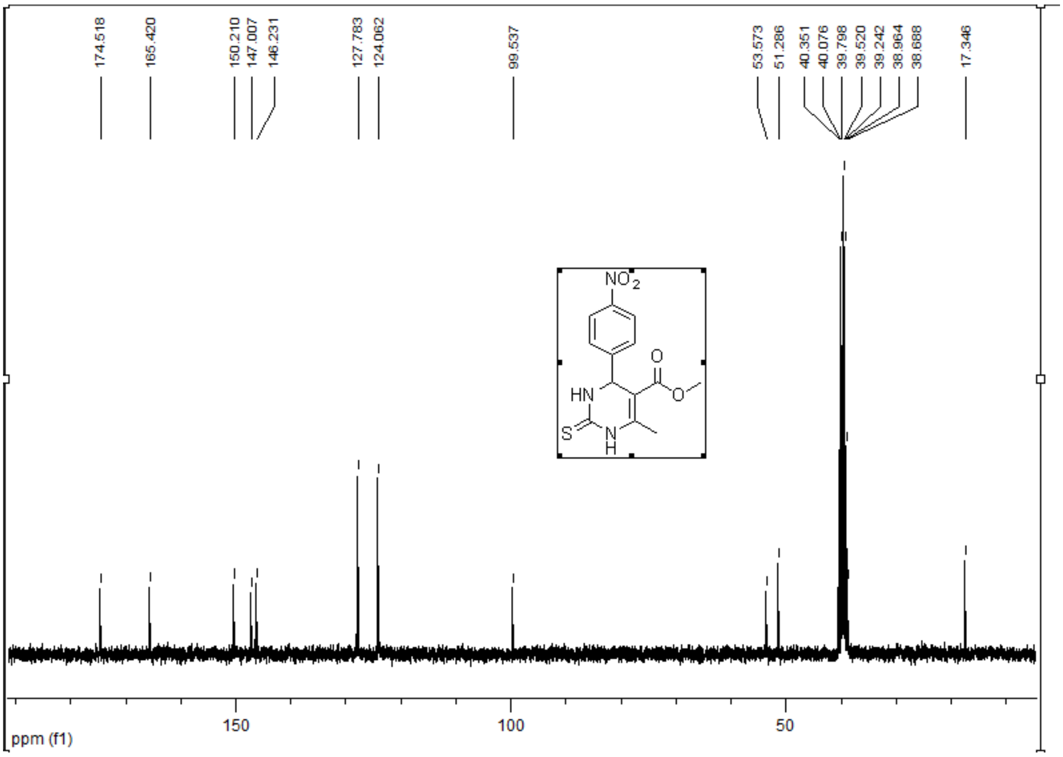
174.518
165.420
150.210
147.007
146.231
127.783
124.062
99.537
53.573
51.286
40.351
40.076
39.798
39.520
39.242
38.964
38.688
17.346
150
100
50
ppm (f1)

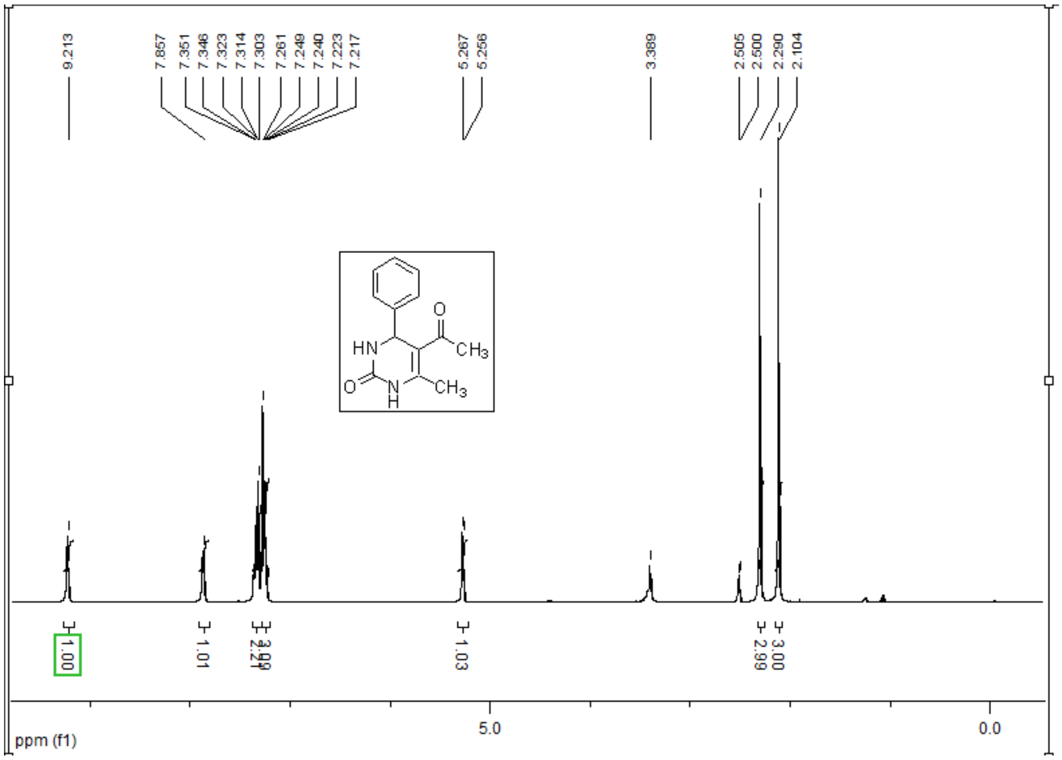

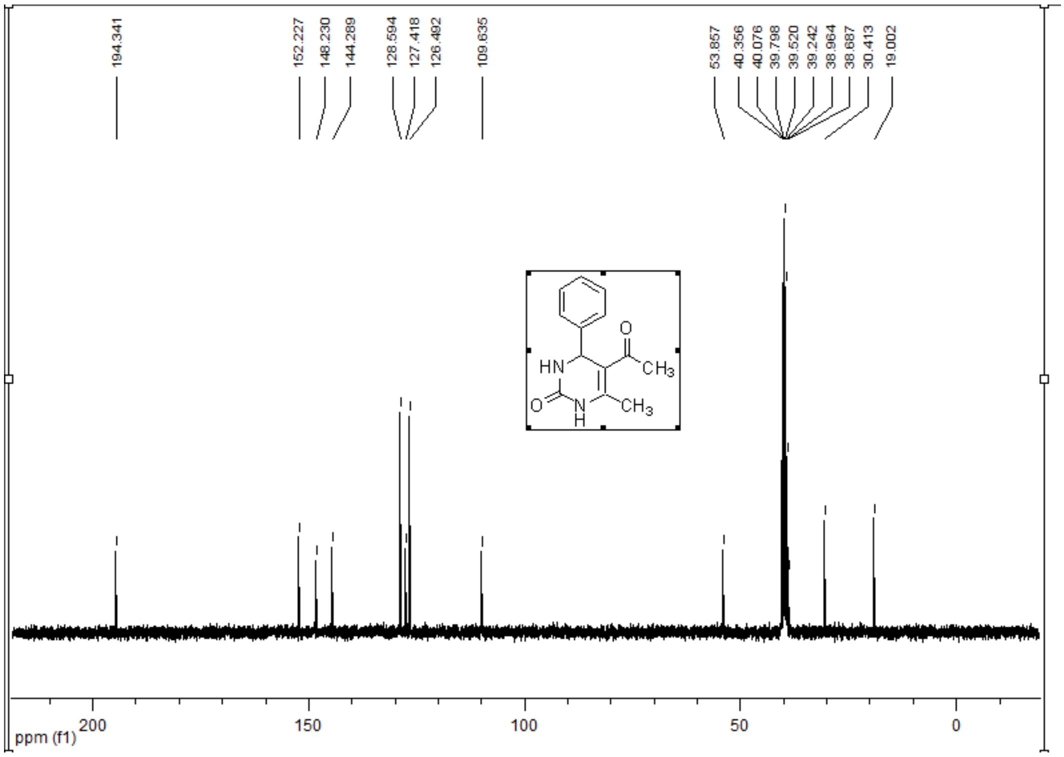

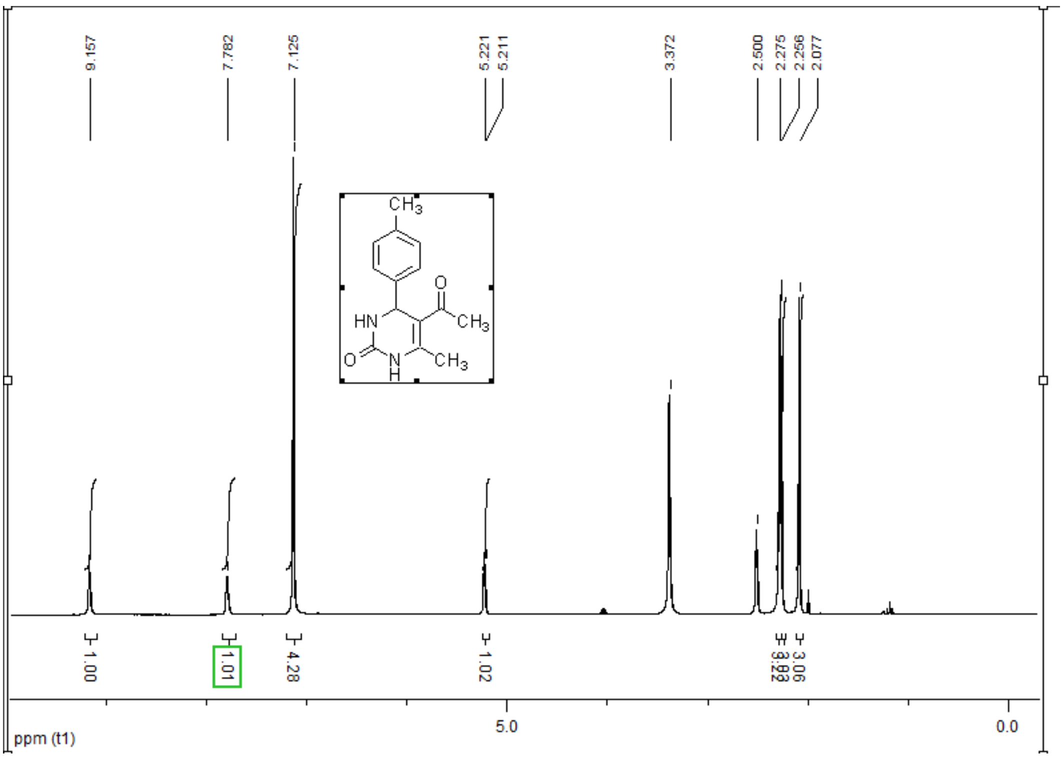
9.157
7.782
7.125
5.221
5.211
3.372
2.500
2.275
2.256
2.077
1.00
1.01
4.28
1.02
3.06
5.0
0.0
ppm (t1)
CH3
O
HN
CH3
O
N
H
CH3

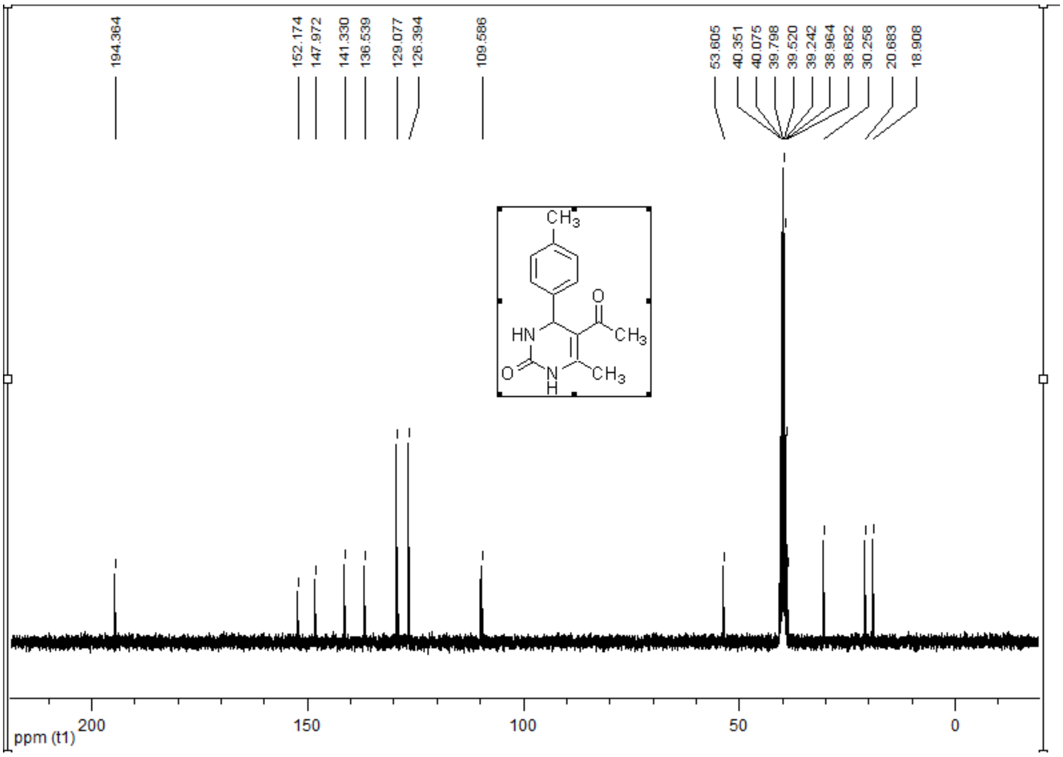
194.364
152.174
147.972
141.330
136.539
129.077
126.394
109.586
53.605
40.351
40.075
39.798
39.520
39.242
38.964
38.682
30.258
20.683
18.908
200
150
100
50
0
ppm (t1)
CH3
O
HN
CH3
O
N
H
CH3

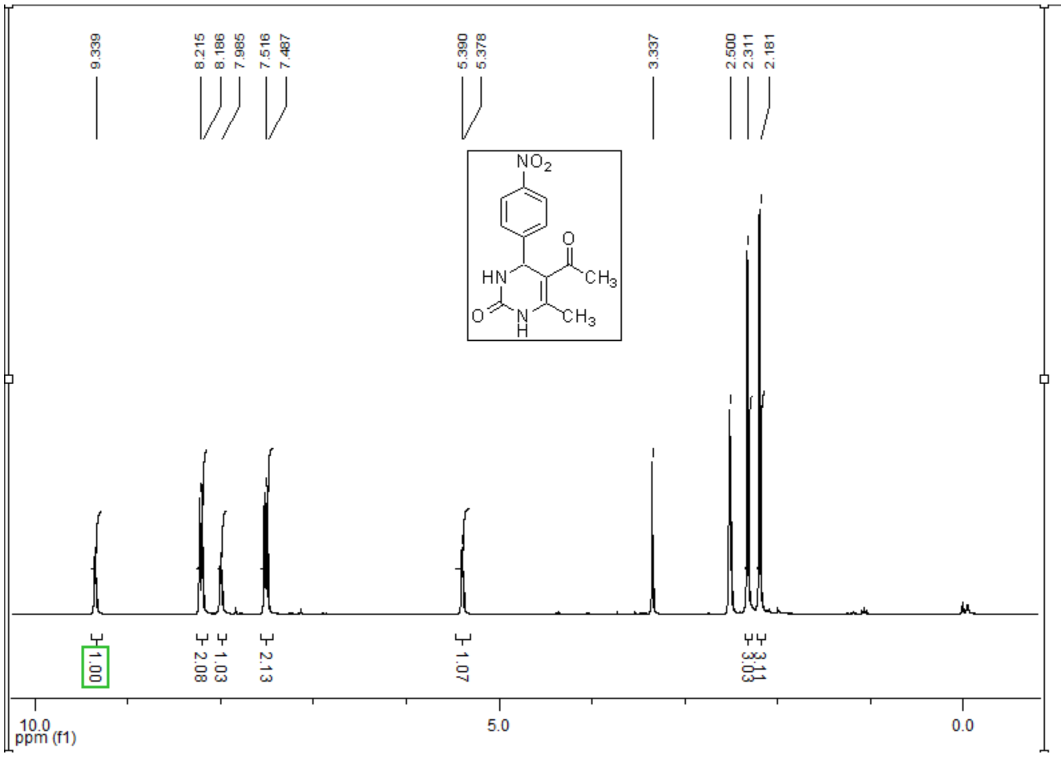
9.339
8.215
8.186
7.985
7.516
7.487
5.390
5.378
3.337
2.500
2.311
2.181
NO2
O
CH3
HN
O
N
H
CH3
1.00
2.08
1.03
2.13
1.07
3.03
3.11
10.0
ppm (f1)
5.0
0.0

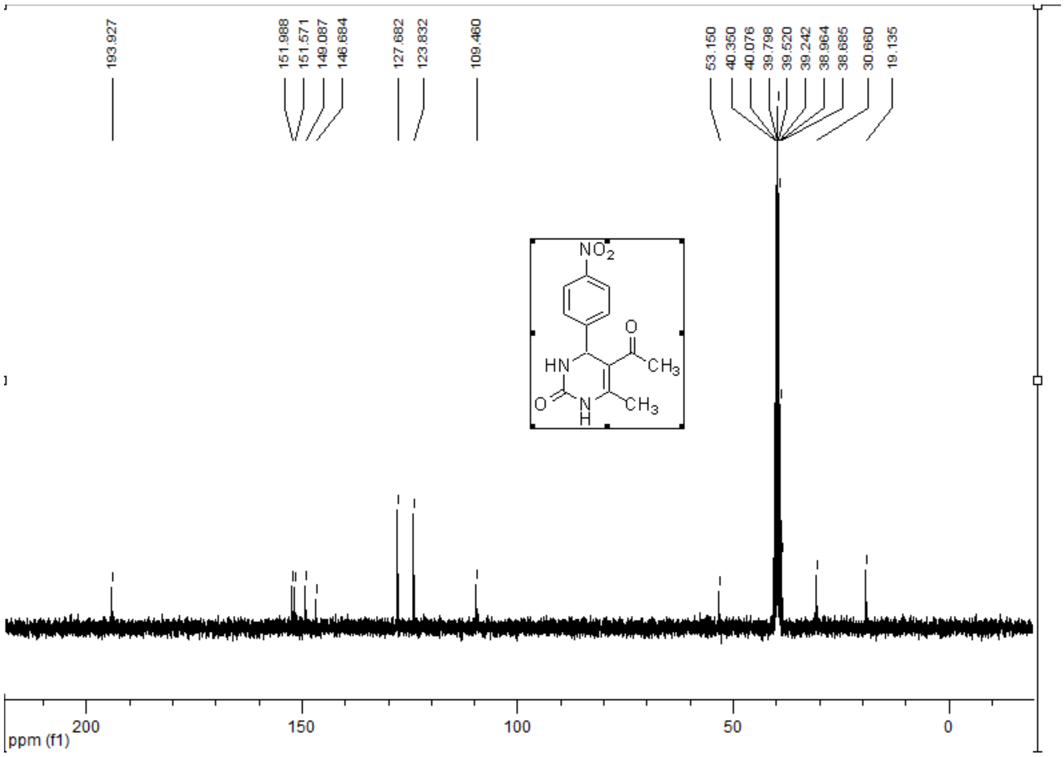
193.927
151.988
151.571
149.087
146.684
127.682
123.832
109.460
53.150
40.350
40.076
39.798
39.520
39.242
38.964
38.685
30.660
19.135
NO2
O
CH3
HN
O
N
H
CH3
200
150
100
50
0
ppm (f1)

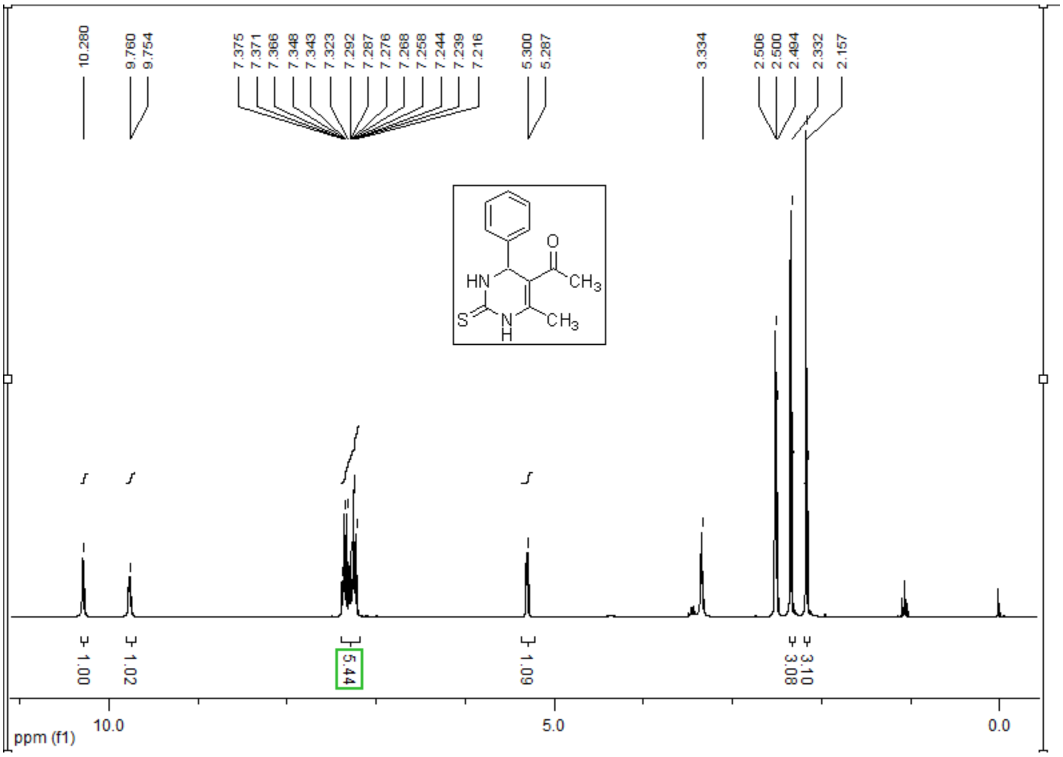
10.280
9.760
9.754
7.375
7.371
7.366
7.348
7.343
7.323
7.292
7.287
7.276
7.268
7.258
7.244
7.239
7.216
5.300
5.287
3.334
2.506
2.500
2.494
2.332
2.157
HN
S
N
H
O
CH3
CH3
1.00
1.02
5.44
1.09
3.08
3.10
ppm (f1)
10.0
5.0
0.0

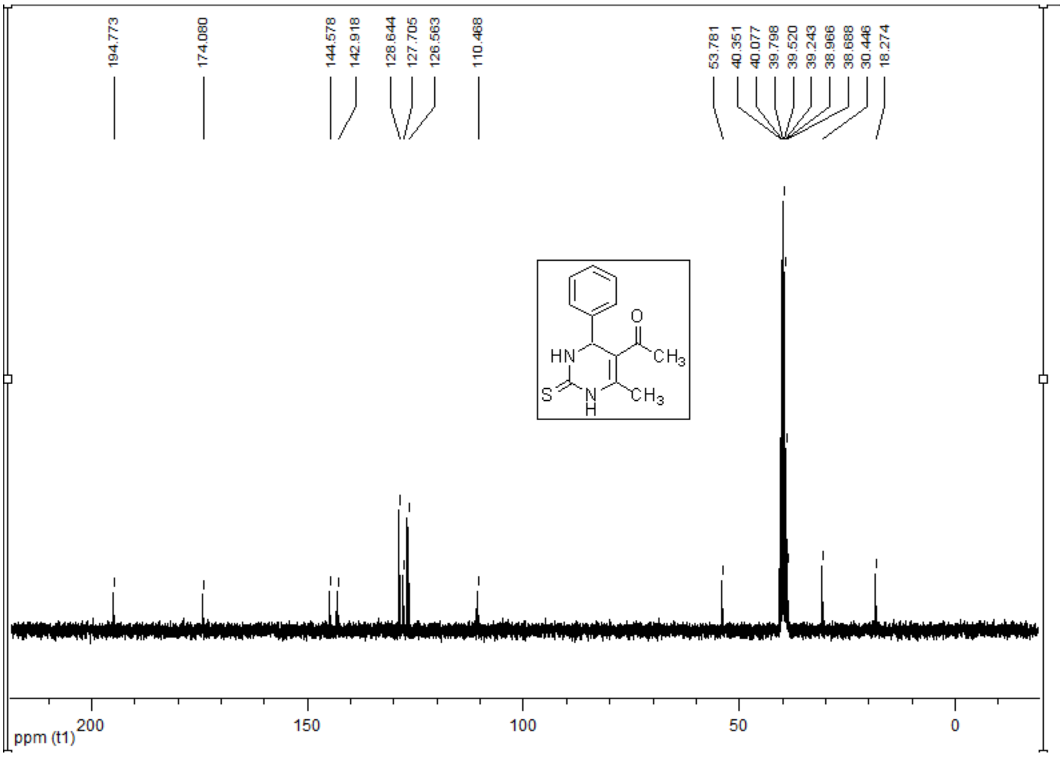
194.773
174.080
144.578
142.918
128.644
127.705
126.563
110.468
53.781
40.351
40.077
39.798
39.520
39.243
38.966
38.688
30.446
18.274
HN
S
N
H
O
CH3
CH3
ppm (t1)
200
150
100
50
0

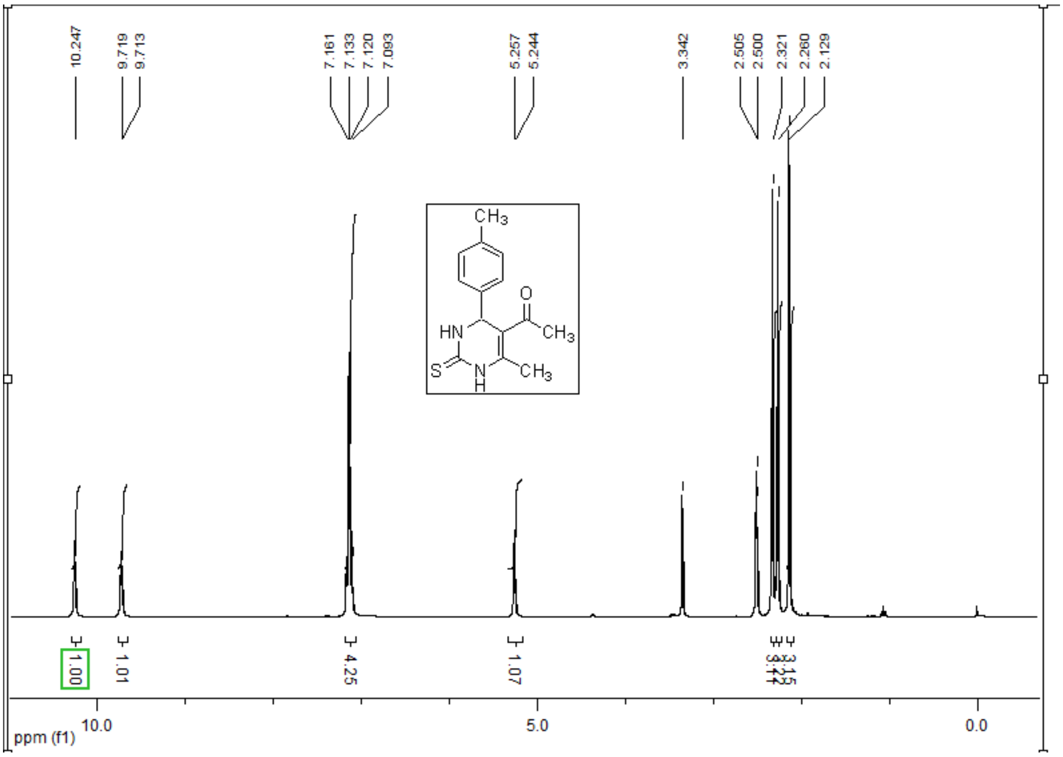
10.247
9.719
9.713
7.161
7.133
7.120
7.093
5.257
5.244
3.342
2.505
2.500
2.321
2.260
2.129
CH3
O
CH3
HN
S
N
H
CH3
1.00
1.01
4.25
1.07
3.15
ppm (f1)
10.0
5.0
0.0

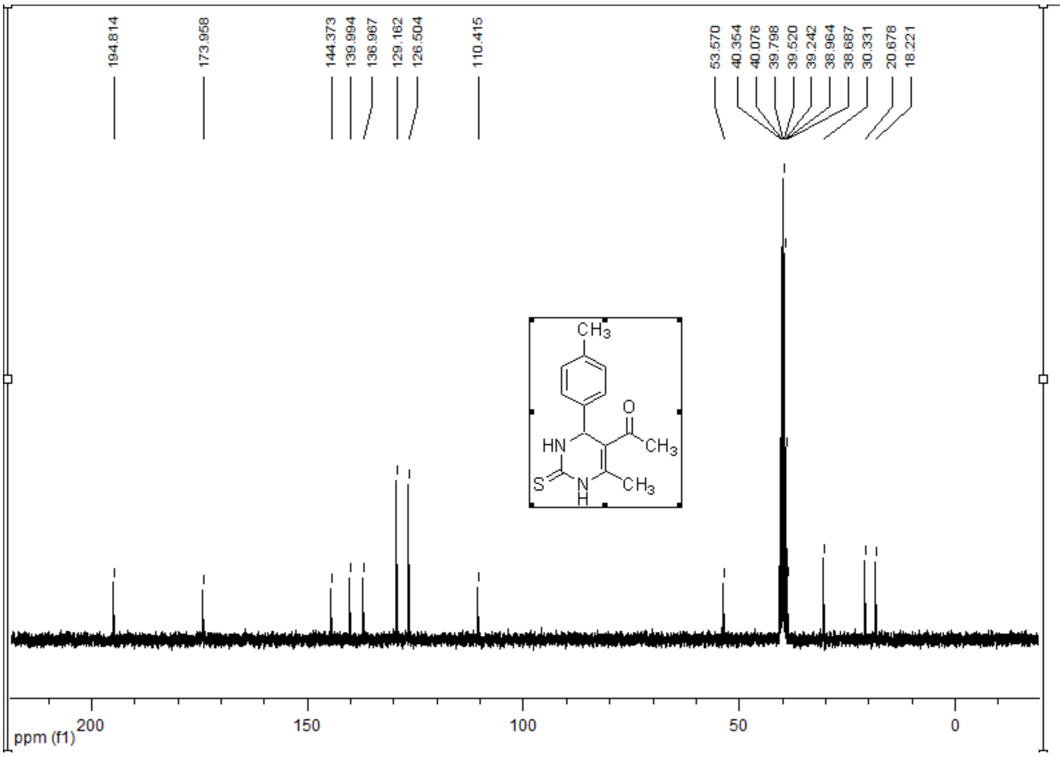
194.814
173.958
144.373
139.994
136.967
129.162
126.504
110.415
53.570
40.354
40.076
39.798
39.520
39.242
38.964
38.687
30.331
20.678
18.221
CH3
O
CH3
HN
S
N
H
CH3
ppm (f1)
200
150
100
50
0

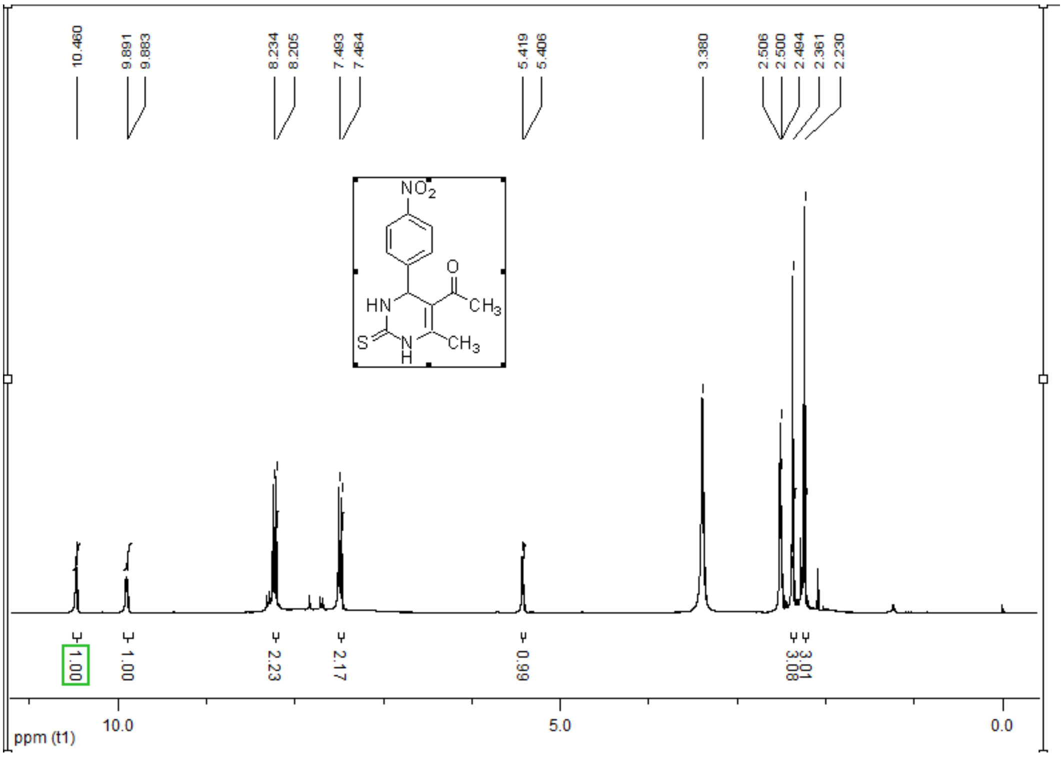
10.460
9.891
9.883
8.234
8.205
7.493
7.464
5.419
5.406
3.380
2.506
2.500
2.494
2.361
2.230
1.00
1.00
2.23
2.17
0.99
3.01
3.08
10.0
5.0
0.0
ppm (t1)

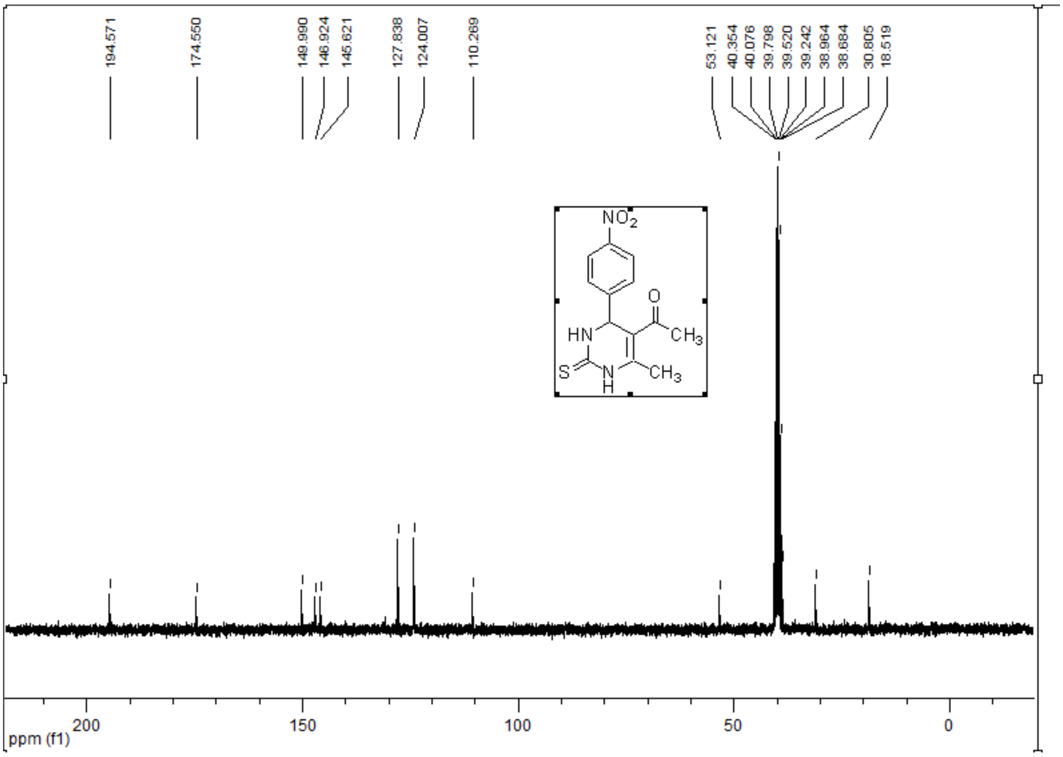
194.571
174.550
149.990
146.924
145.621
127.838
124.007
110.269
53.121
40.354
40.076
39.798
39.520
39.242
38.964
38.684
30.805
18.519
200
150
100
50
0
ppm (f1)
